# Detection of structural DNA variants in medulloblastomas using optical genome mapping

**DOI:** 10.1186/s40478-026-02245-7

**Published:** 2026-02-11

**Authors:** Nadezhda Kubon, Mirela Bălan, David Koppstein, Sophia Praeger, Marietta Wolter, Peter Ebert, David Pauck, Jörg Felsberg, Thomas Beez, Daniel Picard, Marc Remke, Guido Reifenberger

**Affiliations:** 1 Institute of Neuropathology, Medical Faculty and University Hospital Düsseldorf, Heinrich Heine University, Moorenstr. 5, 40225 Düsseldorf, Germany; 2 Department of Rheumatology, Medical Faculty of Heinrich Heine University, University Hospital Düsseldorf, Düsseldorf, Germany; 3 Hiller Research Center, Medical Faculty of Heinrich Heine University, University Hospital Düsseldorf, Düsseldorf, Germany; 4 Department of Pediatric Oncology, Hematology and Clinical Immunology, Medical Faculty and University Hospital Düsseldorf, Heinrich Heine University, Düsseldorf, Germany; 5 Cancer Bioinformatics and Multiomics (ED08) German Cancer Research Center (DKFZ), Heidelberg, Germany; 6 German Cancer Consortium (DKTK), partner site Essen/Düsseldorf, Düsseldorf, Germany; 7 Core Unit Bioinformatics (CUBI), Medical Faculty and University Hospital Düsseldorf, Heinrich Heine University, Düsseldorf, Germany; 8 Center for Digital Medicine, Heinrich Heine University, Düsseldorf, Germany; 9 Department of Neurosurgery, Medical Faculty and University Hospital Düsseldorf, Heinrich Heine University, Düsseldorf, Germany; 10 Department of Pediatric Hematology and Oncology, University Medical Center of Saarland, Homburg, Saar Germany

**Keywords:** Pediatric brain tumors, DNA copy number profile, Medulloblastoma groups, Optical genome mapping, Structural variants

## Abstract

Medulloblastoma (MB) is the most common malignant brain tumor in children that is subdivided into distinct MB groups characterized by specific genetic, epigenetic, and transcriptional profiles. In addition to single nucleotide variants, structural variants (SVs), including DNA deletions, duplications, focal gene amplifications, translocations and inversions, may contribute to MB pathogenesis. However, comprehensive profiling of SVs is challenging, as established cytogenetic methods such as karyotyping and fluorescence *in situ* hybridization (FISH) are limited by low resolution or restricted to detection of predefined SVs. Moreover, commonly used molecular diagnostic techniques like gene panel next-generation sequencing and microarray-based DNA copy number (CN) analysis may not reveal complex SVs. To more comprehensively characterize SVs in MB, we applied optical genome mapping (OGM) for genome-wide SV detection in 29 MB tissues and six MB cell lines. We found that OGM reliably detected genome-wide DNA CN changes in these samples, as validated by comparison to microarray-based DNA CN profiling. In addition, OGM allowed for the characterization of novel genomic alterations, including amplification and overexpression of the *GPC5* gene on chr13q31.3 in a Group 3 MB tumor, as well as recurrent SVs affecting the *NRXN1* gene on chr2p16.3 by deletion or translocation in subsets of MB Group 3 and 4 tumors. Taken together, our data indicate that OGM represents a promising approach for comprehensive profiling of SVs in pediatric brain tumors.

**Supplementary Information:**

The online version contains supplementary material available at 10.1186/s40478-026-02245-7.

## Introduction

Medulloblastoma (MB) is the most common embryonal tumor of the central nervous system (CNS) that preferentially develops in infants and children but may also present in adolescents and young adults [28, 40]. Four major molecular MB groups have been distinguished based on distinct transcriptional profiles and DNA methylation patterns, namely wingless (WNT)-activated MB (MB WNT), sonic hedgehog (SHH)-activated MB (MB SHH), and non-WNT/non-SHH-activated MB (MB non-WNT/non-SHH), further stratified into MB Group 3 and MB Group 4 tumors [28, 40]. Genome-wide DNA methylation profiling further refined MB classification, enabling segregation of MB subclasses, including two subclasses of MB WNT, four subclasses of MB SHH and eight subclasses within the MB non-WNT/non-SHH tumors [28, 40]. Thus, MB represents a highly heterogeneous disease with different molecular characteristics leading to diverse clinical manifestations and outcomes.

In contrast to solid cancers in adults that usually present with elevated mutational burden, studies on different types of pediatric solid tumors reported lower mutational loads but a relatively high prevalence of structural variants (SVs) of ≥ 50 bp in size [29, 55]. Thus, different mechanisms leading to tumor initiation and progression of adult *versus* pediatric tumors have been postulated [29, 55]. While single nucleotide variants (SNVs) leading to loss-of-function or activating mutations of tumor suppressor genes or proto-oncogenes are well-documented in different MB groups [39], characterization of SVs is mostly limited to detection of copy number variants (CNVs) including numerical aberrations of whole chromosomes or chromosome (chr) arms as well as circumscribed gene amplifications or homozygous deletions [39, 47]. Generally, SVs are classified as insertions, inversions, translocations, deletions, duplications, and other CNVs [34]. They contribute not only to interindividual diversity of the human genome but are also detectable as somatic genetic events in cancer cells [33, 36]. In different types of cancers, SVs involving tumor suppressor genes or proto-oncogenes have been identified as relevant tumor drivers [60]. However, large-scale profiling for SVs remains challenging, as traditional cytogenetic methods such as karyotyping and fluorescence *in situ *hybridization (FISH) are limited by low resolution and thus restricted to detecting either very large (5–10 Mbp) or predefined SVs (one probe, one target), respectively. Short-read next-generation sequencing (NGS) techniques are variably able to detect SVs but have limited sensitivity due to the short read length (~ 150 bp), which also affects the accuracy of the read alignment to the reference genome. This becomes particularly challenging in genomic regions with highly repetitive sequences or regions with multiple overlapping SVs, as the sequence alterations in such regions are difficult to capture or amplify, and may not easily be mapped to the reference genome [12, 22].

To assess the role of structural DNA variants in MB, we extracted ultra-high molecular weight DNA (UHMW DNA) from fresh-frozen tumor tissues of 29 MB patients, corresponding fresh-frozen blood samples of 18 patients, and 6 long-term established MB cell lines, and performed optical genome mapping (OGM) [6, 10]. OGM is based on an enzymatic reaction that places fluorescence-labeled DNA probes at a specific sequence motif throughout the genome. The fluorescence-labeled UHMW DNA molecules are then linearized in nanochannel arrays, and changes in the patterning or spacing of the labels are being identified. Thereby, OGM provides a high-resolution genome-wide map highlighting DNA copy number (CN) and structural anomalies from 500 base pairs (bp) to one or more megabase pairs (Mbp) in length [9]. It detects simple SVs including deletions, insertions, inversions, duplications, and translocations as well as complex genomic rearrangements or chromothripsis events [9]. In comparison to short-read NGS, OGM is able to align to larger segments of the genome and may better cover repetitive regions.

We found that OGM reliably identified genome-wide DNA CN changes in MBs with high concordance to DNA methylation microarray-based CN profiling. In addition, OGM enabled the characterization of novel genomic alterations including focal amplifications, deletions and chromosomal translocations affecting cancer-relevant genes. These included a circumscribed SV on chr13q31.3 that led to amplification and overexpression of the glypican 5 (*GPC5*) gene. Furthermore, we identified recurrent SVs affecting the *NRXN1* gene on chr2p16.3 in non-SHH/non-WNT MBs, and detected novel potentially oncogenic gene fusions.

## Materials and methods

### Patients’ samples

Unfixed fresh-frozen tumor tissue samples from patients with different subtypes of medulloblastoma (MB) were retrieved from the CNS tumor tissue bank at the Department of Neuropathology, Heinrich Heine University Düsseldorf, Germany. In total, MB tumors from 29 patients (18 male, 11 female, median age: 7 years, range 1–42 years), including 3 MB WNT, 11 MB SHH, 4 MB Group 3, and 11 MB Group 4 tumors, were analyzed for genome-wide SVs (Table S1). Constitutional DNA samples from peripheral blood leukocytes were available from 18 of the 29 patients (Table S1). The study was approved by the Institutional Review Board of the Medical Faculty, Heinrich Heine University Düsseldorf (study number: 2021-1745_1).

### Cell lines and culture conditions

In addition to primary tissue and blood samples from MB patients, OGM analyses were performed for six human MB cell lines. MB cell lines D283 Med (RRID:CVCL_1155), MED-MEB-8A (RRID:CVCL_M137), ONS-76 (RRID:CVCL_1624) and UW228-3 (RRID:CVCL_0573) were provided by Dr. Pablo Landgraf (Department of Pediatric Oncology and Hematology, Children's Hospital, University of Cologne, Germany), while HD-MB03 (RRID:CVCL_S506) was provided by Prof. Dr. Till Milde (Department of Pediatrics and Adolescent Medicine, Jena University Hospital, Jena, Germany), and Daoy (RRID:CVCL_1167) was obtained from the American Type Culture Collection (ATCC, Manassas, Virginia, USA). Cell lines were incubated in a humidified atmosphere at 37 °C and 5% CO_2_. The cell line MED-MEB-8A was grown in Roswell Park Memorial Institute (RPMI) 1640 medium (RPMI 1640, Gibco™, Thermo Fisher Scientific, Waltham, MA, #31870025) supplemented with 10% fetal bovine serum (FBS, Sigma-Aldrich, St. Louis, MO, #F9665, GibcoTM, Thermo Fisher Scientific) and 1% MEM non-essential amino acids (MEM NEAA, Gibco™, Thermo Fisher Scientific, #11140035). Daoy was grown in minimal essential medium (MEM, Gibco™, Thermo Fisher Scientific, #10370047) supplemented with 10% FBS and 1% penicillin/streptomycin (P/S, Sigma-Aldrich, #P4333). The cell lines D283 Med, HD-MB03, ONS-76, and UW228-3 were grown in Dulbecco’s Modified Eagle’s medium (DMEM, Thermo Fisher Scientific, #31966021) supplemented with 10% FBS and 1% P/S. Authentication of the cell lines was conducted by STR profiling and each cell line was regularly tested for mycoplasma contamination.

### Ultra-high molecular weight DNA extraction

Ultra-high molecular weight (UHMW) DNA was extracted from unfixed frozen tissue samples using the Bionano Prep SP Tissue and Tumor DNA Isolation Protocol (#30339; Bionano Genomics, San Diego, CA, USA). Only solid cellular tissue specimens with a histologically estimated tumor cell content of 80% or more (based on microscopic assessment of cryo-sections stained for hematoxylin-eosine) were used for DNA extraction. Tissue samples of approximately 3 mm^3^ (corresponding in weight to the 3–12 mg range recommended by Bionano) were retrieved by manual tissue punching. UHMW DNA from blood was extracted according to the Bionano Prep SP Frozen Human Blood DNA Isolation Protocol v2 (#30395, Rev B). The Bionano Prep SP Frozen Cell Pellet DNA Isolation Protocol (#30268) was used for extraction of UHMW DNA from MB cell lines. Quantification of the purified UHMW DNA was performed spectrophotometrically using the QuantiFluor® ONE dsDNA System (Promega, Madison, WI, USA) and the Quantus™ Fluorometer (Promega).

### Optical genome mapping (OGM)

A total of 750 ng UHMW DNA from each sample was fluorescently labelled, loaded on a Saphyr chip (G2.3, Bionano Genomics), and DNA molecules were imaged by the Saphyr instrument (Gen 2, Bionano Genomics). Details on DNA labelling and OGM analysis are provided as Supplementary Information.

### Data collection and detection of structural variants and copy number variants

Each flow cell of the Saphyr chip generated an average of 1312 gigabase pairs (Gbp) of sequence data per labelled UHMW DNA sample and multiple cycles of imaging generated numerous single-molecule maps to reach the average effective coverage of 343.0x (± 37.8x).

For each analyzed sample, one raw molecule file (BNX format) was generated and the human reference genome GRCh38 was used for alignment. For further specimen-specific QC metrics refer to Supplementary Table S2.

Each molecule file was analyzed with two Bionano-integrated genome assembly algorithms, the Rare Variant Analysis (RVA) Pipeline and the De Novo Assembly Pipeline (DNP) (Figure S1). Prior to DNP analysis, molecule files were subjected to downsampling to 480 Gbp with a minimal molecule length of 200 kilobase pairs (kbp), as recommended by Bionano Genomics. In cases in which OGM data from matched constitutional DNAs were available, the “Dual” Variant Annotation Pipeline (VAP) was applied to automatically exclude germline SVs. SV and CNV calling, automatic variant annotation and visualization were performed on the Bionano Access Server (version 1.7, Bionano Genomics) using Bionano Solve (version 3.7). More details on the pipeline analyses and SV pre-filtering are provided as Supplementary Information.

### Structural variant annotation and interpretation

In addition to the Bionano-integrated SV pre-filtering and annotation, the pre-filtered SV datasets were manually compared with the University of California Santa Cruz (UCSC) Genome Browser (GENCODE, release 42, GRCh38.p13, https://www.gencodegenes.org/human/release_42.html). We also reviewed whether the identified SVs overlap with (i) genes from the Cancer Gene Census of the Catalogue Of Somatic Mutations In Cancer (COSMIC CGC, https://cancer.sanger.ac.uk/census; v98), (ii) exonic regions of genes not included in the COSMIC CGC, and (iii) putative polymorphic regions > 1% of allele frequency using the TCAG DGV Curated Catalogue of Human Structural Variation (https://dgv.tcag.ca/dgv/docs/GRCh38_hg38_variants_2020-02-25.txt) and NCBI dbVAR Curated Common SVs (https://www.ncbi.nlm.nih.gov/dbvar/studies/nstd186/).

### RNA sequencing

Total RNA was extracted from fresh-frozen MB tissue samples (n = 29) using the Maxwell® RSC simplyRNA Tissue Kit and Maxwell® RSC Instrument (Promega). RNA sequencing libraries were prepared either with the Illumina Stranded Total RNA Prep, Ligation with Ribo-Zero Plus kit (Illumina, San Diego, CA, USA) or the TruSeq RNA Sample Preparation v2 kit (Illumina) according to the manufacturer’s instructions. Quality of RNA extracts and the generated libraries was assessed using an Agilent Bioanalyzer (Agilent Technologies, Santa Clara, CA). Sequencing of the libraries was performed for 101 cycles using either the NextSeq2000 (Illumina) or the NovaSeq6000 (Illumina) sequencer. For detection and visualization of gene fusions, Arriba [53] (v.2.4.0) was used with the references provided by Arriba for GRCh38 and Ensembl v104 gene model. For additional information regarding gene fusions, we deployed FusionCatcher [38] (v1.33) together with the Human Ensembl v102 annotation release. Further details on the RNA sequencing data processing workflow, visualization and gene fusion calling are provided as Supplementary Information.

### Infinium™ MethylationEPIC bead chip-based DNA methylation profiling

The 29 MB tumor samples and the six MB cell lines were subjected to DNA methylation microarray-based profiling using hybridization to Infinium™ MethylationEPIC BeadChip v1.0 (Illumina, San Diego, CA) arrays. DNA methylation data were analyzed with the Heidelberg Epignostix CNS Tumor Classifier v12.8 (https://app.epignostix.com/#/classifiers), and the tumors and cell lines were accordingly assigned to methylation families, classes and subclasses based on calibrated classifier scores (Table S1). In addition to DNA methylation-based classification, genome-wide DNA copy number (CN) information was obtained from the DNA methylation dataset and compared to CNVs identified by OGM. Details on the DNA methylation microarray-based profiling workflow and the comparison of CN profiles obtained either by OGM or by DNA methylation microarray-based analyses are provided as Supplementary Information.

### Immunohistochemistry

For selected cases with high-level copy number gains (gene amplification) of *CCND2* or *MYCN*, immunohistochemical stainings of FFPE tissue samples were performed using a Dako Autostainer Link 48 immunostainer (Agilent Technologies, Santa Clara, CA). Cyclin D2 rabbit polyclonal antibody (Proteintech Group Inc., Rosemont, IL, 10934-1-AP) and N-Myc (D4B2Y) rabbit monoclonal antibody (Cell Signaling Technology Europe B.V., Leiden, The Netherlands, #51705) were used at 1:250 and 1:100 dilutions, respectively. Antigen binding of the primary antibodies was detected with the EnVision FLEX system (Agilent Technologies) using 3,3′-diaminobenzidine as horseradish peroxidase substrate and chromogen. Sections were counterstained with hemalum.

### Statistical analyses and data visualization

Statistical analyses were performed using R (v.R-4-3-2, https://cran.r-project.org/) with the RStudio interface (https://posit.co/downloads/) [42]. The following publicly available packages were downloaded in RStudio and applied accordingly: dplyr, ggplot2 [56], ggpubr, tidyverse [57], multcompView, rstatix, and stats. All data were presented as mean (± SD) unless stated otherwise. Comparisons between groups were made by one-way ANOVA and *p* values < 0.05 were considered statistically significant. The Tukey’s honest significance post hoc test (HSD) was carried out for pairwise comparisons of means between examined groups.

Summarized visualization of OGM-detected SVs in single samples was done using circos plot view and specific SVs were shown in the Genome Browser view within the Bionano Access Software (Bionano). Individual circos plots of the MB cell lines and all MB tumor samples are shown in Supplementary Figure S7. For cumulative visualization of OGM-detected SVs in multiple samples of the same MB groups, the Circa.Ink software (https://circa.omgenomics.com) was applied. An overview of the CNV profiles was created with the OncoPrinter webtool (https://www.cbioportal.org/oncoprinter).

## Results

### DNA methylation microarray-based profiling of MB tumors and cell lines

DNA methylation microarray-based profiling using the Heidelberg Epignostix CNS Tumor Classifier v12.8 (https://app.epignostix.com/#/classifiers) assigned all MB tumors to one of the three medulloblastoma methylation families (MB WNT, MB SHH, MB non-WNT/non-SHH), with classifier scores ≥ 0.9 obtained in 25/29 cases (Table S1). Twenty-one MB tumors were further assigned to distinct MB methylation (sub)classes based on calibrated classifier scores ≥ 0.9: “Medulloblastoma, WNT-activated” (MB WNT, n = 3), “Medulloblastoma, SHH-activated, subclass 1 or 2” of the infant subgroup (MB SHH–infant, n = 5), “Medulloblastoma, SHH-activated, subclass 3 or 4” of the non-infant subgroup (MB SHH–child, n = 1), “Medulloblastoma, non-WNT/non-SHH, Group 3 subtype, subclass I–IV” (MB Group 3, n = 3), and “Medulloblastoma, non-WNT/non-SHH, Group 4 subtype, subclass V–VIII” (MB Group 4, n = 9) (Table S1). Eight MB tumors were not assigned to any of the MB methylation (sub)classes with a classifier score ≥ 0.9, however, they could also be stratified based on classifier scores between 0.44 and 0.86 (Table S1). The HD-MB03 cell line was assigned to the MB non-WNT/non-SHH methylation family with a MF classifier score of 0.68 and the MB non-WNT/non-SHH methylation subclass II with a MF classifier score of 0.53 (Table S1). For the five remaining MB cell lines, no classifier scores of ≥ 0.3 were obtained, and these cell lines could therefore not be assigned to any specific MB methylation (sub)class (Table S1).

### Detection of DNA copy number variants by OGM

Following molecular classification of the MB tumors into MB methylation (sub)classes, we examined the genome-wide DNA CN profiles as detected by OGM with respect to CNVs and patterns of group-dependent CN alterations. All OGM-identified CN segments without detected alterations within the range of 1.800 ≤ CN ≤ 2.200 were considered as balanced and were excluded from the CNV call-set. The CN segments that were explicitly listed within the OGM CNV call-set were assigned a fractional CN value (Table S3, “fractCN”) and defined as “gain” with 2.200 < CN ≤ 3.500, “amplification” with CN > 3.500, “hemizygous deletion” with CN < 1.800, and “homozygous deletion” with CN < 0.800. Alternating CN values assigned to different segments within the same chromosome (e.g. CN = 2.223, CN = 1.646 and CN = 2.834) were classified as a “complex” CN signatures. In line with Bierkens et al. [8], CN alterations were further distinguished according to their size as follows: “focal” CNV for sizes < 3 Mbp and “partial” CNV for sizes > 3 Mbp. Whole-chromosome aneuploidies, such as monosomy or trisomy, were assigned when calculated fractional CN size was ≥ 0.630 and aneuploidies of whole chromosomal arms were assigned upon calculated fractional CN size ≥ 0.315. Detailed CNV data for each MB tumor and cell line are listed in Table S3 and depicted in Fig. S7.

**Fig. 1:**
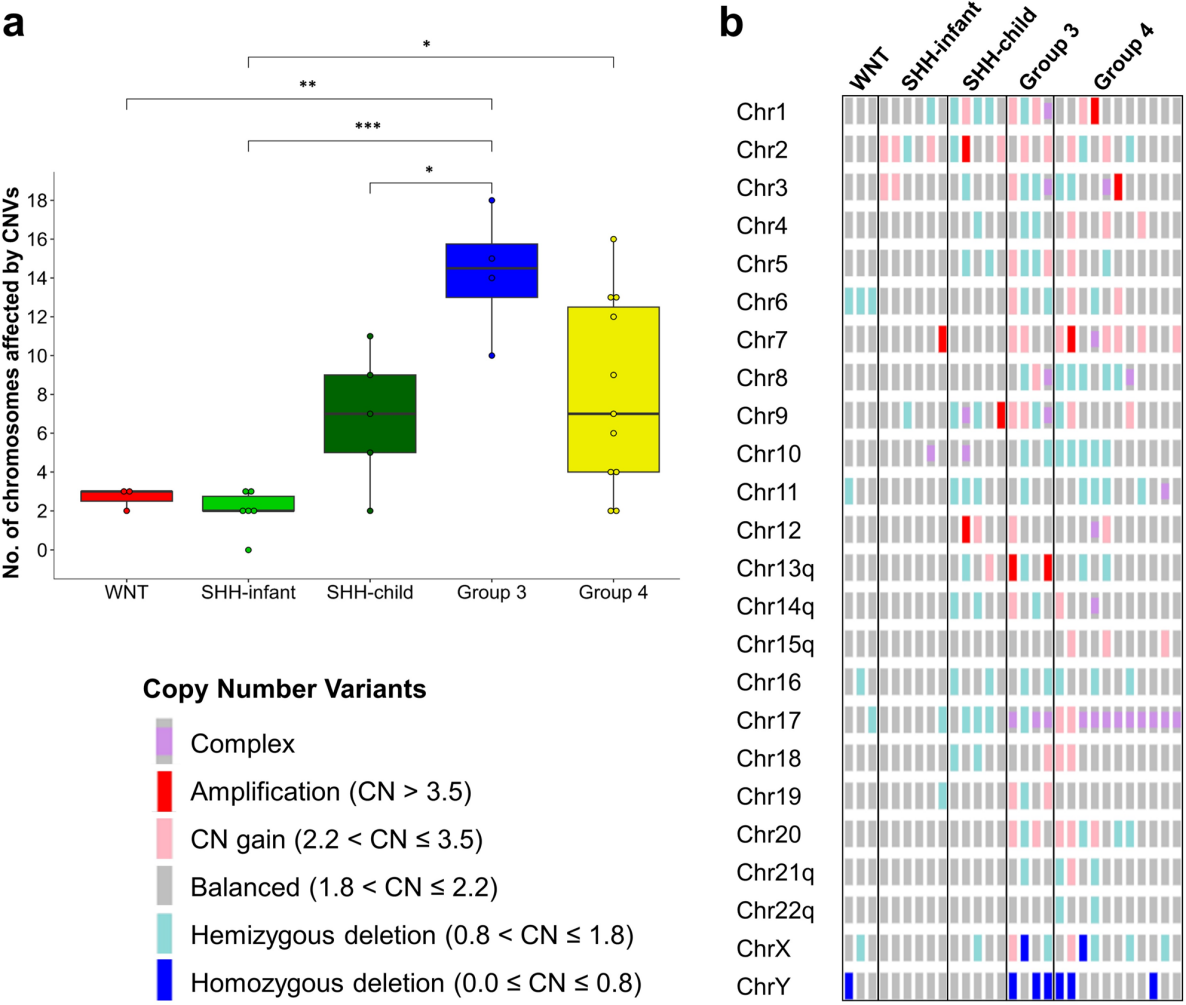
DNA copy number variants (CNVs) detected by OGM in 29 MB tumors. (**a**) Group-wise comparison of the number of chromosomes affected by CNVs using ANOVA followed by Tukey’s test: *p < 0.05, **p < 0.01; ***p < 0.001. The individual MB groups are color-coded as follows: MB WNT, red; MB SHH–infant, light green; MB SHH–child, dark green; MB Group 3, blue; MB Group 4, yellow. Note that MB Group 3 showed the highest CNV load with significant differences to the MB WNT and MB SHH groups. **(b)** Simplified overview of the OMG-detected CNVs in MB tumors sorted by MB groups. Each column represents one MB tumor. Different types of CNVs are color-coded as illustrated in the lower left of the figure.

The CNV prevalence, here defined as number of chromosomes affected by CNVs per tumor, was significantly higher in MB Group 3 tumors CNVs (14.25 ± 3.30) as compared to MB WNT, MB SHH–infant, and MB SHH–child tumors (Fig. 1a). No significant difference in CNV prevalence was found between MB Group 3 and MB Group 4 tumors.

An overview of CNVs detected by OGM (Fig. 1b) shows that MB WNT tumors carried a low number of CNVs (2.67 ± 0.58), with monosomy of chr6 present in all three analyzed cases. In addition, monosomy of chrX and chr11, and loss of chrY were observed once in each. The overall lowest CNV prevalence (2.00 ± 1.10) was detected in SHH–infant MBs (Fig. 1a), in which two out of six tumors presented trisomy of chr2 and chr3 (Fig. 1b). Otherwise, tumor genomes of SHH–infant MBs were mostly balanced with only few focal CNVs (Fig. 1a, b). In contrast, multiple focal/partial CNVs were detected by OGM in MB Group 3, with CN patterns consistent with isochromosome 17q in three out of four cases (Fig. 1b, indicated as “Complex”). In MB SHH–child tumors, 6.80 (± 3.49) chromosomes were affected by CNVs per tumor, including frequent large CNVs on different chromosomes. One MB SHH–child presented a CNV pattern consistent with isochromosome formation of 9p and 10p (Fig. 1b, indicated as “Complex”). The highest intra-group heterogeneity of CNVs was found in MB Group 4 (8.00 ± 4.90), with chr17 being invariably affected, including isochromosome 17q in 9 of 11 cases, and trisomy chr17 in the remaining two cases. In addition, many complex CNVs were detected in MB Group 4 tumors as sub-chromosomal CN alterations or concomitant whole arm gain and loss of individual chromosomes suggestive of isochromosome formation.

### Comparison of DNA copy number variants detected by OGM or DNA methylation microarray-based analyses

**Fig. 2:**
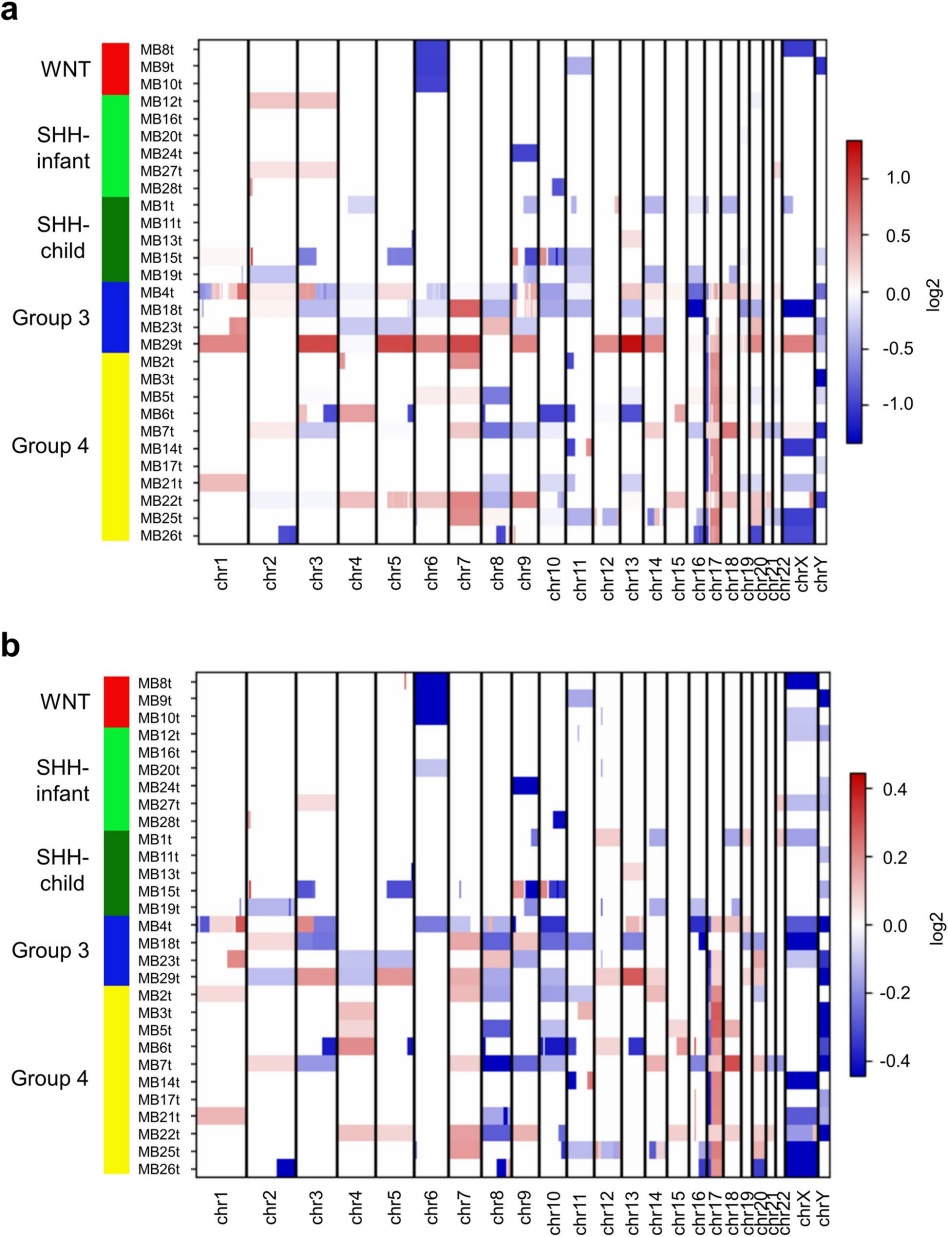
Comparison of DNA copy number profiles detected by OGM (**a**) or by DNA methylation microarray-based analysis (**b**) in 29 MB tumors. The individual tumor samples are listed on the y-axis stratified according to MB group. The individual MB groups are color-coded as follows: MB WNT, red; MB SHH–infant, light green; MB SHH–child, dark green; MB Group 3, blue; MB Group 4, yellow. Genome-wide CNVs are stratified from chr1 to chrY on the x-axis. Segments of losses and gains are color-coded according to their relative log2 copy number ratios. The log2 median segment intensity ranges from 1 to -1, and 0.4 to -0.4, respectively. Note a high degree of overlap in the pattern of CNVs detected by either method.

To validate the CNVs detected by OGM in MB tumors we compared the OGM CN profiles with those derived from the DNA methylation microarray-based analyses. Figure 2a summarizes the OGM-derived CN profiles of the investigated 29 MB cases and Fig. 2b presents the summary of the corresponding CN profiles obtained by the DNA methylation microarray-based analyses. Compared to the DNA methylation microarray-based CN profiles, the OGM profiles presented a higher resolution segmentation of CNVs leading to detection of additional CN changes in individual cases (Fig. 2a, b; Fig. S7). These segmentation differences were most apparent in MB Group 3 and MB Group 4 cases, affecting e.g. chr1, chr3, chr6, chr9 and chr17 (MB4t, Fig. 2a, b). In two MB SHH–infant cases, MB12t and MB27t, low-level gains of chr2 were detected by OGM, but missed by the DNA methylation microarray-based analysis (Fig. 2a, b).

For quantitative comparison of both CNV detection methods, we assessed the similarity between the CN profiles by application of the “CNVmetrics” package [7]. Thereby, pairwise overlap metrics were calculated with the Sorensen similarity coefficient (twice the genomic intersection divided by the sum of each sample’s genomic ranges; see Supplementary Methods) using the CNV status calls from both methods for each MB sample. In 11/29 MB samples, the Sorensen coefficients were ≥ 0.9 (Fig. S2a). A heatmap of the Sorensen metrics of the 29 MB profiles showed that most OGM-CN profiles cluster closely to the respective DNA methylation microarray-based CN profiles (Fig. S2b). Furthermore, clustering of CN profiles based on biological group similarity was observed, as for instance in MB WNT (Fig. S2b: cluster in the lower right corner including MB9t, MB10t and MB8t) and most MB Group 3 tumors (Fig. S2b: cluster in the upper left corner including MB29t, MB4t and MB18t). Overall, we found a concordance of 86.2% for CNVs that were detected by both OGM and DNA methylation microarray-based analyses. Two cases, MB11t and MB16t, were excluded from the overall calculation of concordance because they presented balanced genomes without any CNVs detected by OGM or DNA methylation microarray-based analyses (Fig. S2a).

### Detection of structural DNA variants by OGM

**Fig. 3:**
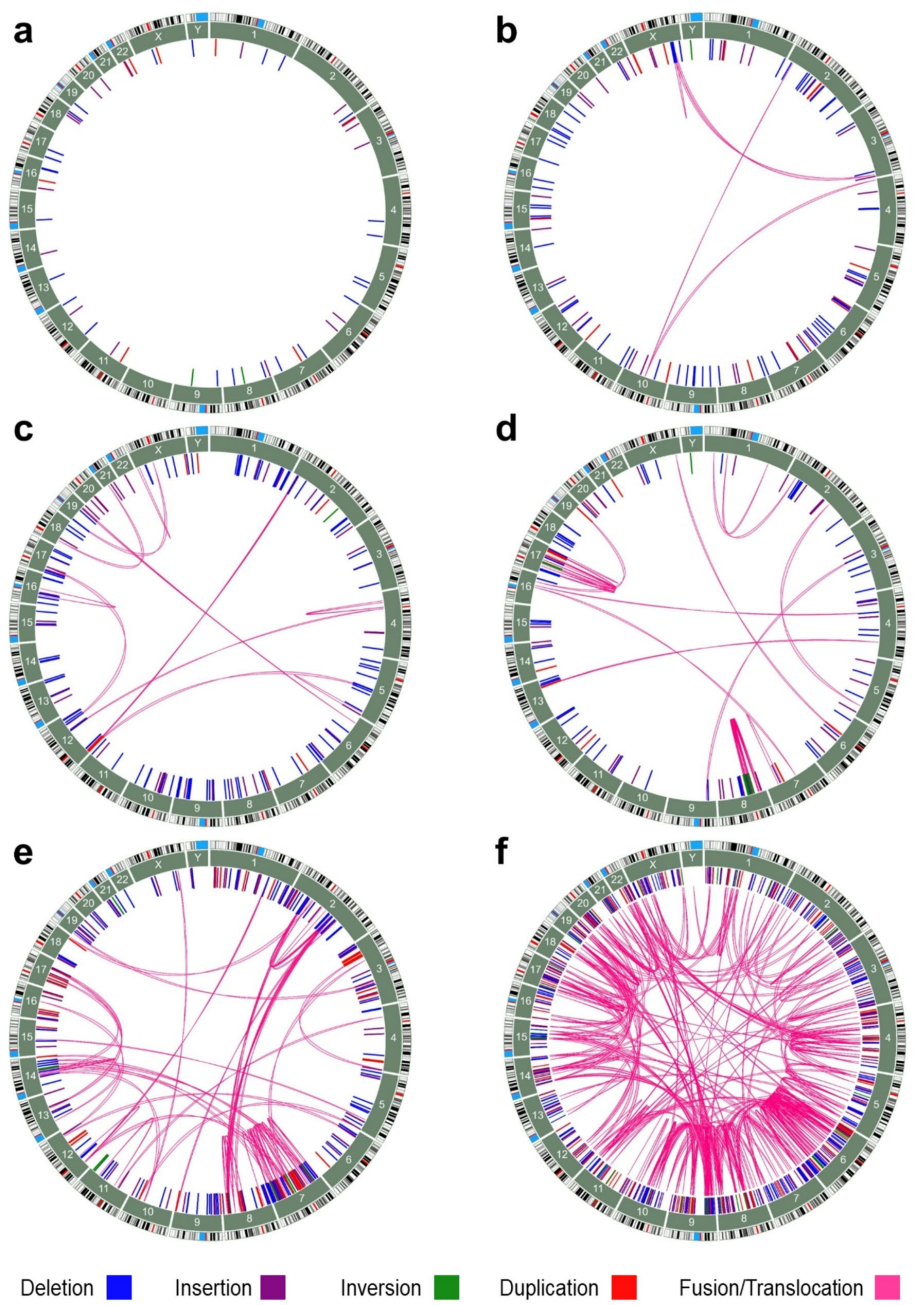
Cumulative SV profiles detected by OGM in 29 MB tumors stratified according to the distinct MB groups (**a**–**e**) and in six MB cell lines (**f**). The outer circle of each plot shows the ideogram of the respective chromosome with the centromere labelled in red and gaps in light-blue according to the GRCh38 reference genome. The numbered rectangles in the circle further inside represent each chromosome. SVs (deletions in blue, insertions in purple, inversions in green, and duplications in red) are visualized as lines inside the circle indicating the involved genomic positions. Complex SV events (translocations, chromothripsis) are shown as pink arcs connecting either the same (intrachromosomal SVs) or two different chromosomes (interchromosomal SVs). (**a**) MB WNT (n = 3 tumors, SVs = 57), (**b**) MB SHH–infant (n = 6 tumors, SVs = 125), (**c**) MB SHH–child (n = 5 tumors, SVs = 224), (**d**) MB Group 3 (n = 4 tumors, SVs = 188), (**e**) MB Group 4 (n = 11 tumors, SVs = 349) and (**f**) MB cell lines (n = 6 cell lines, SVs = 1476).

In total, 53 samples were processed in the OGM workflow (Tables S1, S2 and S3). OGM-integrated SV calling algorithms identified significantly more SVs in the MB cell lines as compared to the MB tumors, i.e., 1512.79 (± 217.82) SVs per tumor vs. 1812.67 (± 332.67) SVs per cell line (*p* = 0.011) (Table S4a, b; Fig. S3a). Overall, deletions and insertions contributed to the majority of identified SVs (Fig. S3c, e; Table S4a, b). Only a minority of SV calls (0.5%) in MB tumors corresponded to translocations (Fig. S3c; Table S4b), while translocations were more common in the MB cell lines (8.1%) (Fig. S3e; Table S4b). Rare SVs were retained after exclusion of common SVs, which were defined by the allele frequency cut-off value of ≤ 1% among the Bionano-integrated control database of more than 200 healthy individuals. Again, the overall fraction of rare SVs remained higher in MB cell lines as compared to MB tumors (*p* < 0.0001) (Table S4a, b; Fig. S3b). The rare SVs detected in MB tumors are summarized as cumulative SV profiles stratified according to MB group and MB cell lines in Fig. 3. In the three analyzed MB WNT tumors, 57 unique, rare SVs were identified (Fig. 3a, Table S4b). Among others, these included a hemizygous deletion covering the *CBFB*, *CTCF* and *CHD1* gene loci on chr16 (chr16:66,886,992–69,305,254; ~ 2.4 Mbp) in MB8t (Fig. S7), and a hemizygous deletion on chr17p (chr17:7,286,494–7,840,671; ~ 0.5 Mbp) spanning the *TP53* gene locus in MB10t (Fig. S7).

In the six investigated MB SHH–infant cases, 125 unique, rare SVs including deletions, duplications, insertions, inversions and translocations were detected by OGM (Fig. 3b). Four SVs affected exonic regions of cancer-related genes such as *STAG2* (a hemizygous deletion of ~ 7 Mbp at chrX:123,995,348–130,952,538 and a chr3::chrX translocation: t(3;X)(3q28;Xq25), *NPIPB2* (an approximately 5 kbp deletion at chr16:11,917,019–11,962,114) and *NOTCH2* (an approximately 6 kbp insertion at chr1:119,960,225–119,969,033). Two MB SHH–infant tumors, MB20t and MB28t, carried distinct interchromosomal translocations. In MB20t, chr3 and chrX were involved, and in MB28t, chr2, chr4 and chr10 (Fig. S7). Recurrent SVs were not found among the MB SHH–infant tumors.

The five MB SHH–child tumors presented an overall higher abundance of SVs compared to the MB WNT and MB SHH–infant tumors, and an increased complexity of structural genomic rearrangements. In total, 224 unique, rare SVs were detected (Fig. 3c). Multiple SVs affected exonic regions of cancer-associated genes in individual tumors, such as *RPL5* (hemizygous deletion of ~ 2.4 Mbp at chr1:92,491,106–94,918,675) and *SET*, *FNBP1*, *ABL1*, *TSC1* (hemizygous deletion of ~ 4.6 Mbp at chr9:128,376,902–132,947,376) in MB1t, *FOXP1* (~ 5 kbp deletion at chr3:71,465,253–71,476,913) in MB11t, *RGS7*, *FH*, and *AKT3* (hemizygous deletion of ~ 4.4 Mbp at chr1:241,009,879–245,374,670) in MB13t, and *ABL2* (~ 5 kbp insertion at chr1:179,201,431–179,219,468) in MB19t (Fig. S7). In two cases (MB15t and MB1t), large deletions at chr9 (chr9:94,811,772–95,606,427; chr9:95,392,140–98,655,497) were detected that covered the *FANCC* and *PTCH1* loci in MB15t, and *PTCH1* and *XPA* loci in MB1t.

**Fig. 4:**
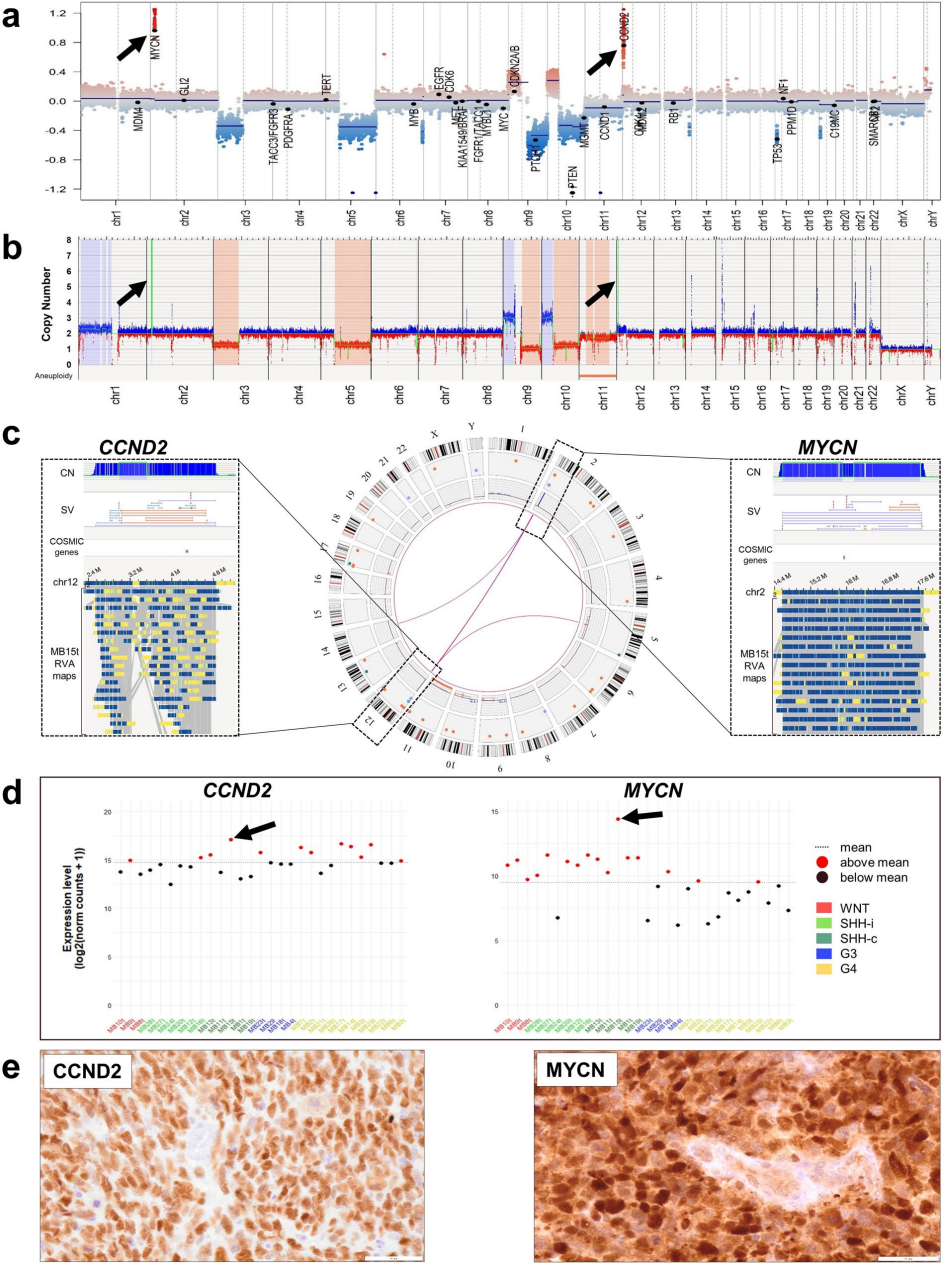
Overview of SVs and selected genes in the MB SHH–child tumor MB15t. (**a**) Whole genome CN profile of MB15t as detected by DNA methylation microarray-based analysis with losses (blue) and gains (red) of chromosomal regions. *y-axis*, log2 median segment intensity in the interval [-1.2, 1.2]; *x-axis*, chr1–22 plus sex chromosomes. (**b**) Corresponding whole genome CN profile of MB15t as detected by OGM showing similar losses (red) and gains (blue) of chromosomal regions. *y-axis*, CN in the interval [0, 8]; *x-axis*, Aneuploidy indicators showing whole-chromosome monosomies or total losses (red) and whole-chromosome trisomies or amplifications (blue), and chr1–22 plus sex chromosomes. (**c**) Circos plot of OGM findings in MB15t showing from the outside in the individual chromosomes with their ideograms, SVs as color-coded dots (*red*, deletion; *purple*, duplication; *light-blue*, insertion), large CN alterations, and translocations/events of complex rearrangements as pink arcs. Translocation events between chr5 and chr12, as well as chr2 and chr12 were detected. The latter translocation colocalized with two amplified regions on chr2 and chr12. The boxes flanking the circos plot display a more detailed view of the amplified regions that included the proto-oncogenes *CCND2* on chr12 (left) and *MYCN* on chr2 (right). (**d**) *CCND2* (left plot) and *MYCN* (right plot) mRNA expression levels calculated based on RNA sequencing data across all analysed MB tumors and sorted by MB groups. The increased *CCND2* and *MYCN* mRNA expression levels in MB15t are indicated by black arrows. *y-axis*, (log2(norm counts + 1)) in the interval [0, 15]; *x-axis*, individual MB cases separated by group: MB WNT in red, MB SHH in green, MB Group 3 in blue and MB Group 4 in yellow. (**e**) Immunohistochemical staining of CCND2 (left) and MYCN (right) shows positive and strong protein expression in MB15t tumor cells.

MB15t (Fig. S7) displayed a particularly high SV load affecting multiple loci of cancer-associated genes including *CCND2, MYCN, FGFR10P, AFDN, FANCC, PTCH1, PTEN, FAS, YWHAE, TP53* and *MAP2K2*. Notably, this case contributed to two thirds of all translocations detected in this MB group (Fig. 3c and Fig. S7). For example, a chr2::chr12 translocation t(2;12)(p24.2;p13.32) associated with amplification of *MYCN* on chr2 and amplification of *CCND2* on chr12, yielding gene copy numbers of 19 and 140, respectively (Fig. 4a–c, Table S3). RNA sequencing and immunohistochemical staining supported the enhanced mRNA and protein expression of both amplified genes (Fig. 4d, e).

**Fig. 5:**
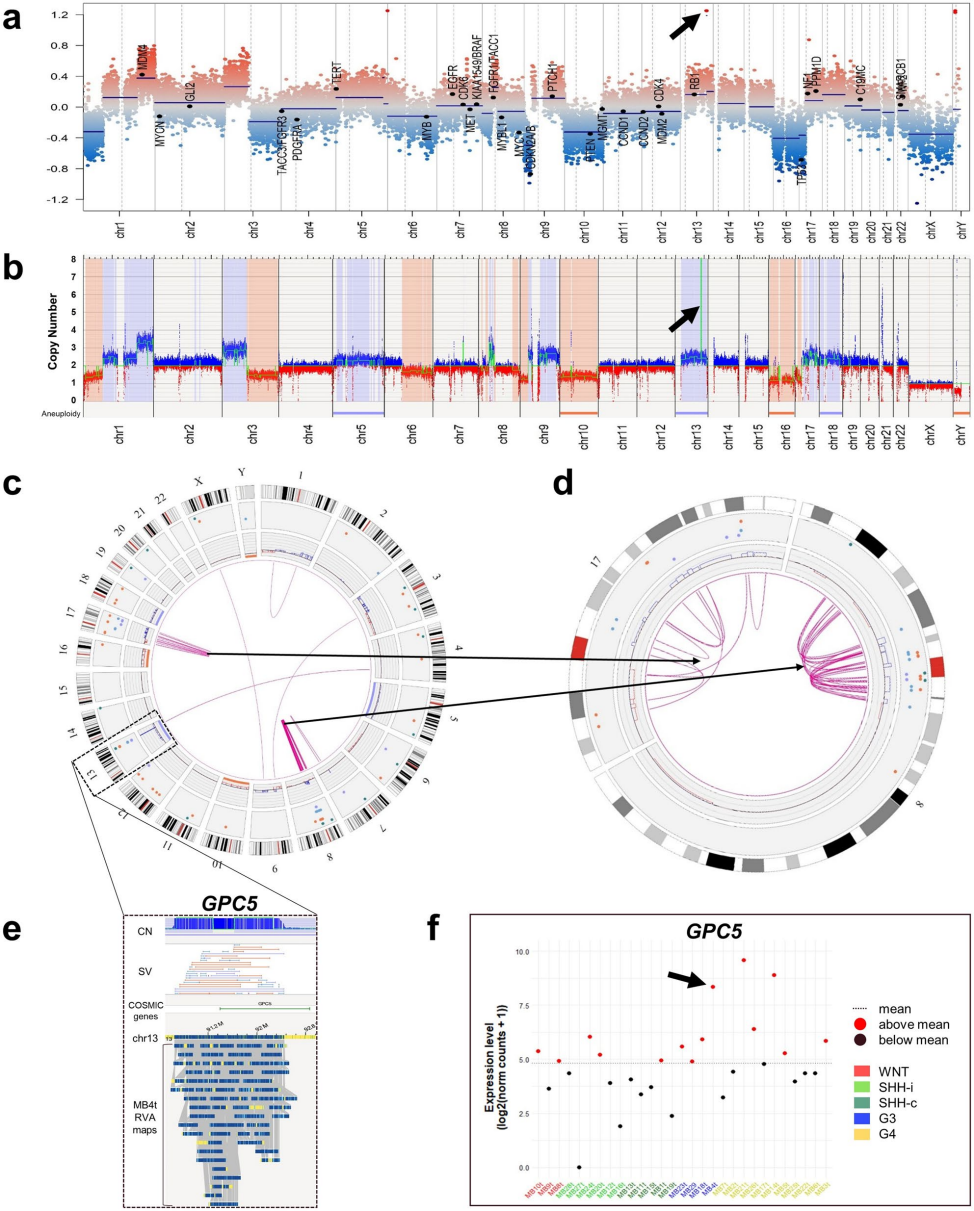
Overview of SVs and selected genes in the MB Group 3 tumor MB4t. (**a**) Whole genome CN profile of MB4t as detected by DNA methylation microarray-based analysis with losses (blue) and gains (red) on different chromosomes. *y-axis*, log2 median segment intensity in the interval [-1.2, 1.2]; *x-axis*, chr1–22 plus sex chromosomes. (**b**) Corresponding whole genome CN profile as detected by OGM with losses (red) and gains (blue) of similar chromosomal regions. *y-axis*, CN in the interval [0, 8]; *x-axis*, Aneuploidy indicators showing whole-chromosome monosomies or total losses (red) and whole-chromosome trisomies or amplifications (blue), and chr1–22 plus sex chromosomes. Many chromosomes show CNVs and an isochromosome 17q is present. Arrows in (a) and (b) point to a circumscribed high-level copy number gain corresponding to *GPC5* gene amplification on chr13. (**c**) A circos plot of MB4t shows from the the outside in, the chromosome ideograms, SVs as color-coded dots (*red*, deletion; *purple*, duplication; *light-blue*, insertion), large CN alterations, and translocations/events of complex rearrangements as pink arcs. Two clusters of such complex rearrangements are detected on chr8 and chr17, as shown in more detail in the circos plot (d) for both chromosomes. In addition, a high-level CN gain/amplification of a 1,580,887 bp region on chr13 (chr13:90,684,670–92,265,557) including the *GPC5* locus is present. (**d**) Enlarged view of the highly amplified region detected by OGM on chr13 including *GPC5*. (**e**) *GPC5* mRNA expression levels across all MB tumors sorted by groups. The increased *GPC5* expression in MB4t is indicated by an arrow. Expression levels were calculated based on RNA sequencing data. The three tumors with increased *GPC5* mRNA levels are indicated by red dots between 7.5 and 10.0. *y-axis*, (log2(norm counts + 1)) in the interval [0, 10]; *x-axis*, individual MB cases separated by group: MB WNT in red, MB SHH in green, MB Group 3 in blue and MB Group 4 in yellow.

In the four MB Group 3 tumors (MB4t, MB18t, MB23t, MB29t), 188 unique, rare SVs were detected (Fig. 3d), most of which were present in MB4t (Fig. 5). This particular tumor presented multiple different SVs on chr13 (chr13q31.1) leading to a focal amplification (CN = 19) of the cancer-associated gene *GPC5* (Fig. 5e, Table S3), which in turn led to enhanced *GPC5* mRNA expression levels, as demonstrated by RNA sequencing (Fig. 5f). In addition, multiple intrachromosomal translocations were detected on chr8 and chr17, suggesting complex chromosomal rearrangements like chromothripsis (Fig. 5c, d). In MB23t, a SV affected the exonic region of the cancer-associated gene *ATRX* (~ 5 kbp hemizygous deletion at chrX:77,507,310–77,524,034), and in MB29t a hemizygous deletion of *CDKN2A* (~ 275 kbp at chr9:21,923,701–22,208,558) was detected (Fig. S7). No recurrent SVs were found among the MB Group 3 tumors.

In the 11 MB Group 4 tumors, 349 unique, rare SVs of varying complexity were detected (Fig. 3e). Although several cancer-related genes were affected, only few recurrent SV regions were observed mostly affecting intronic or intergenic genomic regions. Recurrent SVs among two MB Group 4 cases (MB21t and MB26t) and three MB Group 3 cases (MB4t, MB18t and MB29t) clustered on chromosome band 2p16.3 (Table S5). The deletions overlapped with either *NRXN1* or *NRXN1-DT* introns, while in MB18t an *MSH2* intron was affected. Deletions overlapping with *NRXN1* or *NRXN1-DT* were also observed in the MB cell lines D283 Med and UW228-3, where both exonic and intronic genomic regions were affected (Table S5). In the Daoy cell line, *NRXN1* was affected by an interchromosomal chr2::chr7 translocation t(2;7)(p16.3;q36.3) (Fig. S7).

Regarding complex genomic events or translocations, three MB Group 4 tumors MB5t, MB21t, and MB26t featured intra- and interchromosomal rearrangements affecting chromosome arm 2q (q22.2, q22.3). Additionally, MB21t and MB26t presented complex rearrangements affecting regions on chromosome arm 8q (q21.13, q24.13, q24.21).

In the MB patients with matched tumor and blood samples, exclusion of SVs found in both tumor and blood assemblies revealed tumor-specific somatic SVs. The previously defined rare SV dataset was thereby significantly reduced (Tables S4a, b; Fig. S4). Although these were rare SVs, a larger number of deletions and insertions were in fact germline changes and hence excluded from the final list of somatic SVs. In contrast, all of the intrachromosomal fusions, interchromosomal translocations and most of the inversions and duplications were solely found in tumors, and were thus of somatic origin. This information allowed us to review and reassess those MB where the respective blood samples were not available. The translocations identified in this manner were included in the final list of somatic SVs and likely reflect somatic gene fusions (Table S6).

### Validation of gene fusions detected by OGM using RNA sequencing

**Fig. 6:**
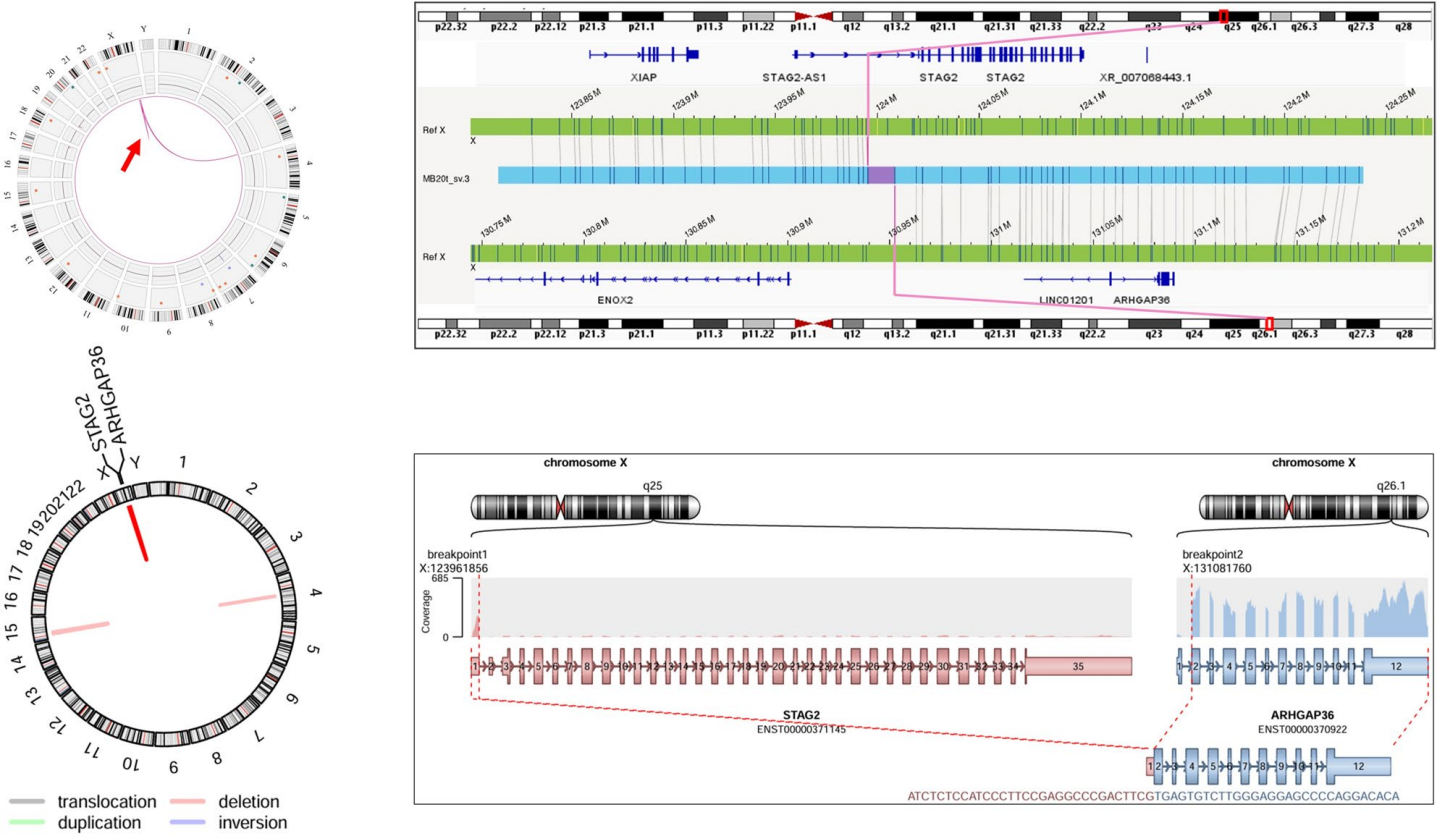
Detection of a *STAG2::ARHGAP36* fusion in the MB SHH–infant tumor MB20t. Two representations of the same gene fusion event are given. The upper circos plot (left) and SV-view (right) are derived from OGM data and the lower circos plot (left) and SV-view (right) from Arriba analysis of RNA sequencing data. The circos plots emphasize all structural rearrangements of the analyzed sample. The SV event of interest is indicated by a red arrow (OGM) or a red line (Arriba), and both SV-views zoom into the specific SV event. In OGM-SV-view, the reference regions are indicated in green with the respective whole chromosome above and below, respectively. The light blue OGM consensus map (map incorporating the SV) is shown in the middle and illustrates the partial alignment to the reference regions, reflecting an intrachromosomal translocation within the q-arm of chrX. The breakpoints are within the *STAG2* gene and an intergenic region. In the Arriba-SV-view (lower panel), a schematic visualization of detected transcripts of the fusion partners, their coverage, orientation and the retained exons contributing to the fusion transcript are visualized, corresponding to ENST0000371145.8(STAG2):e.1::ENST0000370922.5(ARHGAP36):e.2_12 fusion transcript formed by an intrachromosomal deletion in chrX.

RNA sequencing was performed for validation of putative gene fusions resulting from somatic SVs as detected by OGM (Table S6). In 26 of 29 analyzed MB tumors, 62 high-confidence gene fusions between two gene partners were detected using Arriba [53]. Most of these high-confidence gene fusions resulted from deletions (n = 23), followed by duplications (n = 21), translocations (n = 9) and inversions (n = 9). Upon comparison of Arriba-detected fusions with the final list of OGM-detected rare SVs, several fusion events detected by Arriba and labelled as “high-confidence”, overlapped with OGM-detected SVs (Table S6). These include novel, potentially relevant gene fusions in MB that were detected by OGM, and validated by RNA sequencing using both Arriba and FusionCatcher [38] (Table S7). In addition to our own cohort, we investigated RNA sequencing data from two publicly available MB datasets [1, 21] for fusion events using the Arriba and FusionCatcher pipelines. These analyses revealed that the *STAG2::ARHGAP36* fusion detected by OGM and RNA sequencing in a single MB SHH–infant tumor (MB20t) of our cohort (Fig. 6, Table S7) was also detectable in a single case of the Forget et al. [21] cohort (Table S7).

For the other gene fusions identified in our cohort, we could not detect them in the two independent MB RNA sequencing datasets, potentially indicating that they might be novel gene fusions in MB (Figs. S5a–e; Table S7). In another single MB SHH–infant tumor (MB28t), a translocation-dependent t(4;10)(p16.3;q21.3) out-of-frame *SH3BP2::CTNNA3* fusion was identified (Fig. S5a). Among the SHH–child tumors, two gene fusions were found: a deletion-dependent del(12)(p13.1p12.3) out-of-frame *ATF7IP::PTPRO* fusion in MB15t and a deletion-dependent del(5)(q35.1q35.3) in-frame *RANBP17::CANX* fusion in MB13t (Fig. S5c). Two gene fusions, an *ITGA9-AS1::EIF1B-AS1* fusion (Fig. S5D) and an out-of-frame *MYRIP::ITGA9* fusion (Fig. S5e), were found in a single Group 4 tumor (MB5t) resulting from the same duplication-dependent SV event dup(3)(p22.1p22.2).

## Discussion

Previous studies have demonstrated the use of OGM to detect SVs with higher resolution and sensitivity than classical cytogenetic methods like karyotyping and fluorescence in situ hybridization [23, 35]. However, OGM has mostly been used for CNV and SV detection in hereditary disorders [44, 48] and hematological malignancies [13, 23, 32], with relatively few studies focusing on solid tumors [2, 5, 24]. OGM analyses of brain tumors are sparse, even though its utility has been demonstrated in pilot studies on a small cohort of gliomas [49] and a single case of a pediatric embryonal CNS tumor [11].

Here, we applied OGM for detection of genome-wide structural DNA rearrangements in 29 MB tumors alongside established molecular profiling techniques, such as DNA methylation microarray-based analysis and RNA sequencing. DNA methylation microarray-based profiling allows for molecular classification of brain tumors and simultaneous identification of CN profiles [14, 18, 20], while RNA sequencing provides transcriptional profiles and detection of gene fusion events [27, 36, 53]. Comparison of OGM and DNA methylation microarray-based analyses for detection of CNVs revealed an 86.2% concordance between the two methods. In individual cases, OGM identified additional CNVs that were not apparent on the DNA methylation microarray-based CN profiles (e.g. in MB12t and MB27t, Fig. 2a, b) and appeared to be more specific in the segregation of distinct, consecutive CNV segments (e.g. in MB4t, Fig. 2a, b).

Both, OGM and DNA methylation microarray-based analyses revealed differences in CN profiles between the distinct MB groups [15, 28]. OGM additionally identified translocations, complex SVs and putative gene fusion events. Visualization of individual SVs helped to reveal how distinct genetic events are interconnected and contribute to complex genomic rearrangements like chromothripsis. The number of SVs per genome was higher in MB cell lines as compared to MB primary tumors, reflecting the highly rearranged genomes in these long-term cultivated MB models. Among the primary tumors, MB Group 3 tumors showed more frequent SVs as compared to MB WNT and MB SHH tumors, reflecting their higher degree of genomic instability [40]. The four MB Group 3 tumors analyzed by OGM all presented complex SV patterns with multiple CN changes, including isochromosome 17q formation. Interestingly, we detected an amplification of the *GPC5* gene with accompanied increased levels of *GPC5* transcripts in one MB Group 3 tumor. *GPC5* encodes Glypican 5, a member of the glypican family of cell surface heparan sulfate proteoglycans (HSPG) [45]. These transmembrane proteins are documented to act as co-receptors in developmental cell signaling pathways and have been implicated in regulation of cellular growth, differentiation and modulation of growth factors [45]. Activating mutations or genomic alterations leading to overexpression of glypicans have been associated with developmental abnormalities and tumor progression, e.g., changes in *GPC3* expression, a paralog of *GPC5*, may induce cell growth and simultaneously inhibit cell differentiation in pediatric embryonal tumors [41]. Amplification and overexpression of *GPC5* has been documented in several adult and pediatric tumors [17], and has been linked to increased cell proliferation in rhabdomyosarcoma [58]. Similarly, GPC5 may promote growth of salivary adenoid cystic carcinoma (SACC) [61], while a study on uterine sarcomas reported no difference in cell proliferation but alteration in cell differentiation upon *GPC5* overexpression [17]. In non-small cell lung cancer, however, *GPC5* has been reported as a tumor suppressor, indicating that GPC5 function is dependent on context and tumor type [25]. Notably, two MB Group 4 tumors of our cohort also showed increased *GPC5* transcript levels (Fig. 5e), but no *GPC5* amplification by OGM, indicating that other mechanisms may contribute to increased *GPC5* expression in MB.

MB Group 4 is the most common molecular group accounting for approx. 40% of all MBs [40]. This group displayed the overall highest level of SV and CNV heterogeneity, in line with previous studies [40, 47, 52]. In addition to isochromosome 17q formation, another common feature consisting of multiple different SVs affecting 2p16.3 was identified among several cases of MB Group 4 and MB Group 3 tumors. Similar SVs were also present in the D283 Med, Daoy, and UW228-3 cell lines. Most of these SVs were deletions that overlapped either *NRXN1* or *NRXN1-DT* introns, while one tumor, MB21t, featured a translocation-dependent t(2;8)(p16.3;q23.1) *NRXN1::NUDCD1* putative gene fusion. Germline deletions/mutations affecting the *NRXN1* gene are the cause of the *NRXN1* deletion syndrome, a rare neurodevelopmental disease associated with a variable clinical phenotype including developmental delays, intellectual disability, and epilepsy [54, 59]. However, the role of *NRXN1* alteration in tumorigenesis is largely unexplored. *NRXN1* encodes Neurexin-1, a presynaptic cell-surface receptor that is critical for neuronal development and function [51]. Thus, inactivation of NRXN1 due to gene deletion or gene fusion may impair proper neuronal differentiation, and thereby potentially support tumorigenesis [51]. A recent study on colorectal cancer proposed *NRXN1* as a tumor suppressor gene and associated NRXN1 loss of expression with decreased patient survival [4]. In addition, the same study reported that NRXN1 inactivation promotes an undifferentiated cellular state, allowing tumor cells to retain proliferation, undergo epithelial–mesenchymal transition and thereby enhance their metastatic potential [4]. Our findings thus could point to a link between 2p16.3 SVs affecting *NRXN1* and tumorigenesis in a subset of MB non-WNT/non-SHH tumors.

In line with previous research [28, 40], MB WNT tumors presented with mostly balanced genomes upon OGM analysis. These tumors frequently show chr6 monosomy [28, 40], and overall demonstrate a favorable clinical course under current treatment [16]. In contrast, MB SHH tumors are associated with an intermediate prognosis that is subgroup-dependent with MB SHH–infant patients showing a better prognosis compared to patients with MB SHH–child tumors [16, 46]. This segregation is underlined by different SV and CNV profiles, as detected by OGM. In particular, the MB SHH–infant cases demonstrated a lower frequency of CNVs per tumor and the observed SVs were less complex, i.e., CNVs more commonly affected whole chromosomes without additional complex rearrangements or only single translocations in otherwise mostly balanced genomes. One of these translocations was detected in MB20t as an intrachromosomal exon–exon fusion on chrX, del(X)(q25q26.1), implicating the genes *STAG2* and *ARHGAP36*. *STAG2* encodes the STAG2 cohesin complex component that is an integral element of the cohesion complex crucial for chromosomal segregation during cell division [26]. STAG2 is considered as a tumor suppressor and *STAG2* alterations have been associated with increased rates of chromosomal aneuploidy and accelerated tumor progression [31, 50]. However, in line with reports on individual *STAG2*-mutant cancers that lacked obvious chromosomal aneuploidies [3], MB20t did not present evidence for genomic instability. *ARHGAP36* in turn encodes the Rho GTPase Activating Protein 36 that activates SHH signaling [37]. As ARHGAP36 has been documented to enhance transcriptional activation of GLI [43], the observed increased expression of *ARHGAP36* mRNA could potentially foster SHH signaling in MB20t, thereby contributing to tumorigenesis.

In summary, OGM allowed for genome-wide characterization of SVs in MB and revealed both previously documented as well as novel SVs, some of which likely contributing to MB growth. Examples include amplification and overexpression of the known MB-associated oncogenes *MYCN* and *CCND2* and a newly characterized amplification and overexpression of *GPC5* in individual tumors. We also found recurrent SVs affecting chr2p16.3 in MB Group 3 and Group 4 tumors, with *NRXN1* as a putative target gene of interest. Furthermore, several novel gene fusions potentially implicated in MB pathogenesis were identified by OGM and independently validated by RNA sequencing.

Nevertheless, OGM also has certain limitations in comparison to other methods, such as fluorescence in situ hybridization (FISH) as well as short-read NGS and long-read sequencing, that might be relevant for routine application in brain tumor diagnostics, including restriction to detection of SV sizes of > 500 bp, lack of sequence-level resolution, and higher sample quality requirements for extraction of UHMW DNA, i.e., unfixed tissue samples. However, other novel techniques for molecular brain tumor classification, in particular Oxford Nanopore™ Technologies (ONT) long-read sequencing, also work best with high molecular weight DNA extracted from unfixed tissue samples. Hence, combination of OGM and ONT sequencing may provide a comprehensive overview on CNV and SV profiles in brain tumors, including complex structural alterations, combined with diagnostic SNVs and methylation profiles [19, 30]. In conclusion, our study provides a proof of principle to support OGM as a novel molecular cytogenetic technique for detection of genome-wide SVs and CNVs in brain tumors in a single experiment and without the need for intricate bioinformatics workflows.

## Supplementary Information

Below is the link to the electronic supplementary material.

Supplementary Material 1.

Supplementary Material 2.

## Data Availability

Molecular data sets from this study are available from the corresponding author upon reasonable request.

## Abbreviations

bp: Base pairs
chr: Chromosome
CN: Copy number
CNS: Central nervous system
CNV: Copy number variant
DNP: De Novo Assembly Pipeline
FISH: Fluorescence in situ hybridization
Gbp: Gigabase pairs
kbp: Kilobase pairs
MB: Medulloblastoma
Mbp: Megabase pairs
NGS: Next-generation sequencing
OGM: Optical genome mapping
PCR: Polymerase chain reaction
RVA: Rare variant analysis
SHH: Sonic hedgehog
SNV: Single nucleotide variant
SV: Structural variant
UHMW DNA: Ultra-high molecular weight DNA
VAP: Variant Annotation Pipeline
WHO: World Health Organization
WNT: Wingless
