# Supplementary material for: Detection of structural DNA variants in medulloblastomas using optical genome mapping
Source: Acta Neuropathol Commun. 2026 Feb 11;14:50. doi: 10.1186/s40478-026-02245-7 (PMC12930802; doi:10.1186/s40478-026-02245-7)
Supplement: Supplementary file 1 — Supplementary Material 1. [file 40478_2026_2245_MOESM1_ESM.pdf]

Detection of structural DNA variants in medulloblastomas using optical genome mapping

Nadezhda Kubon[1], Mirela Bălan[2,3], David Koppstein[4,5,6,8], Sophia Praeger[4,5,6,8], Marietta Wolter[1], Peter Ebert[7,8], David Pauck[1], Jörg Felsberg[1], Thomas Beez[9], Daniel Picard[10], Marc Remke[10], Guido Reifenberger[1,6]

[1]Institute of Neuropathology, Heinrich Heine University, Medical Faculty and University Hospital Düsseldorf, Düsseldorf, Germany
[2]Department of Rheumatology, University Hospital Düsseldorf, Medical Faculty of Heinrich Heine University, Düsseldorf, Germany
[3]Hiller Research Center, University Hospital Düsseldorf, Medical Faculty of Heinrich Heine University, Düsseldorf, Germany
[4]Department of Pediatric Oncology, Hematology and Clinical Immunology, Heinrich Heine University, Medical Faculty and University Hospital Düsseldorf, Düsseldorf, Germany
[5]Cancer Bioinformatics and Multiomics (ED08) German Cancer Research Center (DKFZ) Heidelberg, Germany
[6]German Cancer Consortium (DKTK), partner site Essen/Düsseldorf, Düsseldorf, Germany
[7]Core Unit Bioinformatics (CUBI), Medical Faculty and University Hospital Düsseldorf, Heinrich Heine University, Düsseldorf, Germany
[8]Center for Digital Medicine, Heinrich Heine University Düsseldorf, Germany
[9]Department of Neurosurgery, Heinrich Heine University, Medical Faculty and University Hospital Düsseldorf, Düsseldorf, Germany
[10]Department of Pediatric Hematology and Oncology, University Medical Center of Saarland, Homburg/Saar, Germany

SUPPLEMENTARY INFORMATION

Supplementary Materials and Methods

Ultra-high-molecular-weight (UHMW) DNA extraction

UHMW DNA was extracted from fresh frozen medulloblastoma (MB) tissue samples using the SP Tissue and Tumor DNA Isolation Kit Bionano Genomics, San Diego, CA, (#80038) according to the Bionano Prep SP Tissue and Tumor DNA Isolation protocol (Bionano Genomics, #30339). In detail, unfixed, fresh-frozen tumor tissue samples were transferred into a 15 mL conical tube containing 4 mL of pre-chilled homogenization buffer and homogenized for 20 sec at maximal speed using the T 25 digital ULTRA-TURRAX homogenizer (IKA Labortechnik, Staufen, Germany) with a pre-chilled mixing rod. The homogenizer mixing rod was rinsed with 6 mL of ice-cold homogenization buffer using a pipette and the buffer was collected in the same conical tube containing the cell homogenate. The homogenate was decanted through a 40 µm cell strainer (provided in the Bionano extraction kit) into a 50 mL conical tube and placed on ice. The 15 mL conical tube was rinsed with additional 5 mL chilled homogenization buffer to capture cells and debris from the sides of the tube and again decanted through the 40 µm cell strainer into the 50 mL conical tube on ice. The entire tumor cell homogenate (~ 15 mL) was mixed by pipetting up and down twice, transferred into a new 15 mL conical tube and pelleted at 2,000 x g for 5 minutes at 4°C. Subsequently, the supernatant was removed, the cell pellet resuspended in 1 mL of Wash Buffer A (Bionano Genomics) and the cell suspension was transferred into a pre-chilled 1.5 mL Protein LoBind tube (Eppendorf, Hamburg, Germany) for the final centrifugation step at 2,000 x g for 5 minutes at 4°C. Finally, the supernatant was removed, leaving approximately 40 µL for resuspension of the cell pellet. The cell suspension was treated with proteinase K (Bionano Genomics), and detergent-containing Lysis and Binding Buffer (LBB, Bionano Genomics) was added. The cell lysate was mixed using a HulaMixer (ThermoFisher Scientific, Waltham, MA) for homogenous release of DNA. Following PMSF (Sigma-Aldrich, St. Louis, MO) treatment, Salting Buffer, one Nanobind Disc (both from Bionano Genomics) and Isopropanol were added on top and the lysate was repeatedly mixed using a HulaMixer to allow for homogenous DNA binding to the Nanobind Disc. Four rounds of washing steps were followed by DNA elution in elution buffer (EB, Bionano Genomics), and final mixing using a HulaMixer for homogenization of UHMW DNA. To increase homogeneity of the freshly extracted DNA, each sample was mixed by pipetting up

and down with a wide-bore 200 µL tip and kept overnight at room temperature. UHMW DNA extraction from frozen MB cell line pellets and frozen blood aliquots was performed similarly, but with the SP Blood & Cell Culture DNA Isolation Kit (Bionano Genomics, #80030)" according to the Bionano Prep SP Frozen Cell Pellet DNA Isolation Protocol (#30268, Rev D) or the Bionano Prep SP Frozen Human Blood DNA Isolation Protocol v2 (#30395, Rev B).

Direct labelling of UHMW DNA

UHMW DNA extracted from MB cell line, MB tumor or peripheral blood samples was subsequently labelled by using the Bionano Prep DLS Labeling Kit (Bionano Genomics, #80005) as described in the Bionano Prep Direct Label and Stain (DLS) Protocol (#30206, Rev F). Briefly, 750 ng of DNA was treated with DL-green fluorophores using the Direct Labelling Enzyme 1 (DLE-1) that enables direct fluorescent labelling of double-stranded DNA at the CTTAAG sequence motif. This was followed by proteinase K (Qiagen, Hilden, Germany) digestion and DL-Green (Bionano Genomics) clean-up consisting of two membrane adsorption steps. Therefore, each DNA sample was pipetted onto a membrane and incubated in the dark for one hour at room temperature. After one hour, each DNA sample was carefully collected within a standard pipette tip, reapplied to a fresh membrane and re-incubated under the same conditions for 30 minutes. The DLE-1-labelled UHMW DNA samples were homogenized by mixing using a HulaMixer and DNA backbone counterstaining was performed protected from light overnight at room temperature. The labelled DNA was quantified in duplicates using the QuantiFluor® ONE dsDNA System (Promega, Fitchburg, WI). The quantified, labelled DNA samples were temporary stored at 4°C protected from light and finally equilibrated to room temperature directly before loading into the flow cells of the G2.3 Saphyr Chip (Bionano Genomics, #20366).

Structural variant (SV) and copy number variant (CNV) detection by optical genome mapping (OGM)

Each OGM raw molecule file of ~ 1300 Gbp was initially submitted to the Rare Variant Analysis (RVA) pipeline which is specifically designed to identify rare SVs at low variant allele frequencies

(VAF ≥ 5%), as for example in malignant cancers characterized by marked cellular heterogeneity giving rise to subclonal DNA variants. The SV detection of the RVA pipeline is based on direct overlapping alignments of few, raw single-molecules with the provided reference, whereas the labelling pattern of the DNA maps is compared and SVs of ≥ 5 kbp are detected with high sensitivity [3]. The annotated De novo Assembly Pipeline (DNP) was conducted in parallel to the annotated RVA pipeline. Here, each raw molecule file of ~ 1300 Gbp was filtered down to ~ 480 Gbp with a custom minimal molecule length of 200 kbp, corresponding to an approximate raw genome coverage of ~ 90x, as recommended by Bionano Genomics. The filtered molecule file was submitted to the DNP. In contrast to the RVA, the DNP initially builds a new, reference-independent genome map from the raw imaged single molecules by comparison and alignment of the labelling pattern of DNA maps, and thus requires less raw genome coverage with still sensitive detection of SVs ≥ 500 bp in a diploid genome [3]. The final genome assembly was then used for SV detection and annotation against the provided reference.

The annotated SVs from RVA and DNP were filtered with the following SV confidence scores: insertion = 0, deletion = 0, inversion = 0.7, duplication = -1, intra-fusion = 0.05, inter-translocation = 0.05, self-molecule count = 5. To exclude common SVs and polymorphisms, a custom variant annotation threshold was applied setting the SV occurrence cut-off to ≤ 1% among the Bionano-integrated control database of > 200 healthy individuals. For SVs detected by the RVA, we excluded all SVs with a variant allele frequency (VAF) < 0.05 and size < 4.5 kbp. Although the RVA-predefined minimal SV size was 5 kbp, the filtering SV minimum size was set to 4.5 kbp to ensure reliable detection of SVs, taken into account that the methods' minimal resolution is 500 bp [3]. This adaptation ensured that all SVs, which did not specifically reach the 5 kbp, were still included within the RVA call-set.

For identification of copy number variations (CNVs), both analysis pipelines (RVA, DNP) were used to examine the genome coverage depth with regard to a "normal", diploid reference and document fractional copy number deviations and aneuploidies. The default filtering parameters for CNVs were set as follows: confidence = 0.99 and minimal size = 0.5 Mbp. Additionally, a

custom fractional copy number (CN) < 1.800 for losses and a fractional CN > 2.200 for gains was used.

Bioinformatic evaluation of RNA sequencing data

The RNA sequencing Fastq files were analyzed using the nf-core-rnaseq nextflow pipeline (v.3.14.0) [7]. Briefly, reads were trimmed with Trim Galore [10] and aligned to the human genome GRCh38.p14 using STAR mapper [6] and the Ensembl release v104 for annotation. Quality controls were performed before and after mapping as part of the default execution of the nf-core rnaseq pipeline. The count matrix was generated as final product of the nf-core rnaseq pipeline with Salmon [15]. To compare gene expression between groups of interest, the count matrices were further analyzed with the nf-core differentialabundance nextflow pipeline (v1.4.0) [7] that implements a differential analysis using the DeSeq2 package[12]. Gene expression levels were plotted with ggplot2 [19].

To detect gene fusions, the Arriba [17] (v.2.4.0) workflow was employed on the STAR alignment from the nf-core rnaseq pipeline using the references distributed with Arriba for GRCh38 and the Ensembl v104 gene model (blacklist, known fusions and protein domains). Arriba extracts the sequence of supplementary alignments and discordant mates, followed by a set of filters according to the workflow defaults that reduces the number of false positives. The output was a list of gene fusion candidates and a graphical depiction of each fusion. The second tool used for detection of novel somatic fusion genes was FusionCatcher [14] (v1.33) together with the Human Ensembl v102 annotation release. The resulting high-confidence calls were deduplicated and merged by gene symbol for each of the analyzed datasets. As a final filtering step, we kept only the calls that were reported by both Arriba and FusionCatcher. An identical approach was applied to the two publicly available MB datasets from Azatyan and Zaphiropoulos [1] and Forget *et al.* [8] for independent validation of the detected gene fusion events.

Infinium^{TM} MethylationEPIC v1.0 BeadChip-based DNA methylation profiling

The DNA methylation and copy number analyses using the Infinium^{TM} MethylationEPIC v1.0 BeadChip ("850k EPIC") platform (Illumina, San Diego, CA, USA) were performed at LIFE & BRAIN GmbH (Bonn, Germany). 500 ng of genomic DNA was subjected to bisulfite conversion followed by purification, hybridization to 850k EPICv1 bead chips and scanning. As output of the methylation analysis, a paired red-green (RG) DNA methylation dataset (2 IDAT files) was generated per analyzed sample. Both files were analyzed with the Heidelberg CNS Tumor Methylation Classifier (v12.8) (https://app.epignostix.com) [4, 5], and tumors were correspondingly assigned to their corresponding methylation family and methylation class/subclass.

Comparison of CNVs identified by OGM and DNA methylation microarray-based analyses

Annotation of the DNA methylation IDAT data was converted from hg19 to hg38 using the Python script segment_liftover [9]. Values of the DNA methylation microarray-based segment median (corresponding to log2 median segment intensity normalized to reference) in the interval [-0.1, 0.06] were set to zero. For female samples, the segment median of chromosome Y was also set to zero. If only one CNV was called per chromosome, the boundaries of the segment were extended over the entire chromosome. Optical genome mapping (OGM) values were transformed using log2(fractional copy number) for sex chromosomes of male samples and by log2(fractional copy number / 2) for all other values. For both methods, gaps were filled for each sample by using the function tidygenomics::genome_complement with a log2 fold change of zero. DNA methylation microarray-based segments and combined OGM data were converted to .cns format and CNVKit heatmap [18] with modifications (https://github.com/dkoppstein/cnvkit/tree/plot-conumee) was used to plot summary-level CNVs. Next, CNVMetrics [2] was used to calculate distance metrics between samples. Data frames containing CNV information were pre-processed into CNVMetric format in R [16, 20] and sex chromosomes were omitted [4]. CNV segments were imported into GenomicRanges [11], and calculated pairwise per sample using CNVMetrics::calculateOverlapMetric(., states=c("NEUTRAL", "CNV"), method=c("sorensen")).

Analysis steps were orchestrated with Snakemake [13]. Scripts for data analysis are available upon request.

Supplementary Figures

Figure S1

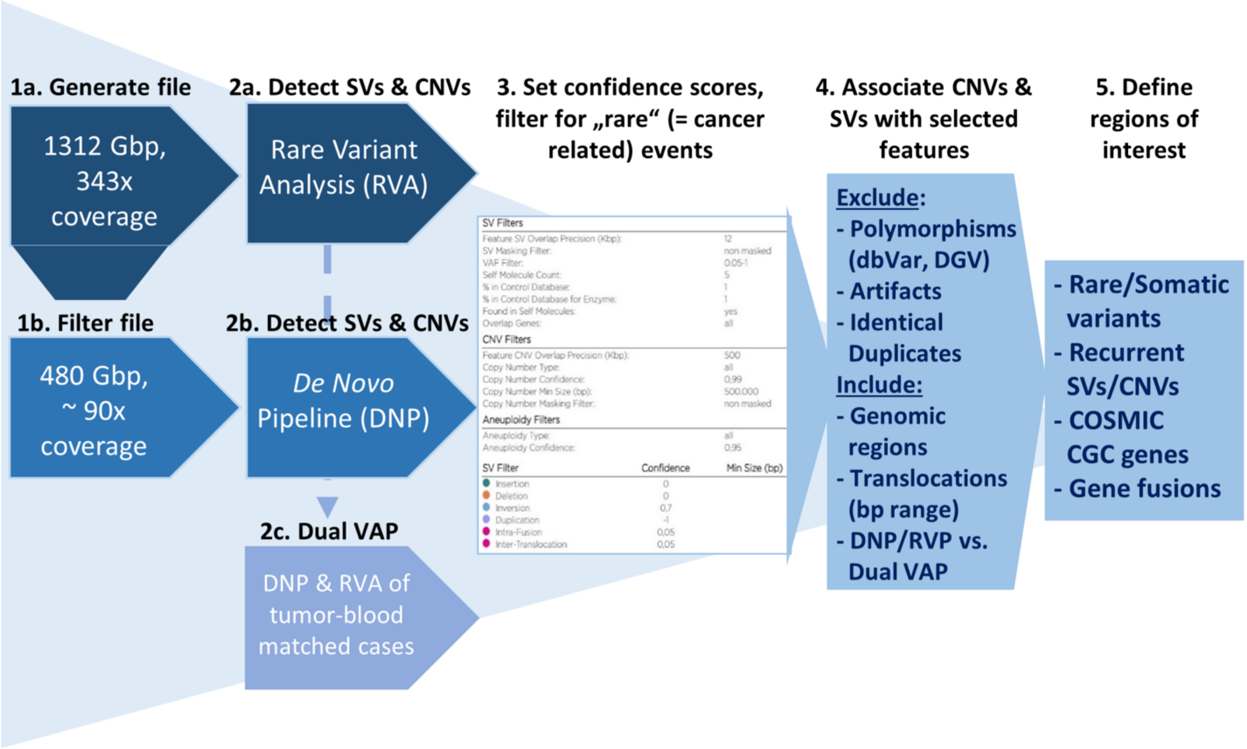

Figure S1. Illustration of the OGM data analysis workflow. Stepwise processing of OGM-generated raw molecule files (1a, b); application of SV-detection and annotation algorithms (2a–c), downstream manual filtering of annotated SVs from different assemblies (3, 4) and final integration into an output with defined regions of interest (5). *bp*, base pairs; *Gbp*, gigabase pairs; *VAP*, Variant Annotation Pipeline by Bionano Genomics; *dbVar*, NCBI's database of genomic structural variation; *DGV*, Database of Genomic Variants; *COSMIC*, Catalogue of Somatic Mutations in Cancer; *CGC*, Cancer Gene Census.

Figure S2

a

Sample	Sorensen metric
MB1t	0.459
MB2t	0.398
MB3t	0.353
MB4t	0.760
MB5t	0.332
MB6t	0.862
MB7t	1.000
MB8t	0.998
MB9t	1.000
MB10t	0.988
MB11t	NA
MB12t	0.010
MB13t	0.996
MB14t	0.970
MB15t	0.834
MB16t	NA
MB17t	0.936
MB18t	0.797
MB19t	0.861
MB20t	0.006
MB21t	0.686
MB22t	0.889
MB23t	0.916
MB24t	0.998
MB25t	0.875
MB26t	0.944
MB27t	0.673
MB28t	0.979
MB29t	0.621
**Mdn**	**0.862**

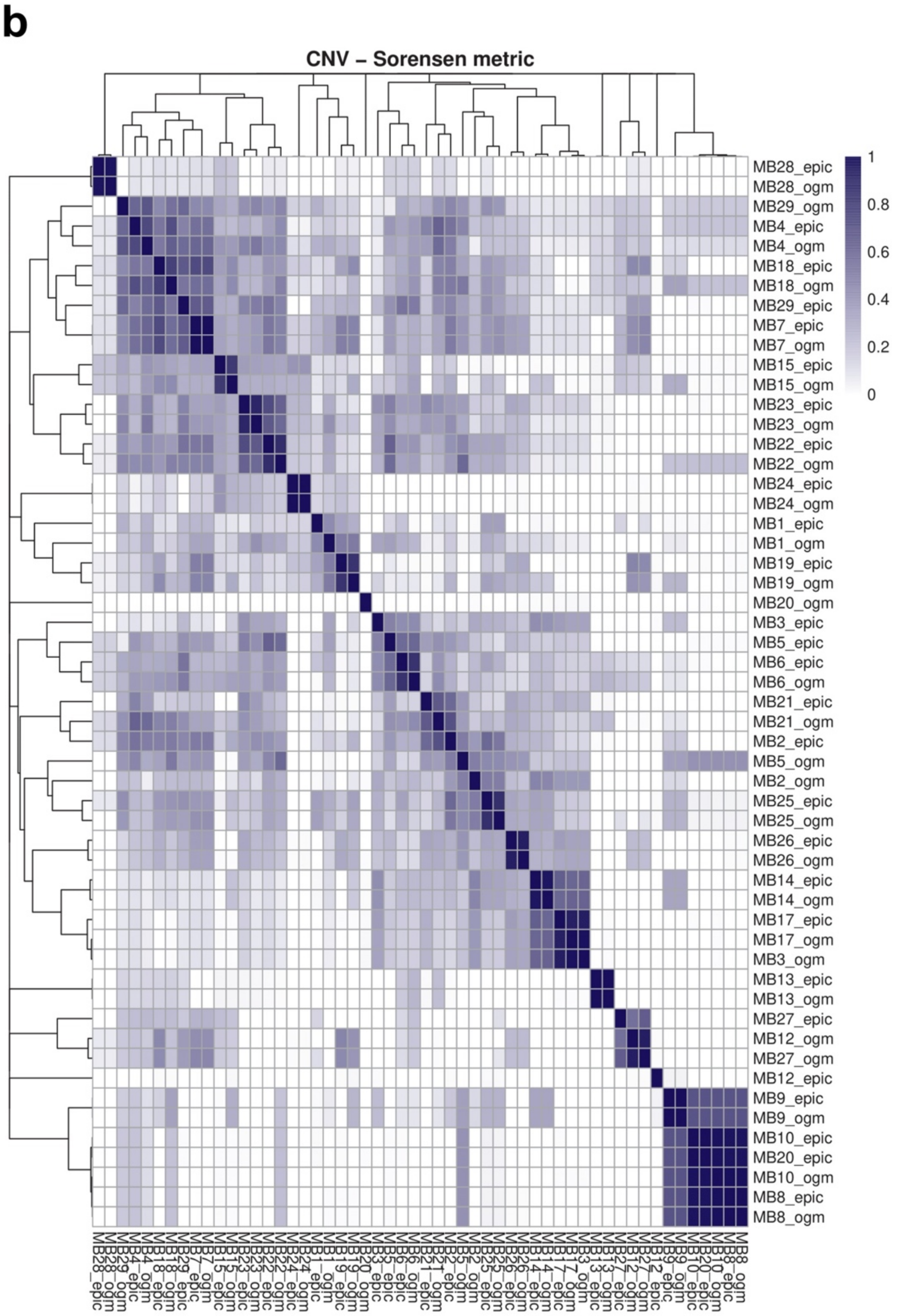

Figure S2. Quantitative comparison of DNA CNV profiles as detected by OGM or DNA methylation microarray-based analysis in 29 MB tumors. (a) Pairwise Sorensen correlation coefficients between CNV profiles detected by OGM vs. DNA methylation microarray-based analyses across all samples and the calculated overall median correlation (Mdn). *NA*, no CN alterations detected. **(b)** Hierarchical clustering of CNV overlap metrics between CNV profiles detected by OGM (_ogm) or DNA methylation microarray (_epic) as calculated by the Sorensen coefficient.

Figure S3

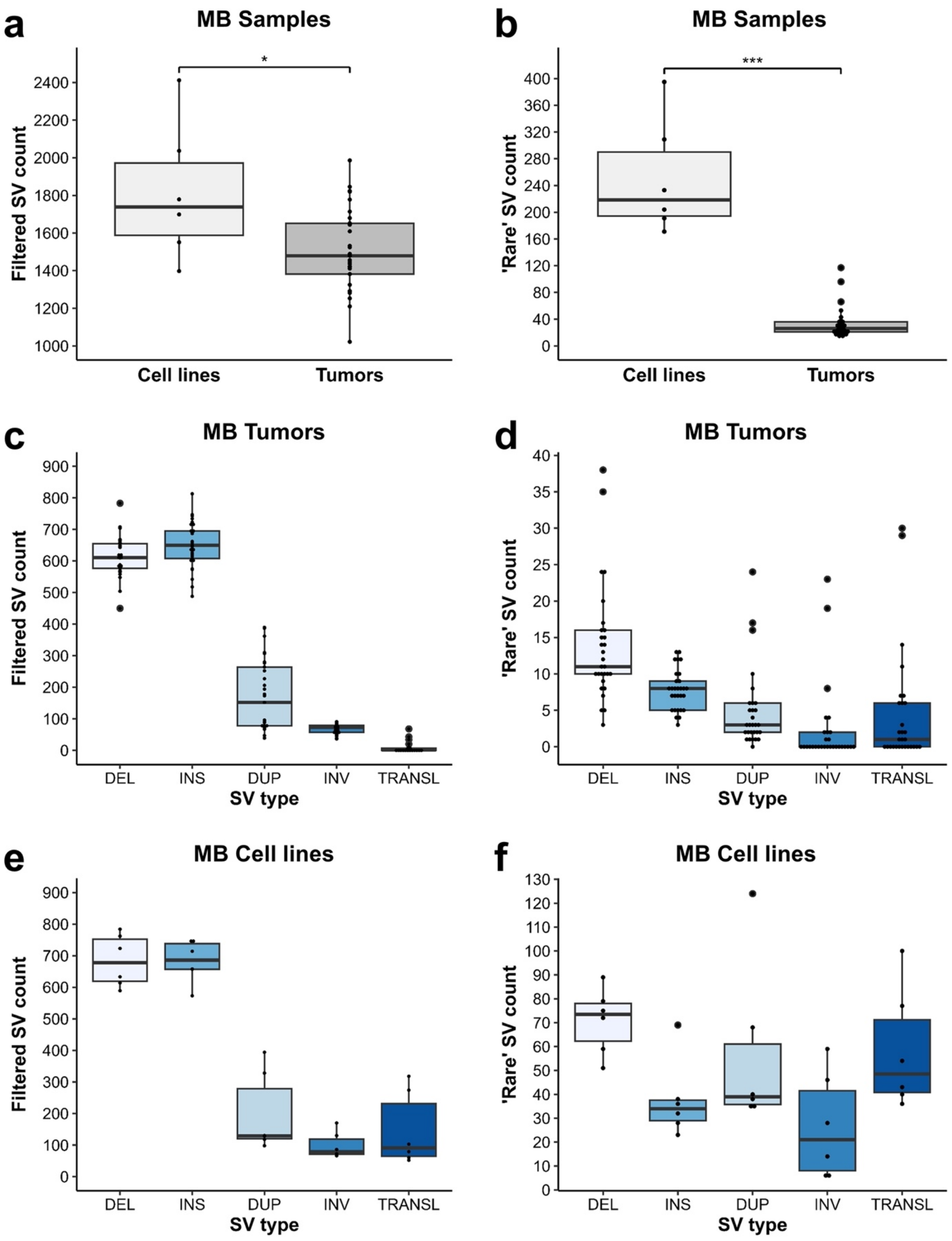

Figure S3. Absolute numbers of SVs detected in MB tumor and cell line samples by OGM stratified according to filtered SVs (left column) and 'rare' SVs (right column). The upper panel represents the total number of SVs detected in MB cell lines (light-grey) vs. MB tumors (dark-grey), dependent on filtering conditions. The middle panel represents the number of specific SV subtypes across MB tumors and the lower panel the number of specific SV subtypes across MB cell lines dependent on filtering conditions. The

graphs on the left show the filtered SVs after application of the general OGM-SV confidence scores while graphs on the right display the number of rare SVs after the exclusion of common variants present in the OGM-integrated control database (right). Sample type comparison of the SV counts was determined by one-way ANOVA: *, $p < 0.05$; ***, $p < 0.001$. The distinct SV types correspond to *DEL*, deletions; *INS*, insertions; *DUP*, duplications; *INV*, inversions; *TRANSL*, intrachromosomal fusions/interchromosomal translocations.

Figure S4

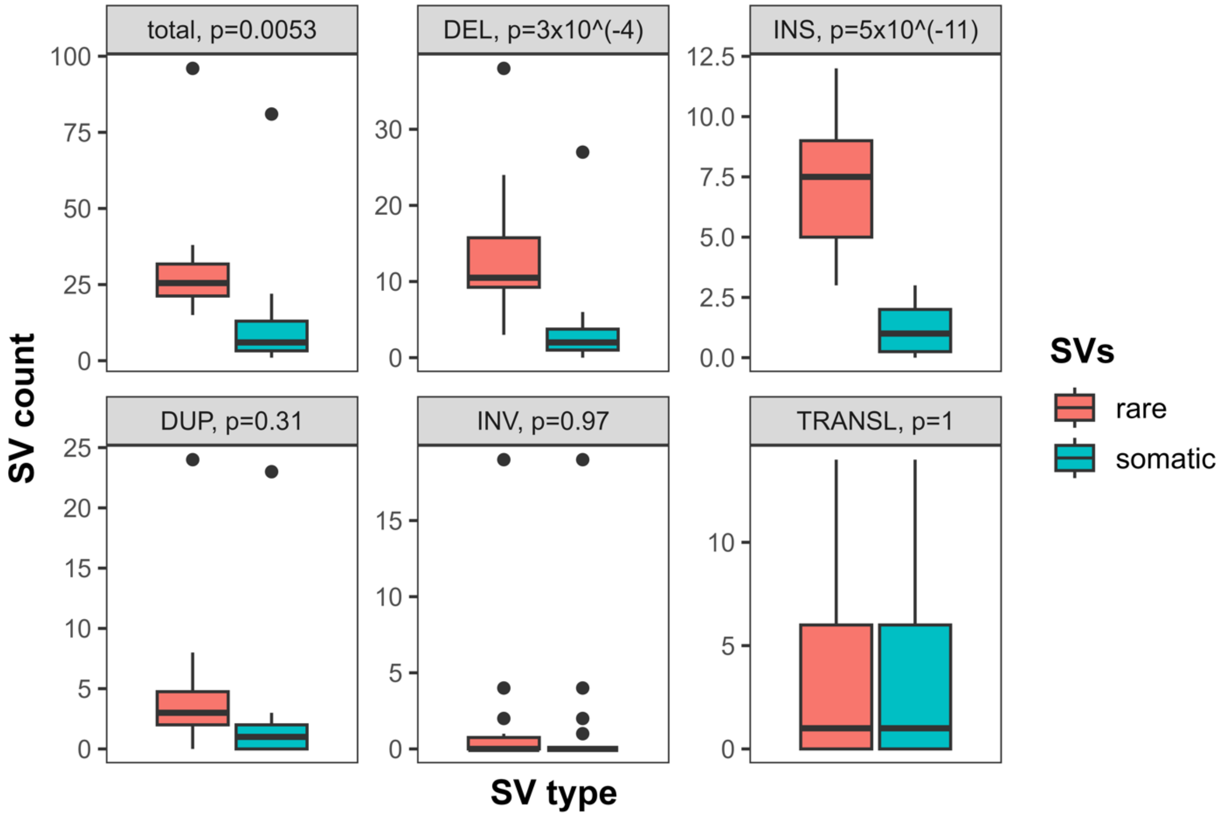

Figure S4. Numbers of high-confidence SVs ≥ 4.5 kbp detect in MB tumor/peripheral blood DNA pairs of 18 patients. Comparison of the absolute SV counts per SV subtype after exclusion of common SVs stratified as rare SVs detected in the MB tumor tissues without subtraction of constitutive SVs also present in the corresponding blood sample (red boxplots), and as somatic SVs detected exclusively in the MB tumor tissues (turquoise boxplots). One-way ANOVA was used to compare SV numbers in the two groups. *kbp*, kilobase pairs; *total*, total SV count; *DEL*, deletions; *INS*, insertions; *DUP*, duplications; *INV*, inversions; *TRANSL*, intrachromosomal fusions/interchromosomal translocations.

Figure S5

a

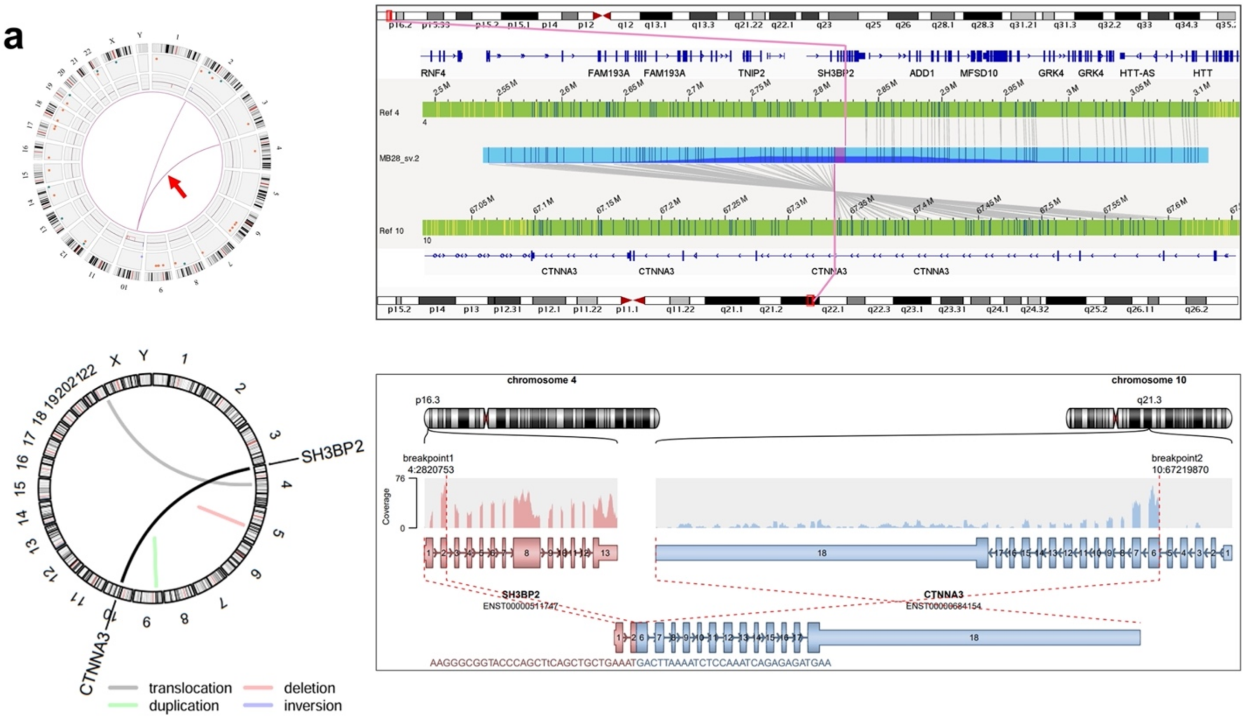
SH3BP2
CTNNA3
translocation
deletion
duplication
inversion
chromosome 4
chromosome 10
breakpoint1
4:2820753
breakpoint2
10:67219870
SH3BP2
ENST00000511747
CTNNA3
ENST00000684154
AAGGGCGGTACCCAGCTCAGCTGCTGAAATGACTTAAAATCTCCAAATCAGAGAGATGAA

b

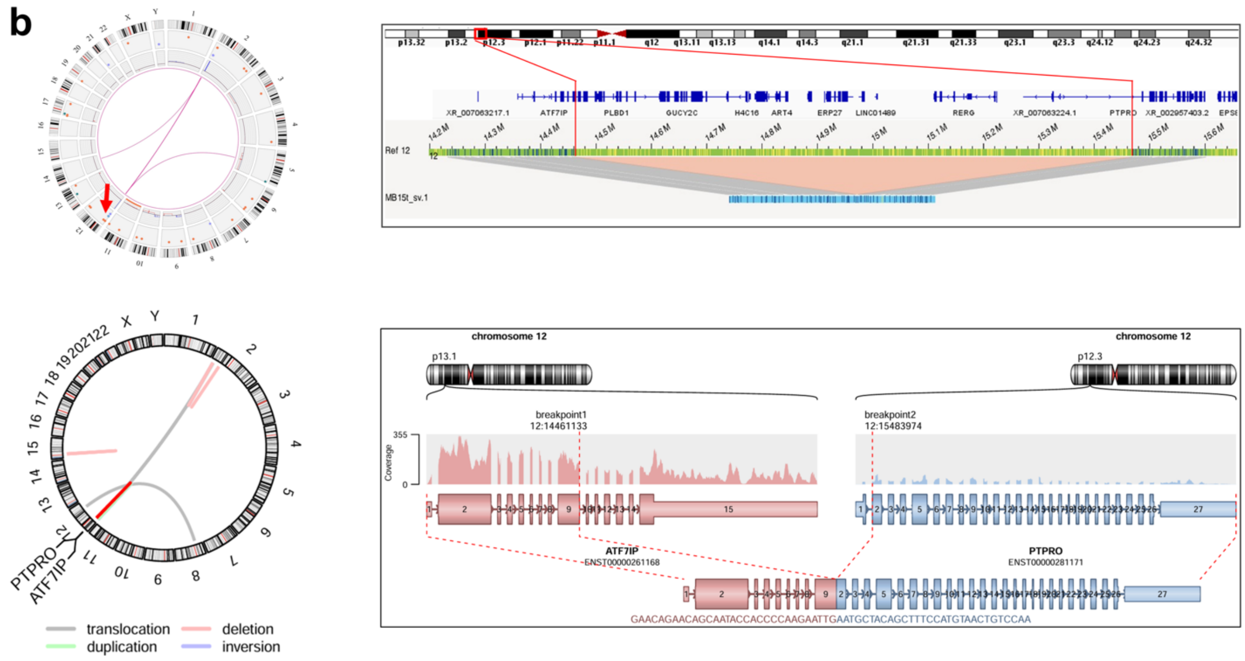
PTPRO
ATF7IP
translocation
deletion
duplication
inversion
chromosome 12
chromosome 12
breakpoint1
12:14461133
breakpoint2
12:15483974
ATF7IP
ENST00000261168
PTPRO
ENST00000281171
GAACAGAACAGCAATACCACCCCAAGAATTGAATGCTACAGCTTTCCATGTAACTGTCCAA

c

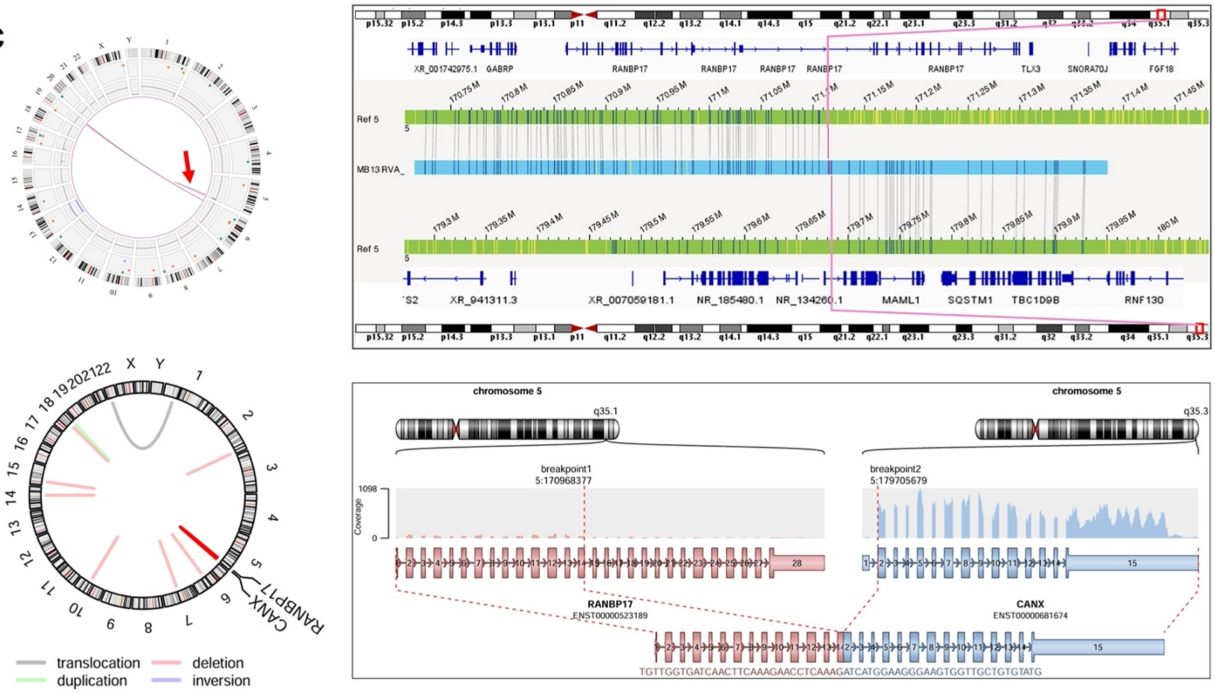
Ref 5
MB13 RVA_
GABRP
RANBP17
TLX3
SNORA70J
FGF18
MAML1
SQSTM1
TBC1D9B
RNF130
RANBP17
CANX
translocation
duplication
deletion
inversion
chromosome 5
q35.1
q35.3
breakpoint1
5:170968377
breakpoint2
5:179705679
Coverage
1098
0
RANBP17
ENST00000523189
CANX
ENST00000681674
TGTTGGTGATCAACTTCAAAGAACCTCAAAGATCATGGAAGGGAAGTGGTTGCTGTGTATG

d

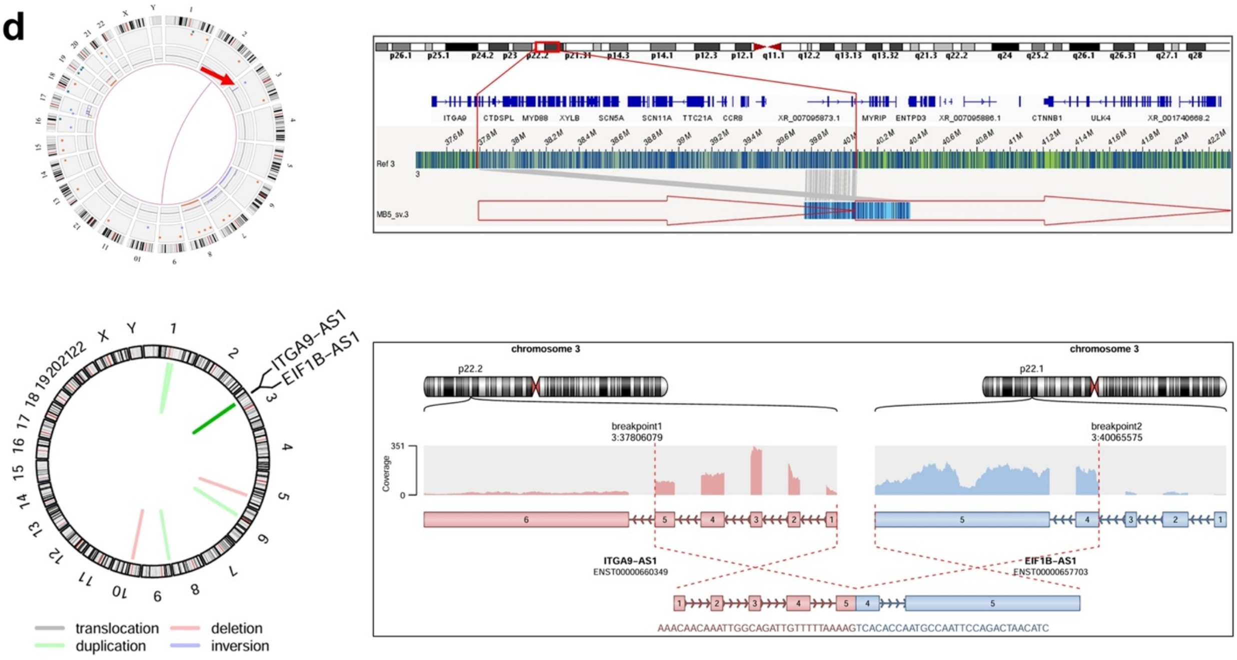
ITGA9
CTDSPL
MYD88
XYLB
SCN5A
SCN11A
TTC21A
CCR8
MYRIP
ENTPD3
CTNNB1
ULK4
Ref 3
MB5_sv.3
ITGA9-AS1
EIF1B-AS1
translocation
duplication
deletion
inversion
chromosome 3
p22.2
p22.1
breakpoint1
3:37806079
breakpoint2
3:40065575
Coverage
351
0
ITGA9-AS1
ENST00000660349
EIF1B-AS1
ENST00000657703
AAACAACAAATTGGCAGATTGTTTTTAAAAGTCACACCAATGCCAATTCCAGACTAACATC

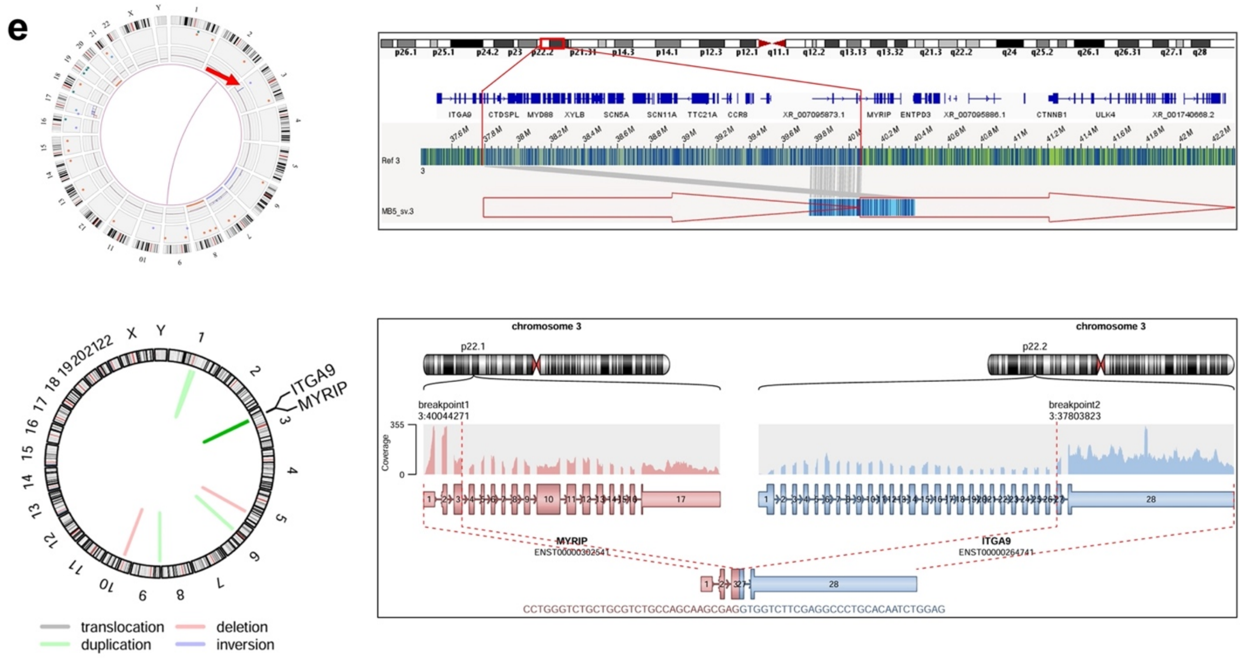

Figure S5. Depiction of cancer-related gene fusions detected by OGM in MB tumors in addition to the *STAG2::ARHGAP36* fusion in MB20t depicted in Fig. 6. For each tumor, two representations of the same gene fusion events are provided, with the upper circos plots (left) and SV-views (right) being derived from OGM analysis and the lower circos plots (left) and SV-views (right) being derived from Arriba analysis of the RNA sequencing data. The circos plots emphasize all rare SVs detected in the respective MB tumor sample, with the translocation/fusion event of interest indicated by red arrows, and the respective SV-views zoom into the specific translocation/fusion event. In the OGM-SV-view, the reference regions are indicated in green with the respective whole chromosome above and below. The light blue OGM consensus map (map incorporating the SV) is shown in the middle and illustrates the partial alignment to the reference regions. In the Arriba-SV-view, a schematic visualization of detected transcripts of the fusion partners, their coverage, orientation and the retained exons contributing to the fusion transcript are visualized.

(a) In MB28t (SHH–infant), OGM detected a partially inverted, interchromosomal translocation between the p-arm of chr4 and the q-arm of chr10, t(4;10)(p16.3;q21.3), overlapping with the *SH3BP2* gene and a large, intronic region of the *CTNNA3* gene. The *SH3BP2::CTNNA3* gene fusion predicted by OGM was confirmed by ENST00000511747.6(SH3BP2):e.1_2::ENST00000684154.1(CTNNA3):e.6_18 fusion transcripts detected by RNA sequencing.

(b) In MB15t (SHH–child), OGM revealed a large deletion affecting a highly rearranged region on the p-arm of chr12. The deletion breakpoints mapped within the genes *ATF7IP* and *PTPRO*. This *ATF7IP::PTPRO* gene fusion is supported by RNA sequencing demonstrating ENST00000261168.9(ATF7IP):e.1_9::ENST00000281171.9(PTPRO):e.2_27 fusion transcripts.

(c) In MB13t (SHH–child), OGM detected a deletion-co-localized, intra-chromosomal translocation affecting the q-arm of chr5. The breakpoints were within the genes *RANBP17* and *CANX* and OGM identified a *RANBP17::CANX* putative gene fusion. This gene fusion is supported by RNA sequencing demonstrating ENST00000523189.6(RANBP17):e.1_14::ENST00000681674.1(CANX):e.1_15 fusion transcripts.

(d, e) OGM identified a duplicated region on the p-arm of chr3 in MB5t (Group 4). The duplication breakpoints mapped within genomic regions overlapping the genes *EIF1B-AS1* (d) and *MYRIP* (e), as well as *ITGA9-AS1* (d) and *ITGA9* (e). Thus, OGM revealed the two duplication-dependent putative gene fusions, which were both supported by detection of the respective ENST00000660349.1(ITGA9-AS1):e.1_5::ENST00000657703.1(EIF1B-AS1):e.4_5 (d) and ENST00000302541.11(MYRIP):e.1_3::ENST00000264741.10(ITGA9):e.27_28 (e) fusion transcripts upon RNA sequencing.

Figure S6

a

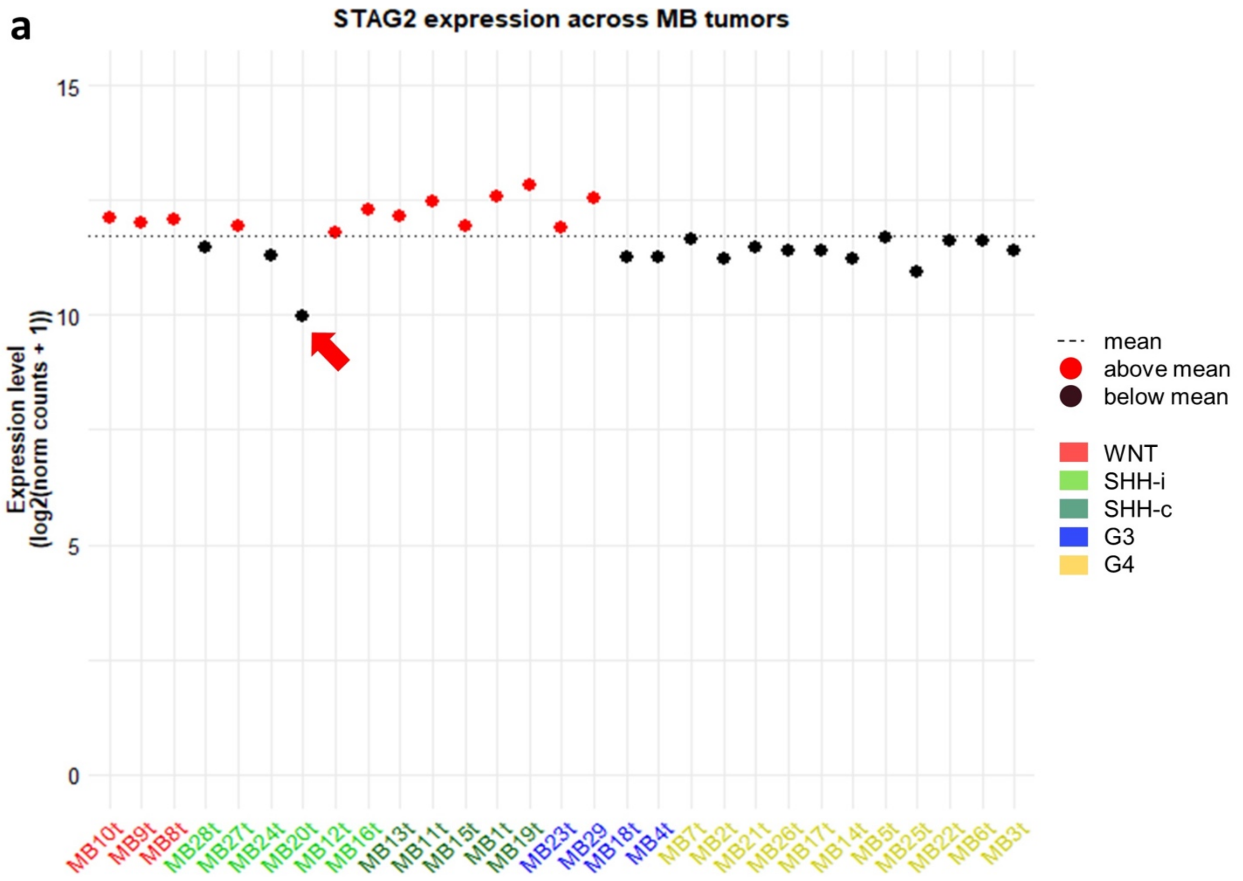

STAG2 expression across MB tumors
Expression level
(log2(norm counts + 1))
15
10
5
0
MB10t
MB9t
MB8t
MB28t
MB27t
MB24t
MB20t
MB12t
MB16t
MB13t
MB11t
MB15t
MB1t
MB19t
MB23t
MB29
MB18t
MB4t
MB7t
MB2t
MB21t
MB26t
MB17t
MB14t
MB5t
MB25t
MB22t
MB6t
MB3t
mean
above mean
below mean
WNT
SHH-i
SHH-c
G3
G4

b

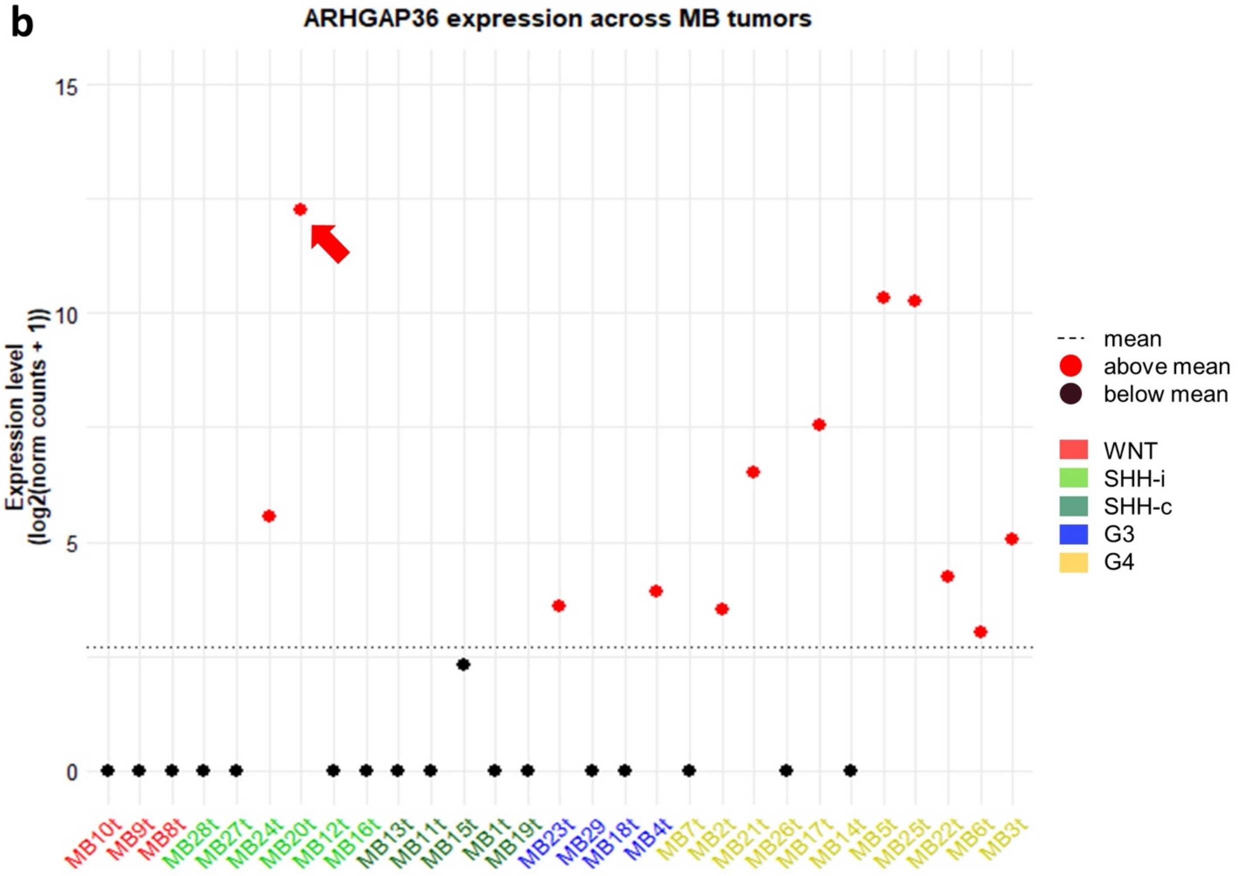

ARHGAP36 expression across MB tumors
Expression level
(log2(norm counts + 1))
15
10
5
0
MB10t
MB9t
MB8t
MB28t
MB27t
MB24t
MB20t
MB12t
MB16t
MB13t
MB11t
MB15t
MB1t
MB19t
MB23t
MB29
MB18t
MB4t
MB7t
MB2t
MB21t
MB26t
MB17t
MB14t
MB5t
MB25t
MB22t
MB6t
MB3t
mean
above mean
below mean
WNT
SHH-i
SHH-c
G3
G4

c

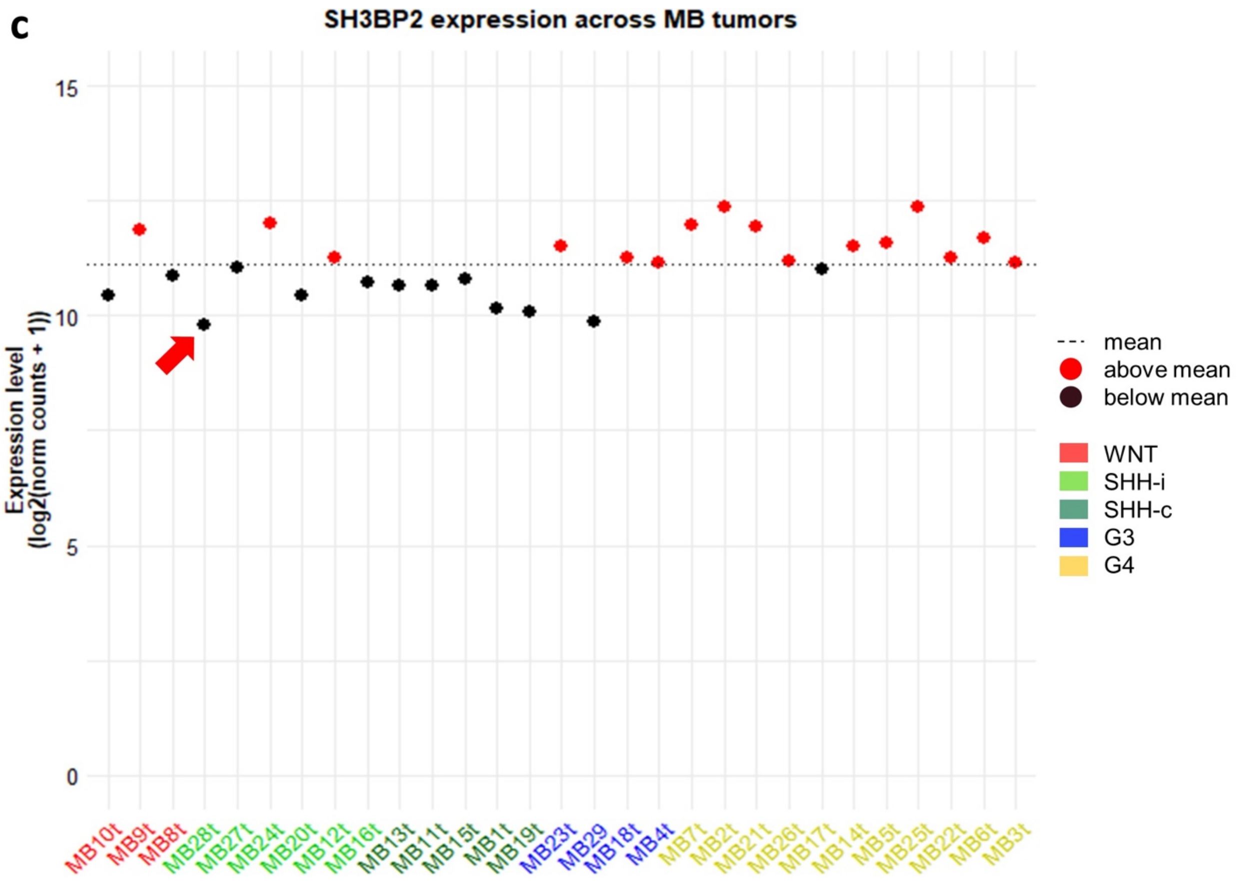
SH3BP2 expression across MB tumors
Expression level
(log2(norm counts + 1))
15
10
5
0
MB10t
MB9t
MB8t
MB28t
MB27t
MB24t
MB20t
MB12t
MB16t
MB13t
MB11t
MB15t
MB1t
MB19t
MB23t
MB29
MB18t
MB4t
MB7t
MB2t
MB21t
MB26t
MB17t
MB14t
MB5t
MB25t
MB22t
MB6t
MB3t
mean
above mean
below mean
WNT
SHH-i
SHH-c
G3
G4

d

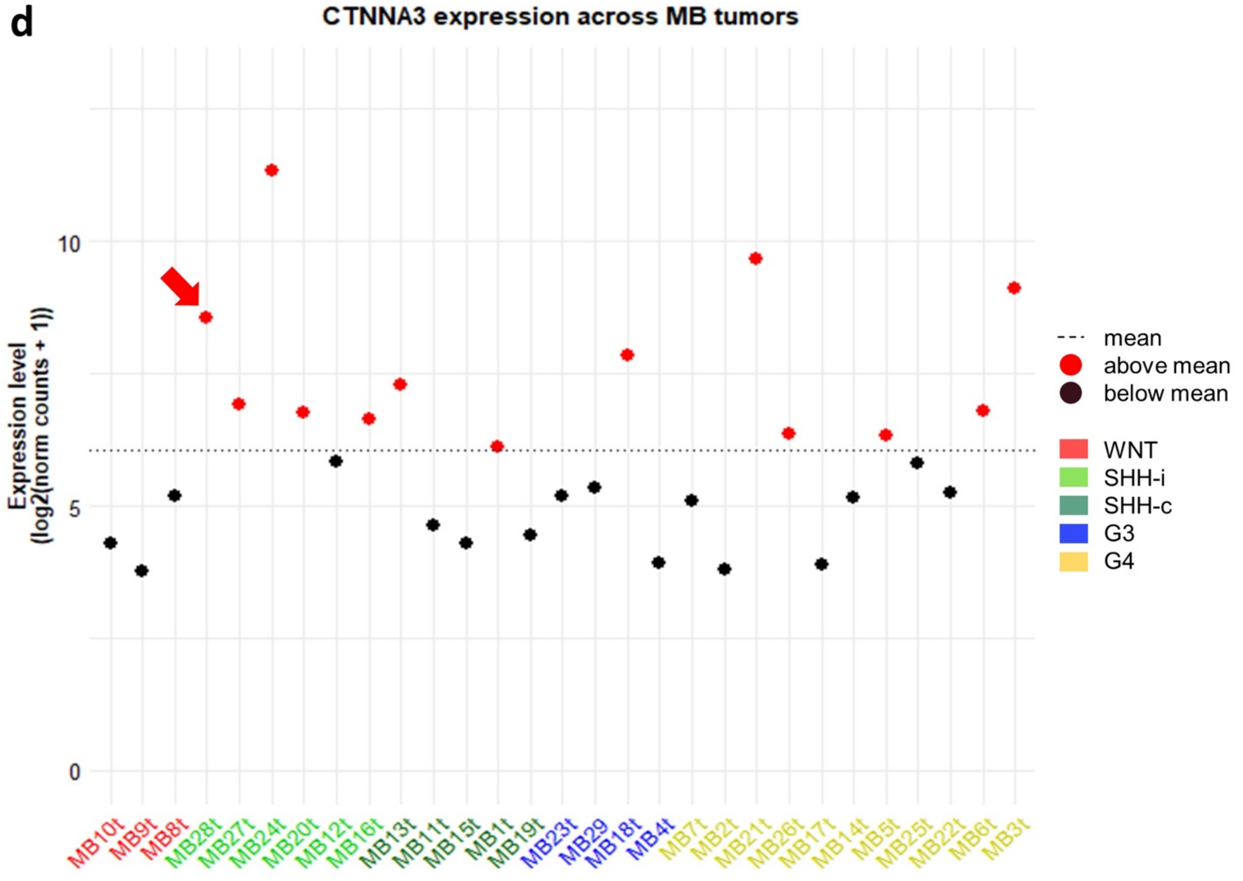
CTNNA3 expression across MB tumors
Expression level
(log2(norm counts + 1))
10
5
0
MB10t
MB9t
MB8t
MB28t
MB27t
MB24t
MB20t
MB12t
MB16t
MB13t
MB11t
MB15t
MB1t
MB19t
MB23t
MB29
MB18t
MB4t
MB7t
MB2t
MB21t
MB26t
MB17t
MB14t
MB5t
MB25t
MB22t
MB6t
MB3t
mean
above mean
below mean
WNT
SHH-i
SHH-c
G3
G4

e

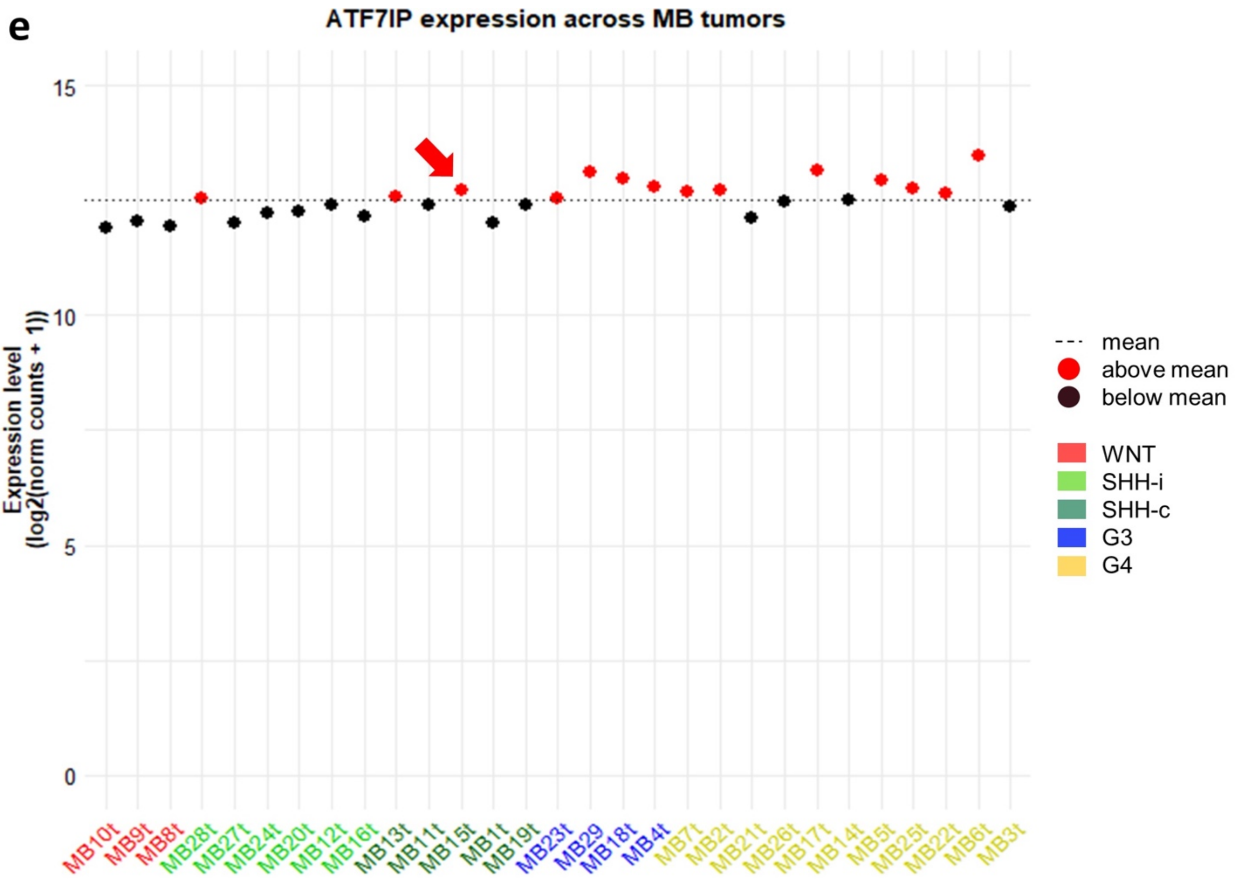
ATF7IP expression across MB tumors
Expression level
(log2(norm counts + 1))
15
10
5
0
MB10t
MB9t
MB8t
MB28t
MB27t
MB24t
MB20t
MB12t
MB16t
MB13t
MB11t
MB15t
MB1t
MB19t
MB23t
MB29
MB18t
MB4t
MB7t
MB2t
MB21t
MB26t
MB17t
MB14t
MB5t
MB25t
MB22t
MB6t
MB3t
mean
above mean
below mean
WNT
SHH-i
SHH-c
G3
G4

f

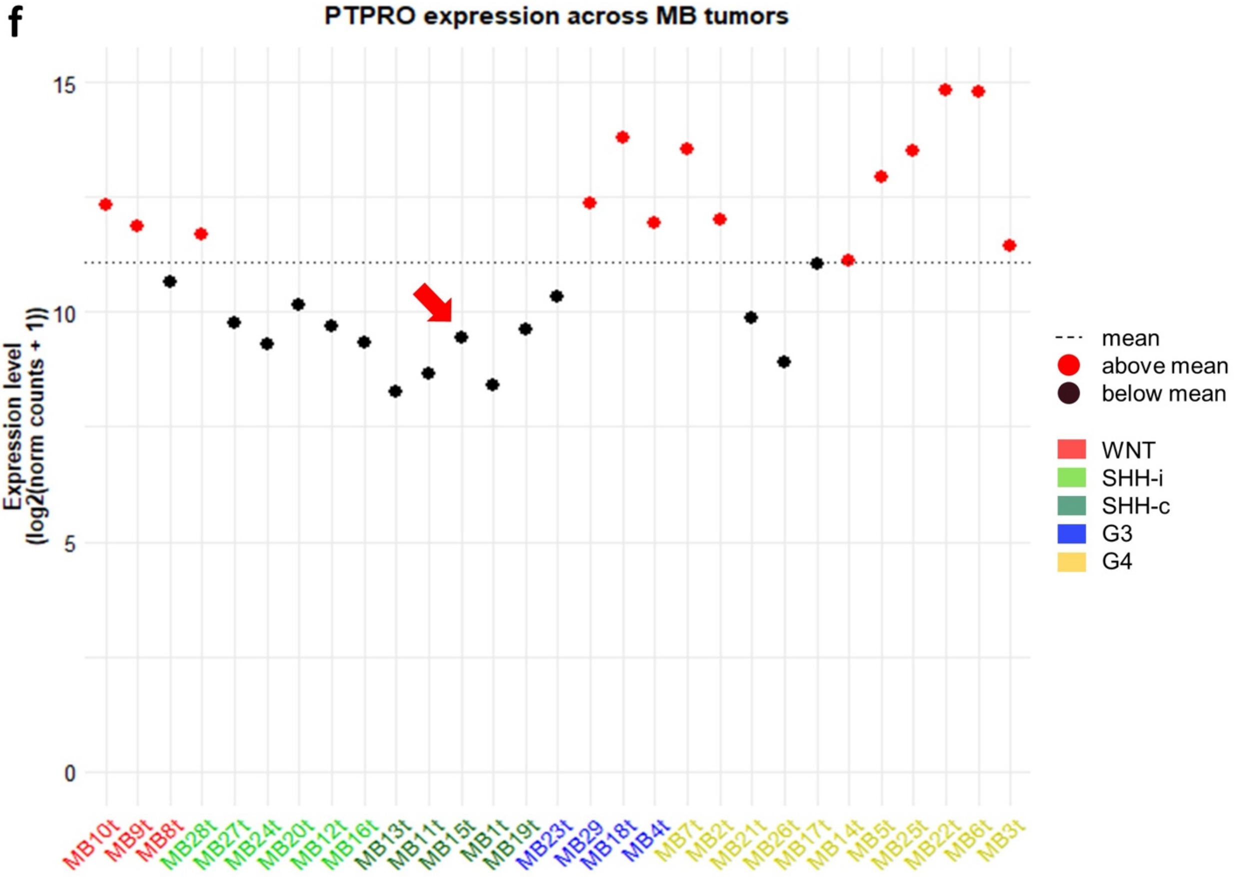
PTPRO expression across MB tumors
Expression level
(log2(norm counts + 1))
15
10
5
0
MB10t
MB9t
MB8t
MB28t
MB27t
MB24t
MB20t
MB12t
MB16t
MB13t
MB11t
MB15t
MB1t
MB19t
MB23t
MB29
MB18t
MB4t
MB7t
MB2t
MB21t
MB26t
MB17t
MB14t
MB5t
MB25t
MB22t
MB6t
MB3t
mean
above mean
below mean
WNT
SHH-i
SHH-c
G3
G4

g

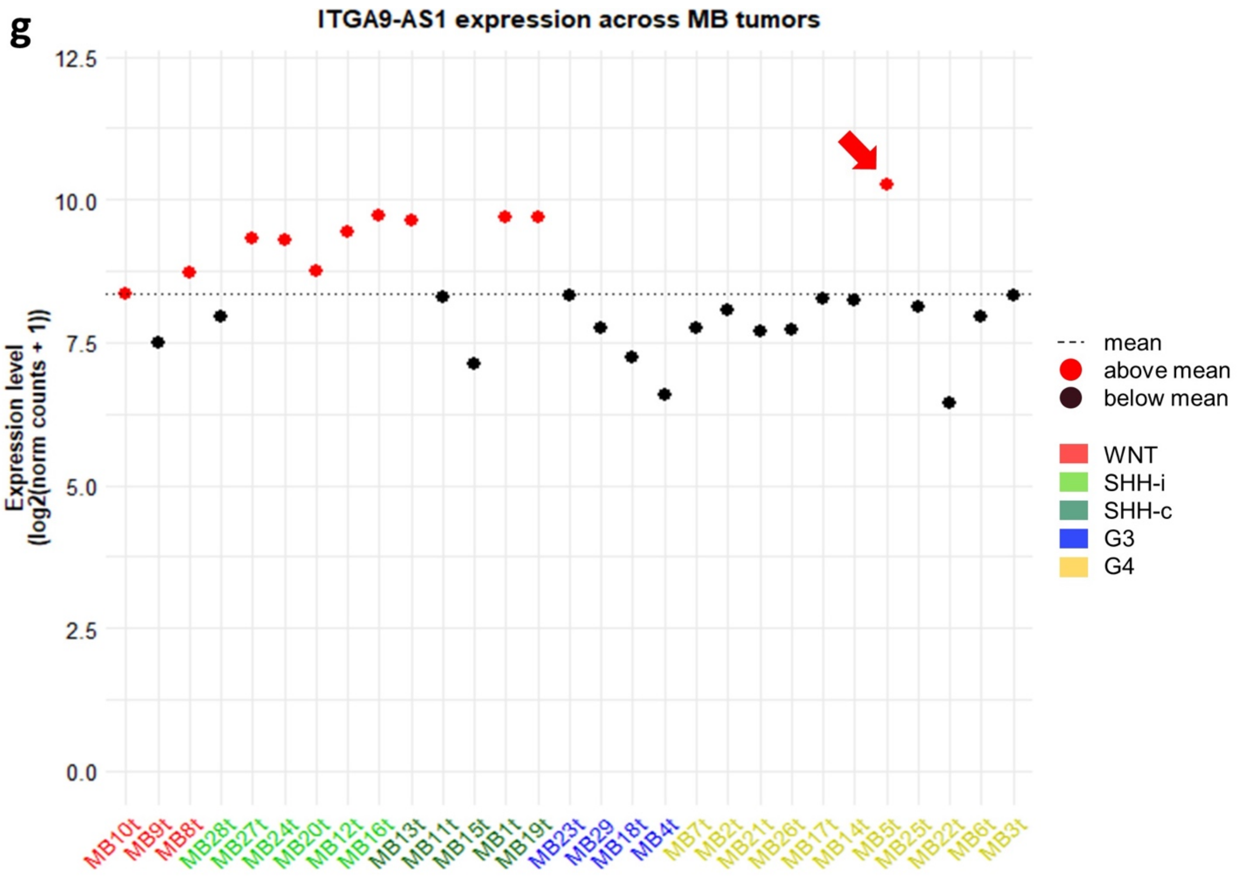

ITGA9-AS1 expression across MB tumors
Expression level
(log2(norm counts + 1))
12.5
10.0
7.5
5.0
2.5
0.0
MB10t
MB9t
MB8t
MB28t
MB27t
MB24t
MB20t
MB12t
MB16t
MB13t
MB11t
MB15t
MB1t
MB19t
MB23t
MB29
MB18t
MB4t
MB7t
MB2t
MB21t
MB26t
MB17t
MB14t
MB5t
MB25t
MB22t
MB6t
MB3t
mean
above mean
below mean
WNT
SHH-i
SHH-c
G3
G4

h

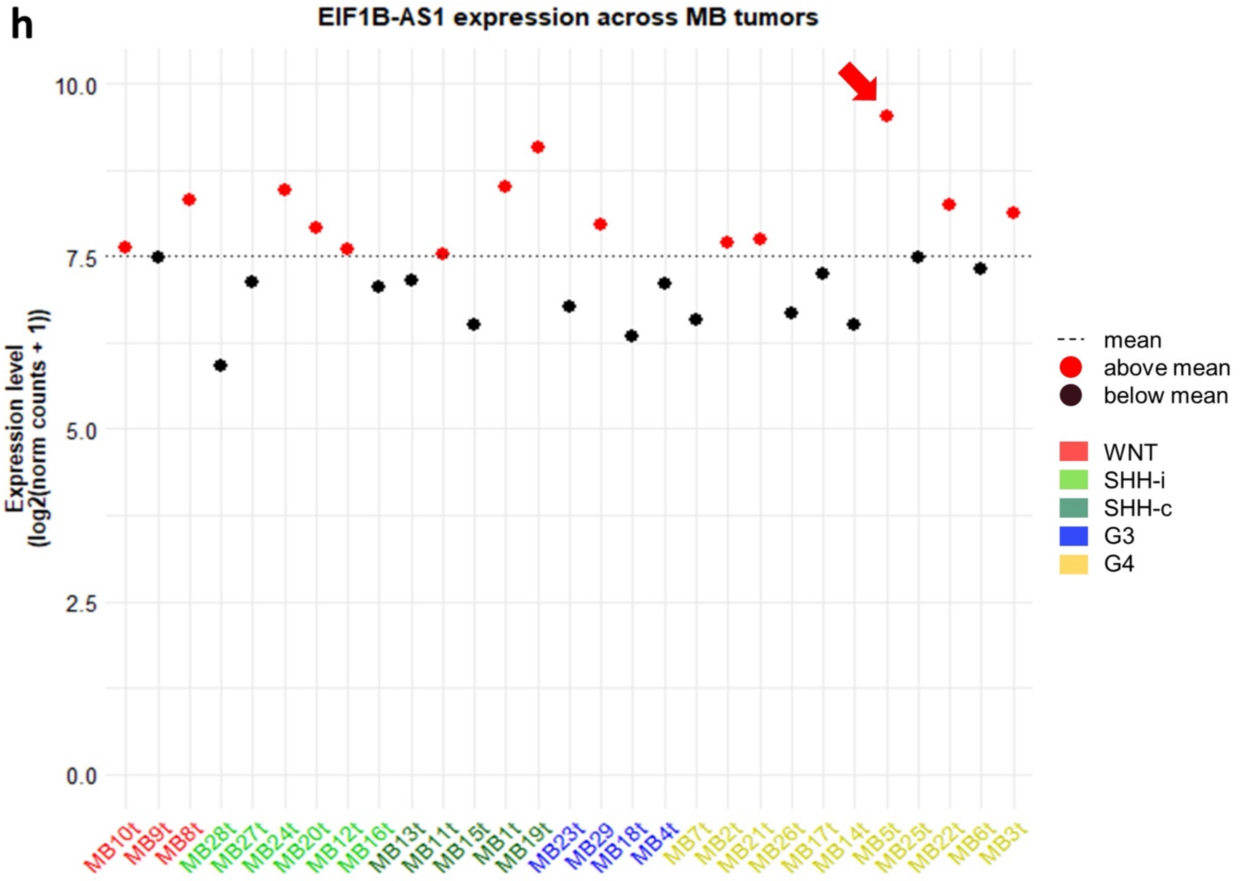

EIF1B-AS1 expression across MB tumors
Expression level
(log2(norm counts + 1))
10.0
7.5
5.0
2.5
0.0
MB10t
MB9t
MB8t
MB28t
MB27t
MB24t
MB20t
MB12t
MB16t
MB13t
MB11t
MB15t
MB1t
MB19t
MB23t
MB29
MB18t
MB4t
MB7t
MB2t
MB21t
MB26t
MB17t
MB14t
MB5t
MB25t
MB22t
MB6t
MB3t
mean
above mean
below mean
WNT
SHH-i
SHH-c
G3
G4

i

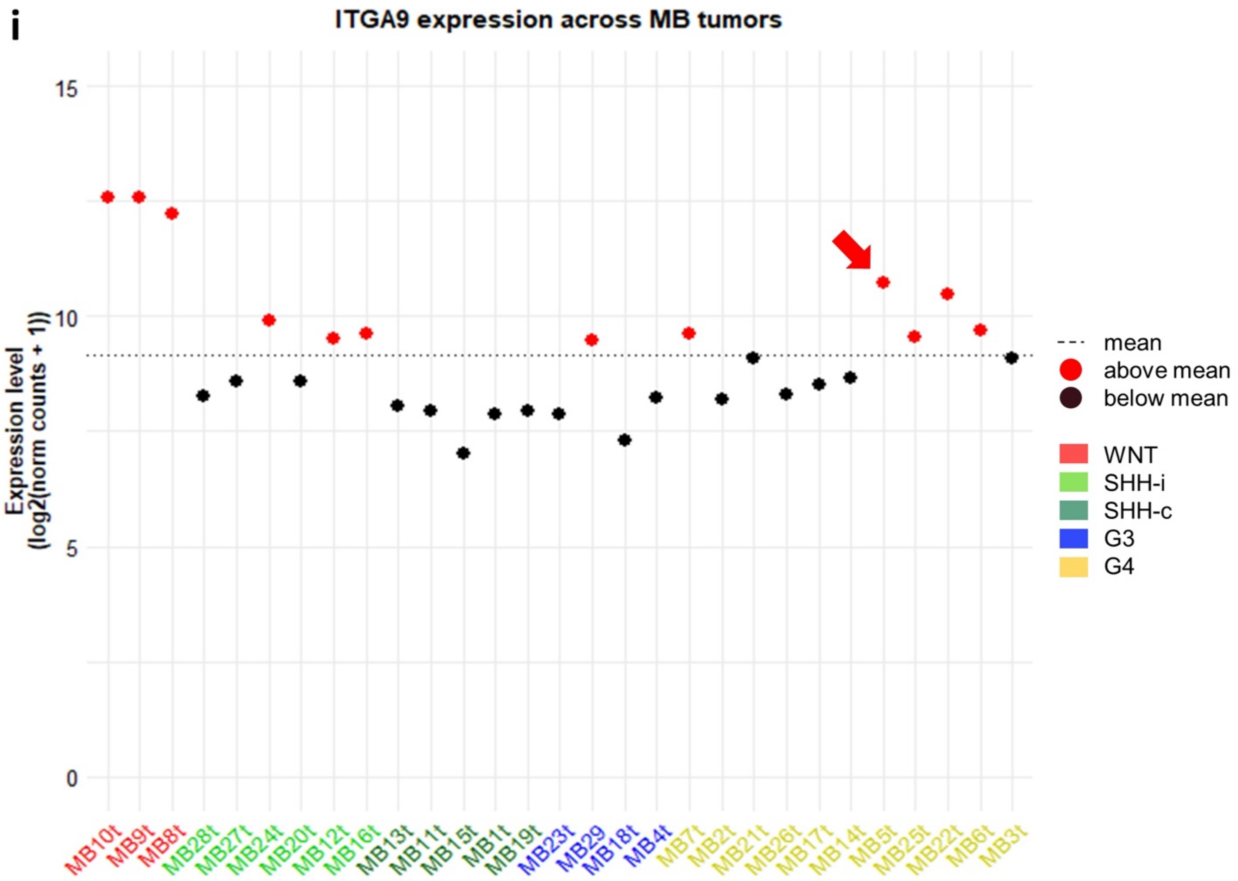

j

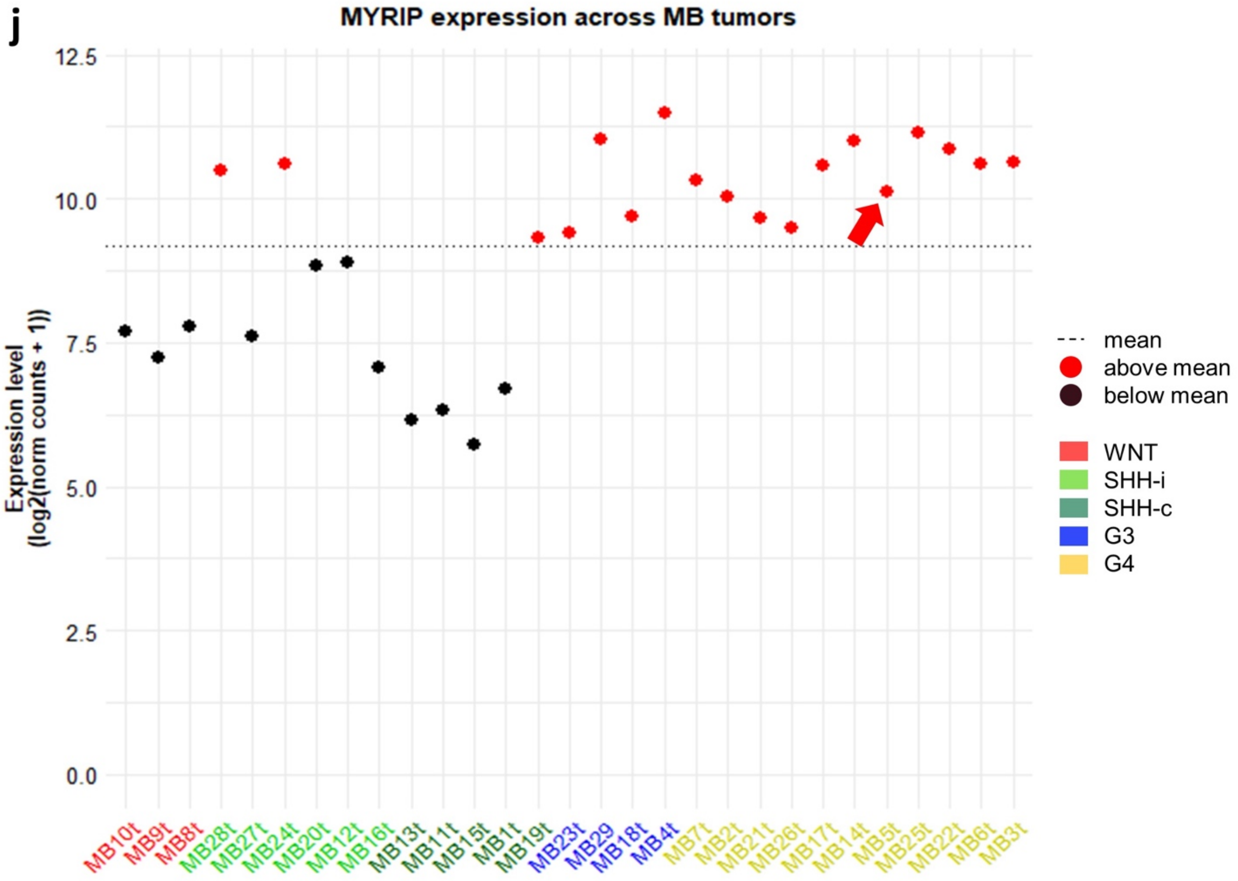

(Figure legend on the next page)

Figure S6. Expression levels of genes implicated in the MB-associated gene fusions depicted in Fig. 6 and Fig. S5 across all MB cases. The expression levels in log2(normalized counts +1) are plotted vertically against the individual MB samples (horizontally), which are group-wise separated by distinct colours from left to right: *red*, MB WNT; *light-green*, MB SHH–infant; *dark-green*, MB SHH–child; *blue*, MB Group 3; *yellow*, MB Group 4. The red arrows indicate the gene expression levels of the specific cases carrying the respective gene fusions. The gene fusion partners are depicted together on each pages: **(a, b)** *STAG2* and *ARHGAP36* in MB20t, **(c, d)** *SH3BP2* and *CTNNA3* in MB28t, **(e, f)** *ATF7IP* and *PTPRO* in MB15t, **(g, h)** *EIF1B-AS1* and *ITGA9-AS1* as well as **(i, j)** *MYRIP* and *ITGA9* in MB5t.

Figure S7

MB1t: MB, SHH-activated, subclass 4

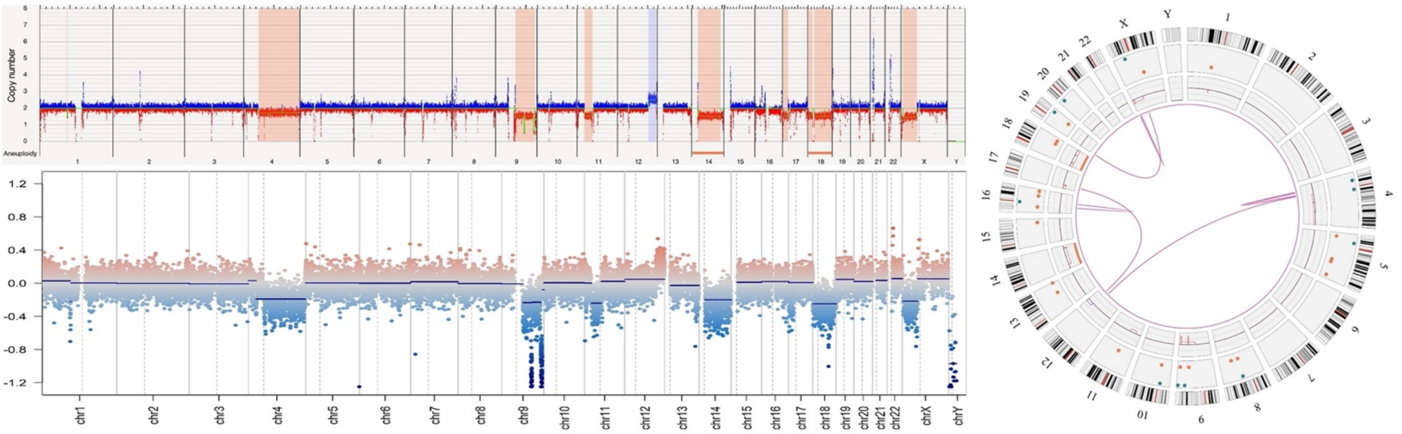

MB2t: MB, non-WNT/non-SHH, Group 4 subtype, subclass V

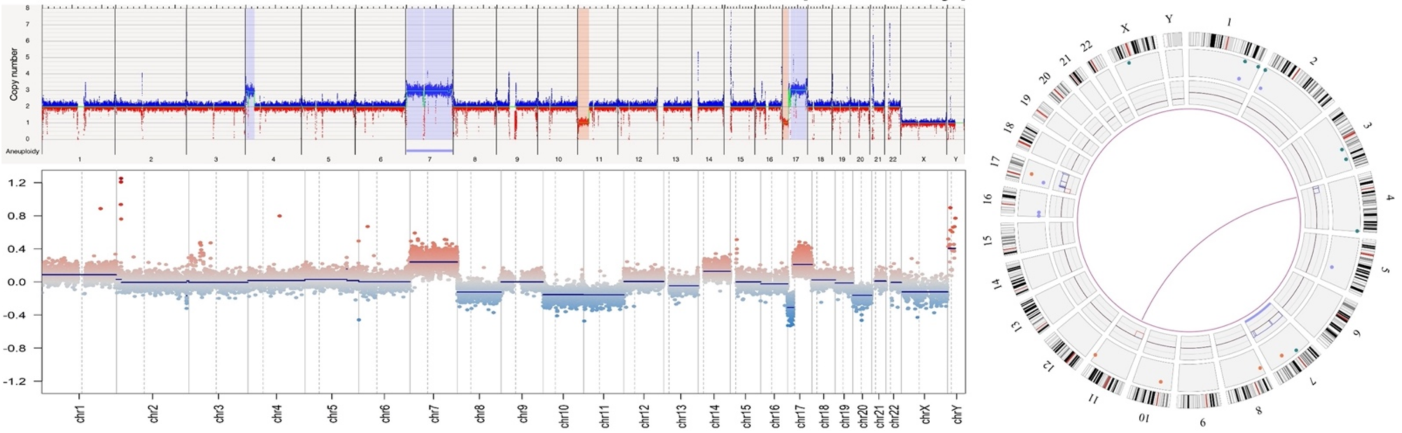

MB3t: MB, non-WNT/non-SHH, Group 4 subtype, subclass VIII

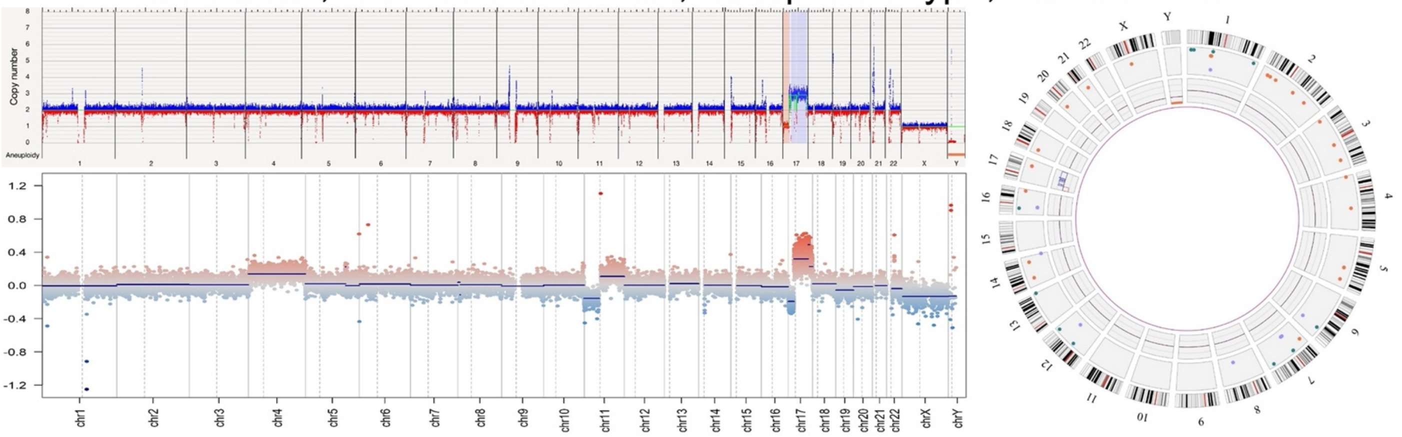

MB4t: MB, non-WNT/non-SHH, Group 3 subtype

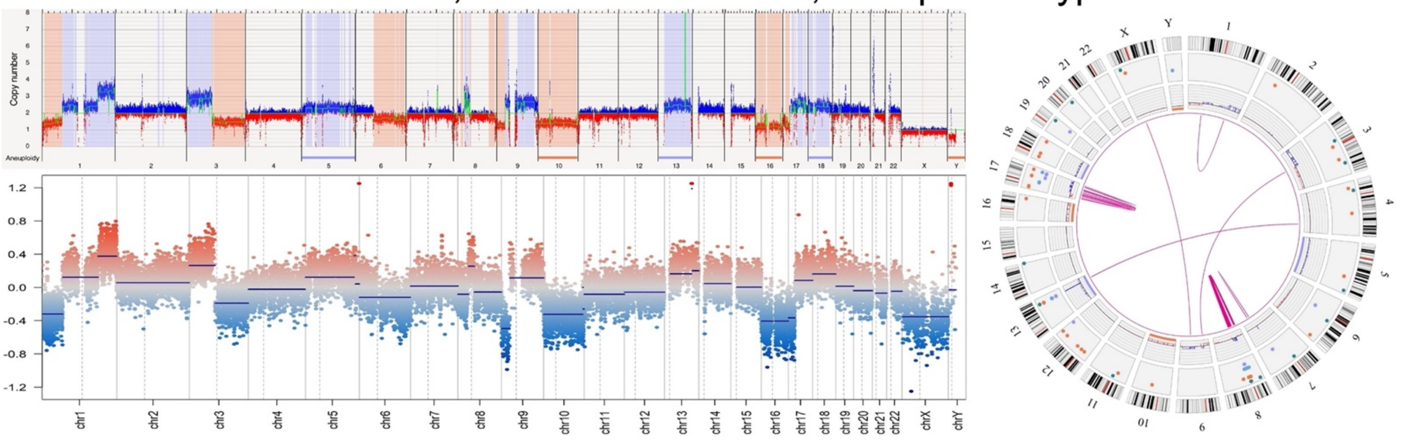

MB5t: MB, non-WNT/non-SHH, Group 4 subtype

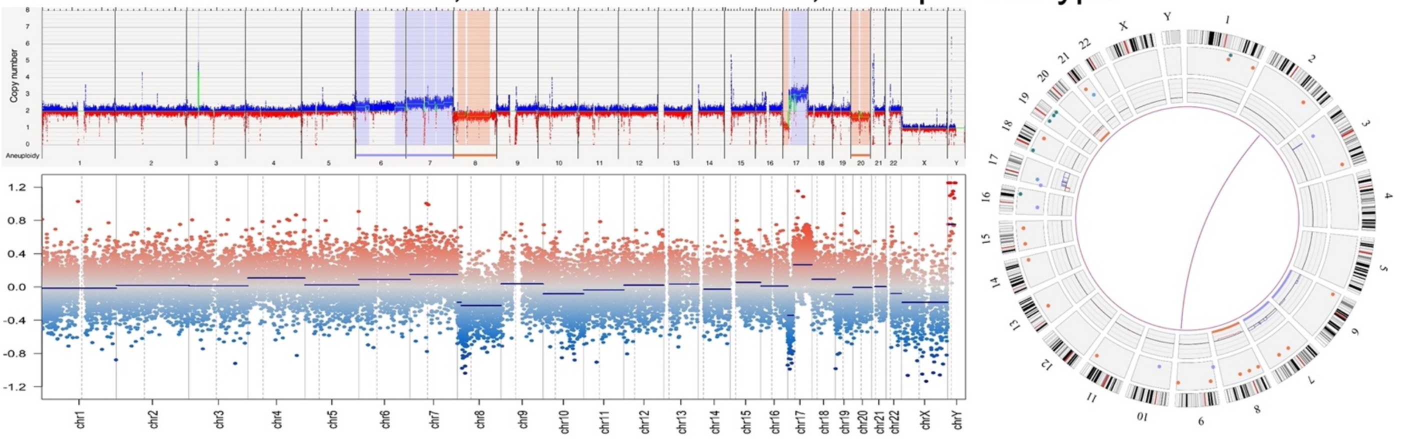

MB6t: MB, non-WNT/non-SHH, Group 4 subtype, subclass V

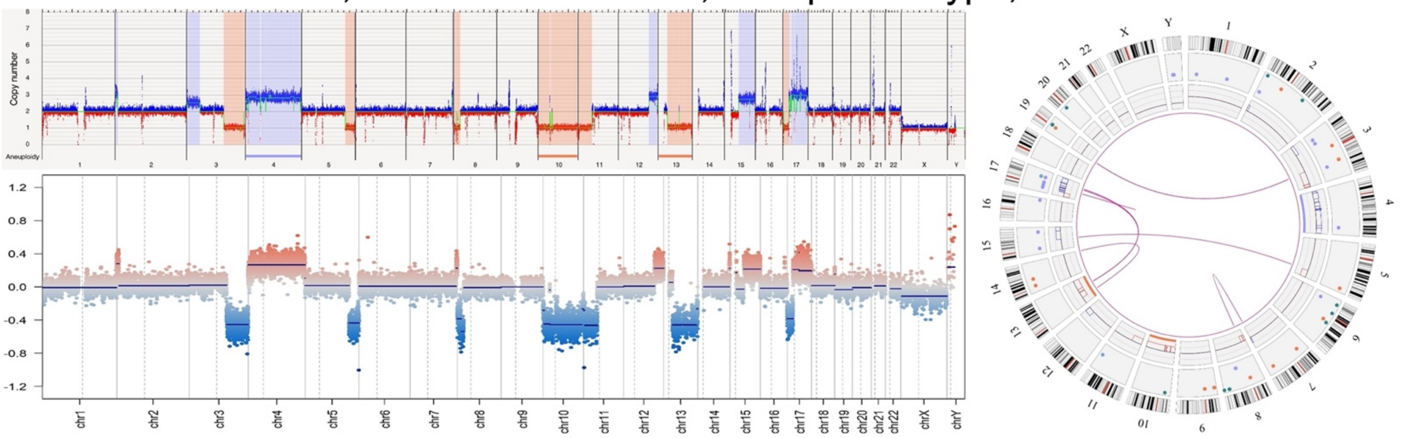

MB7t: MB, non-WNT/non-SHH, Group 4 subtype, subclass VII

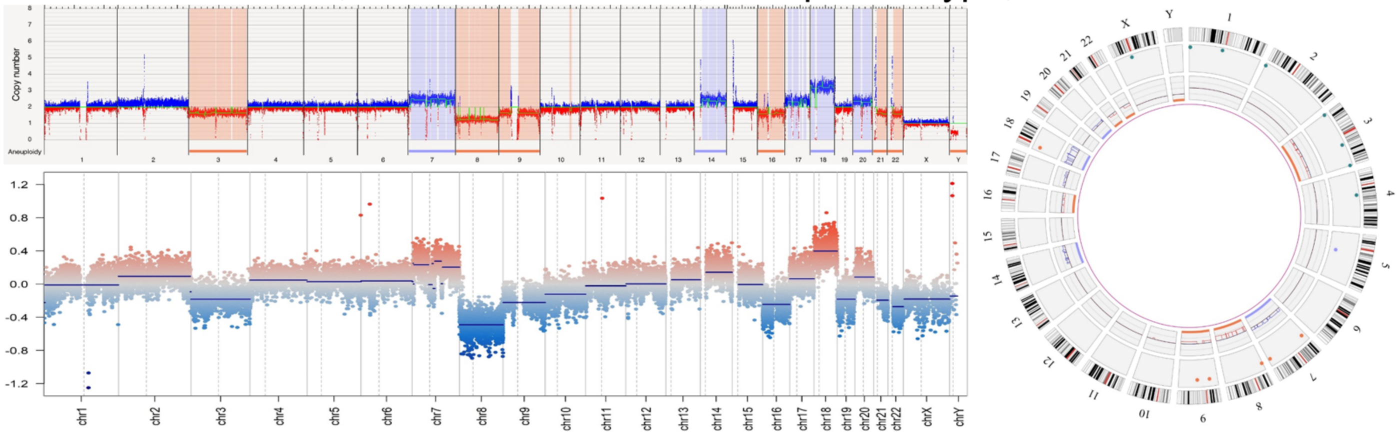

MB8t: MB, WNT-activated

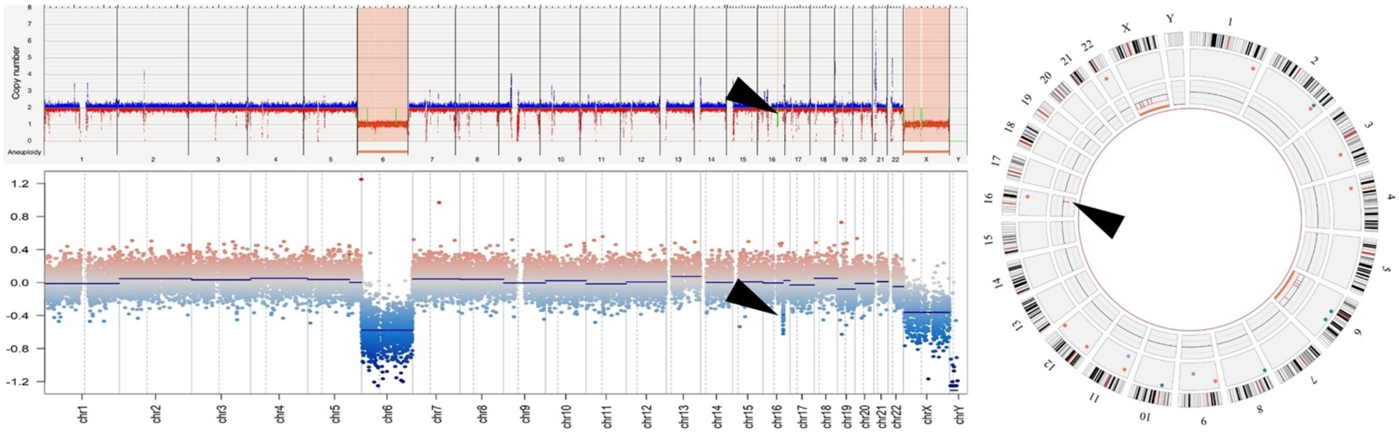

MB9t: MB, WNT-activated

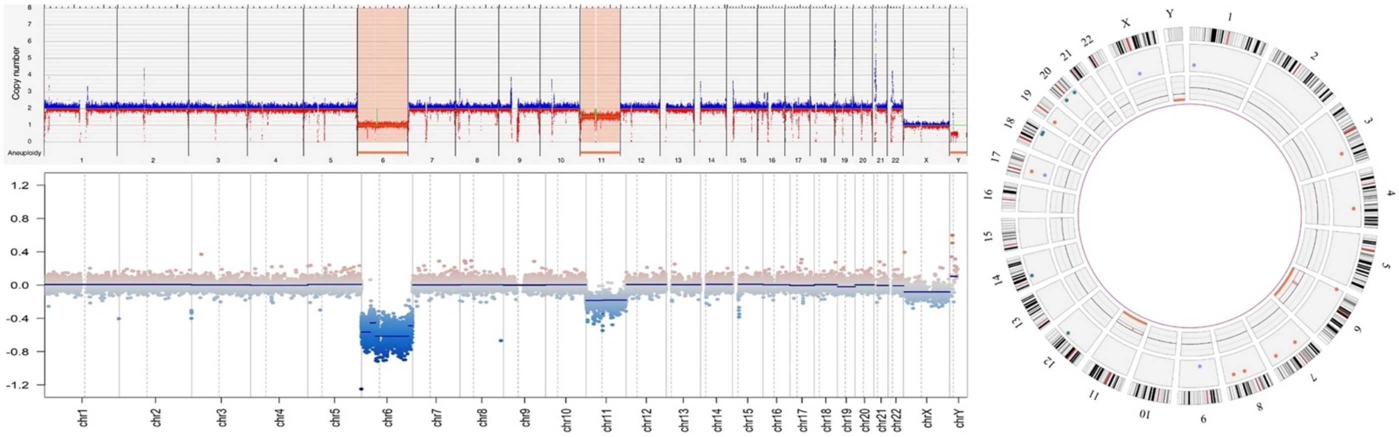

MB10t: MB, WNT-activated

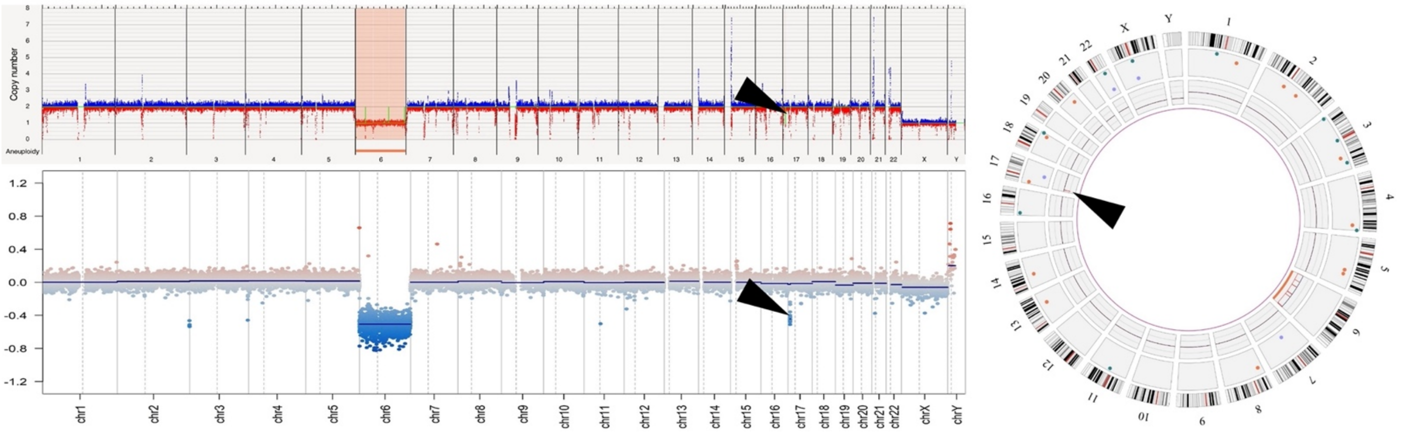

MB11t: MB, SHH-activated, subclass 4

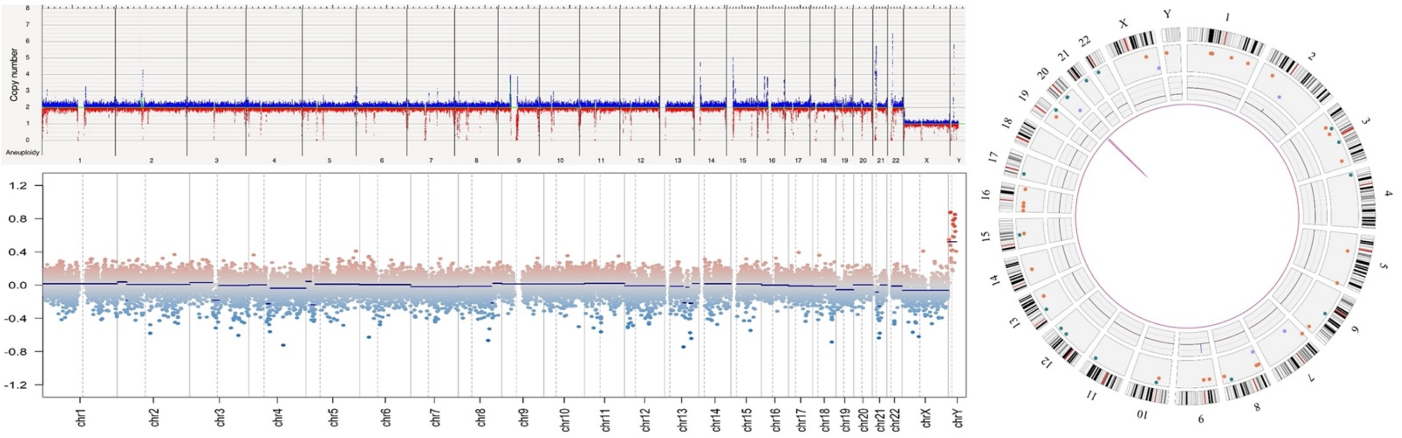

MB12t: MB, SHH-activated, subclass 1

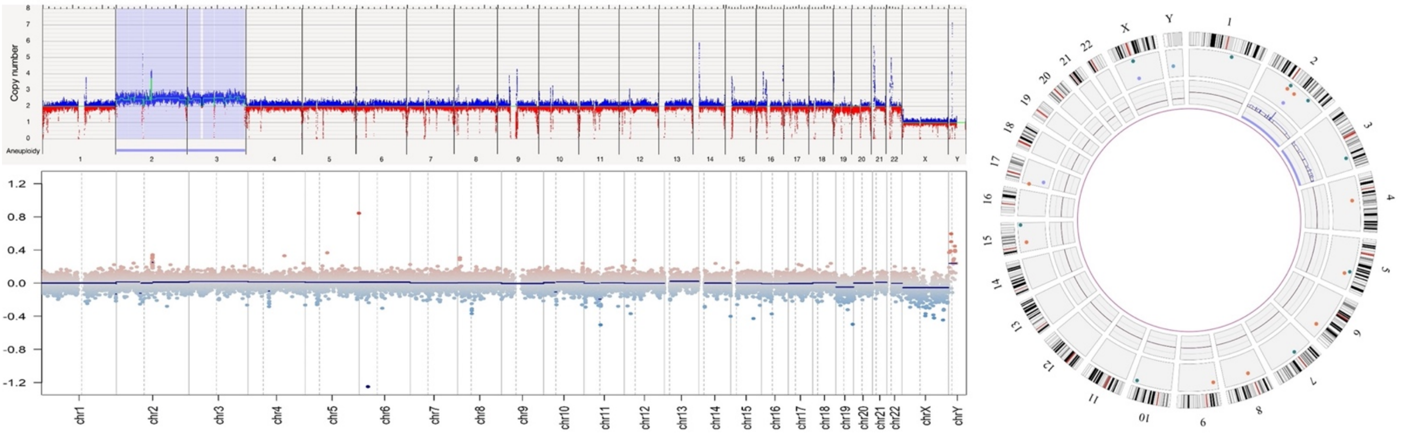

MB13t: MB, SHH-activated, subclass 3

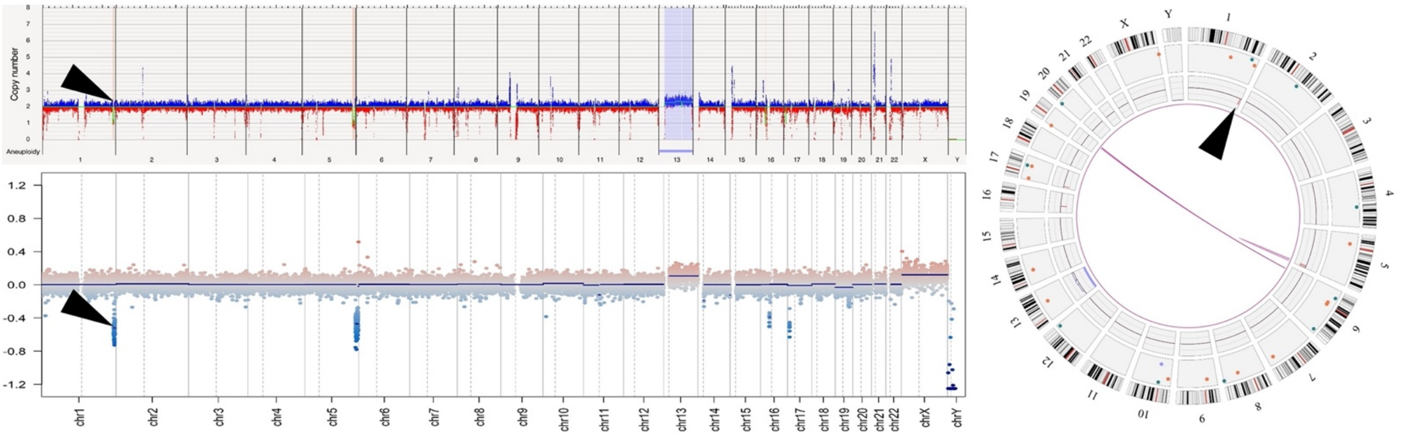

MB14t: MB, non-WNT/non-SHH, Group 4 subtype, subclass VIII

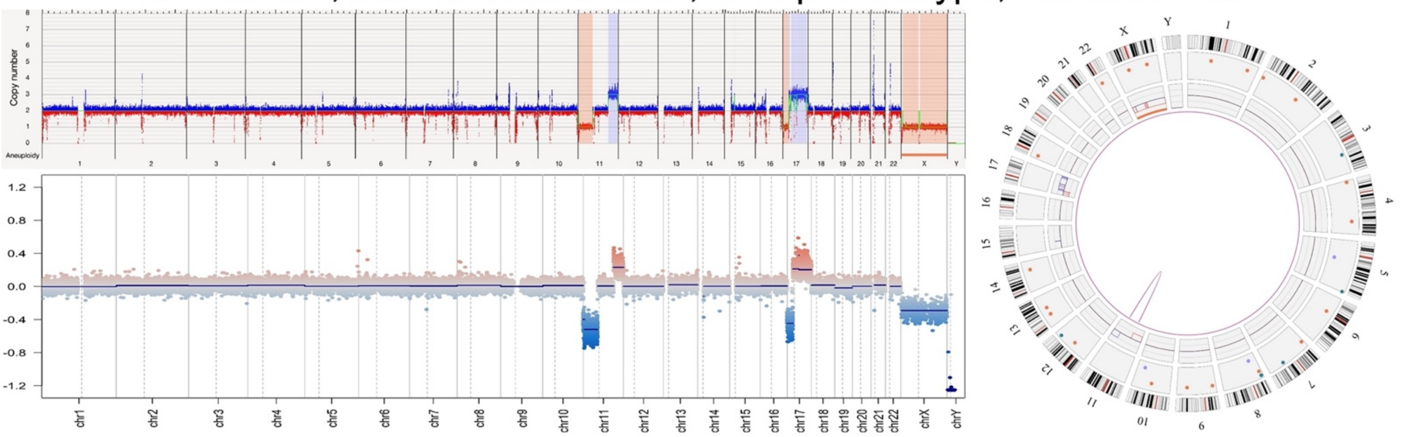

MB15t: MB, SHH-activated, subclass 3

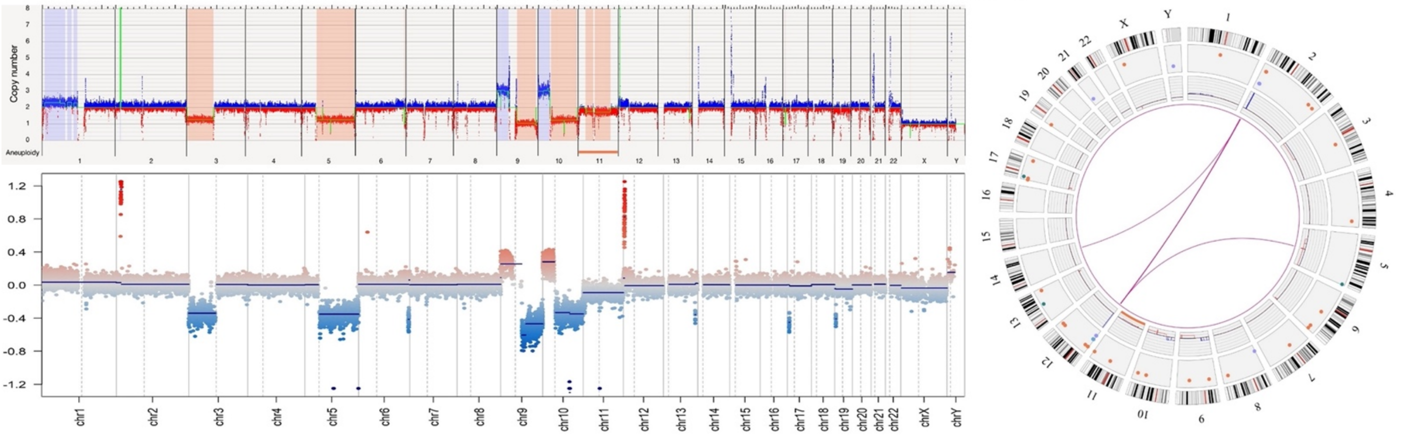

MB16t: MB, SHH-activated, subclass 1

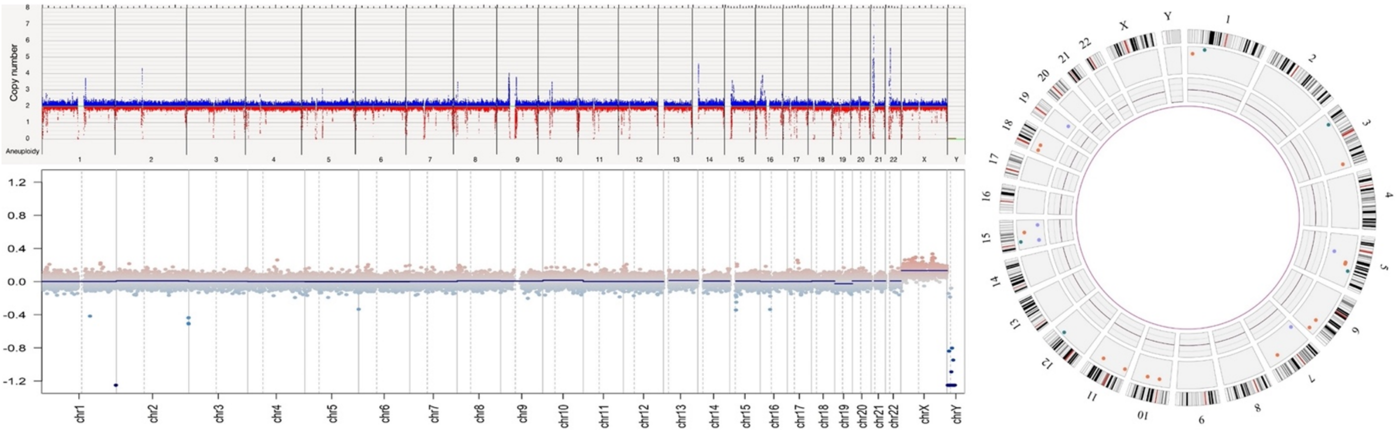

MB17t: MB, non-WNT/non-SHH, Group 4 subtype, subclass VIII

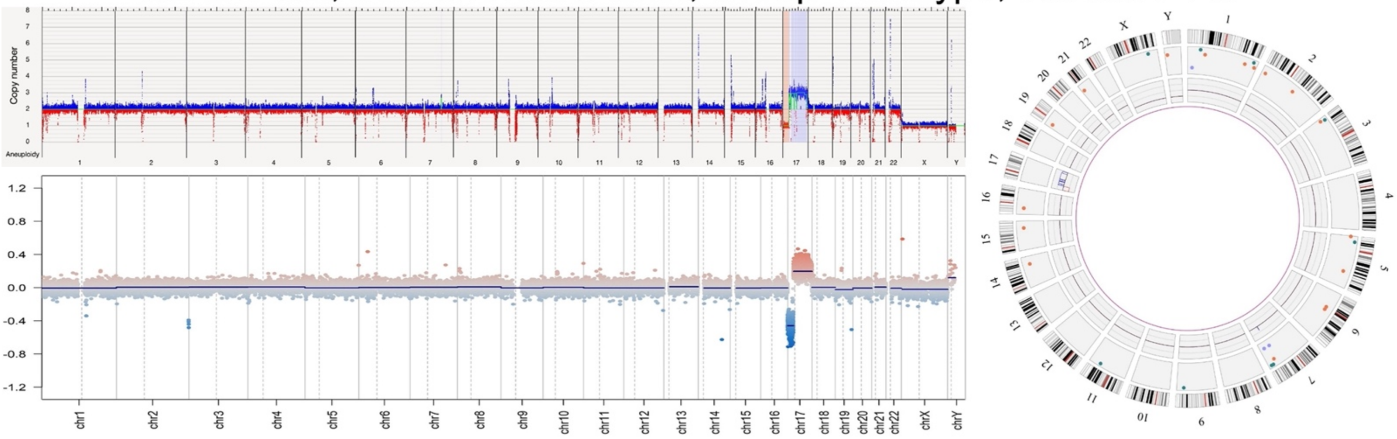

MB18t: MB, non-WNT/non-SHH, Group 3 subtype, subclass IV

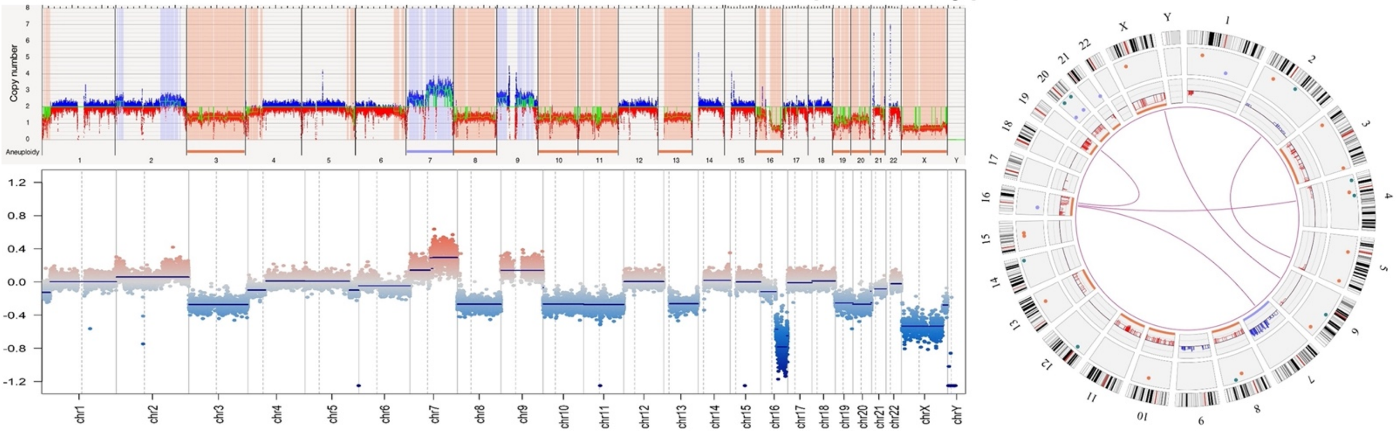

MB19t: MB, SHH-activated, subclass 4

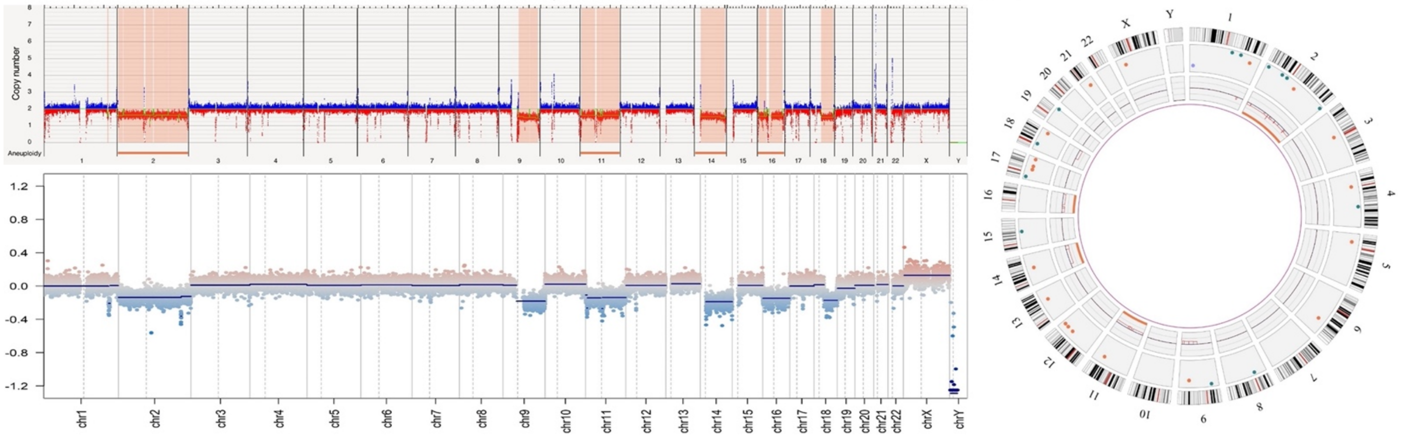

MB20t: MB, SHH-activated, subclass 2

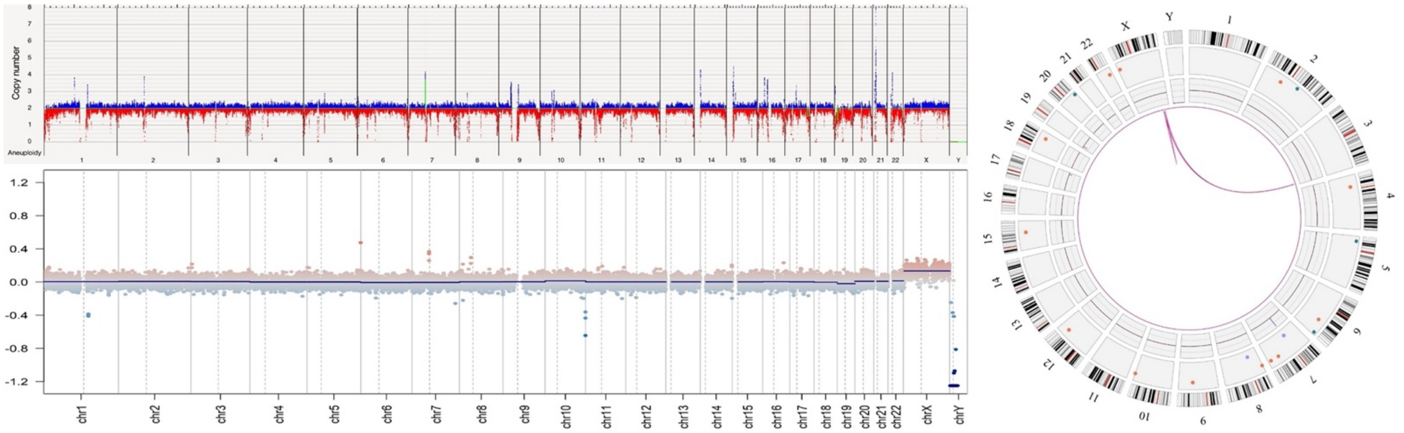

MB21t: MB, non-WNT/non-SHH, Group 4 subtype, subclass V

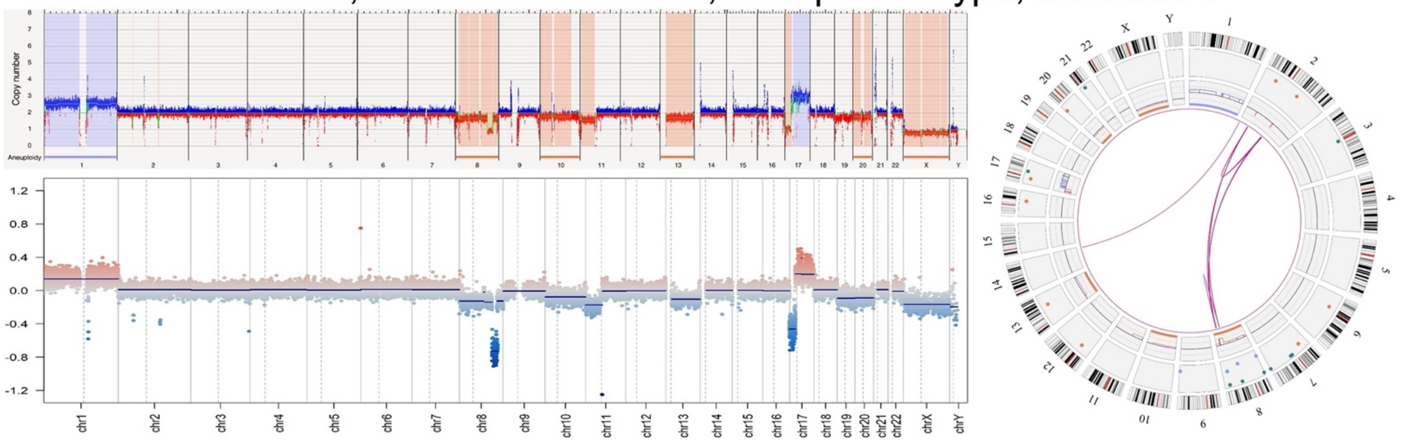

MB22t: MB, non-WNT/non-SHH, Group 4 subtype, subclass VII

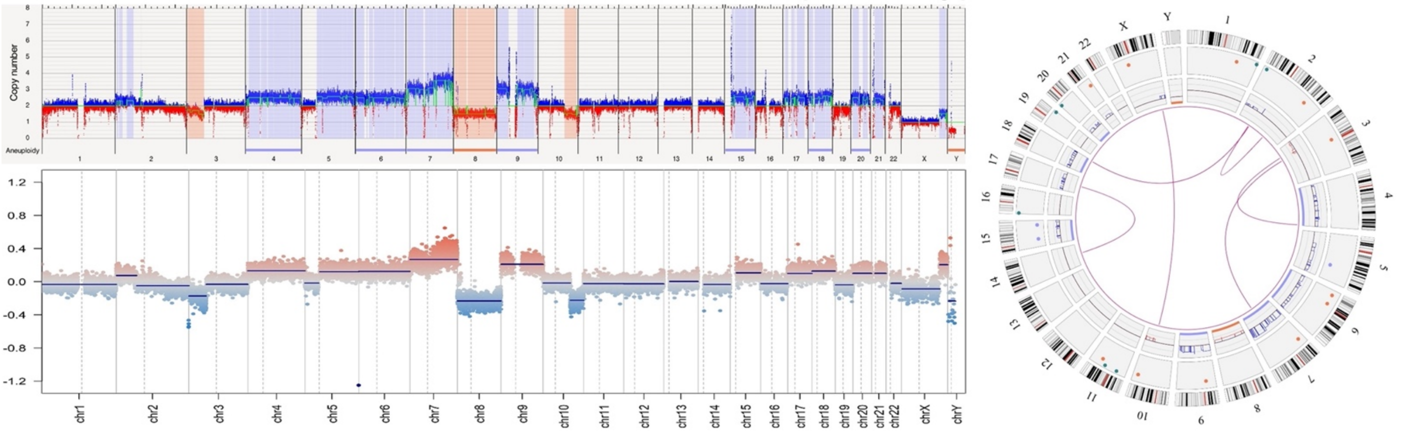

MB23t: MB, non-WNT/non-SHH, Group 3 subtype, subclass II

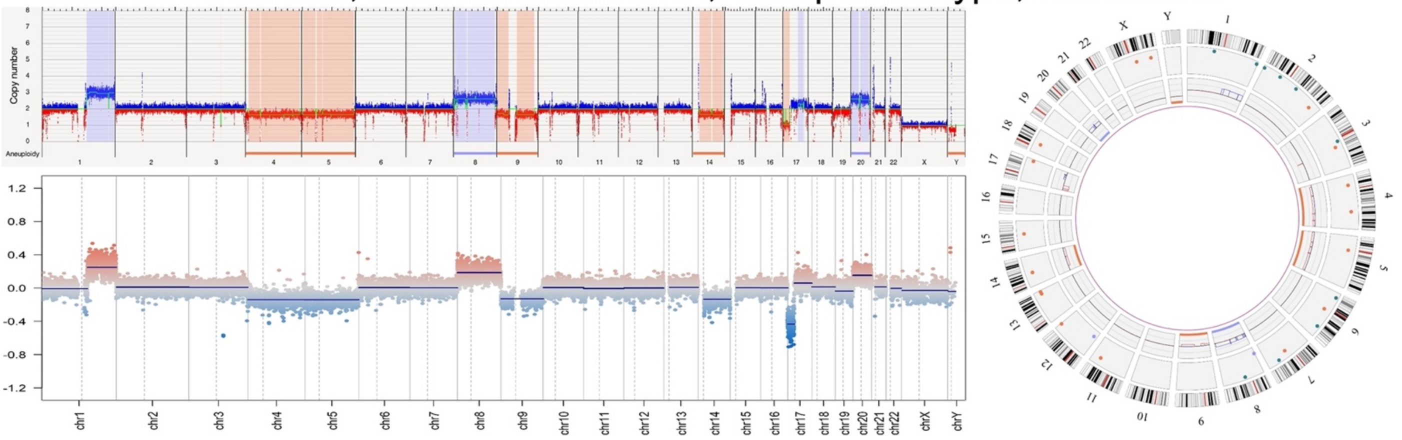

MB24t: MB, SHH-activated, subclass 2

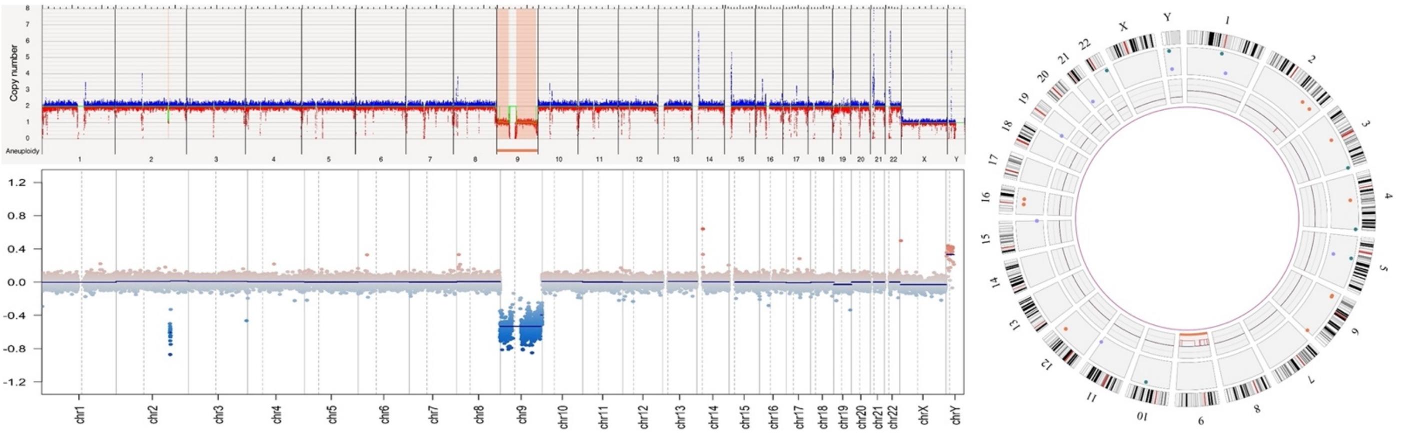

MB25t: MB, non-WNT/non-SHH, Group 4 subtype, subclass VI

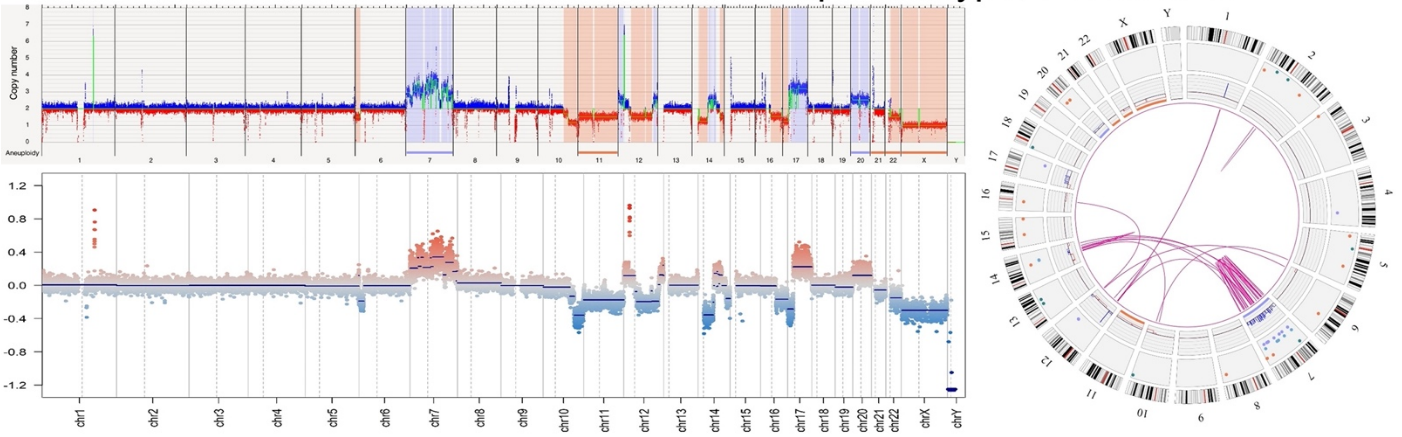

MB26t: MB, non-WNT/non-SHH, Group 4 subtype, subclass VIII

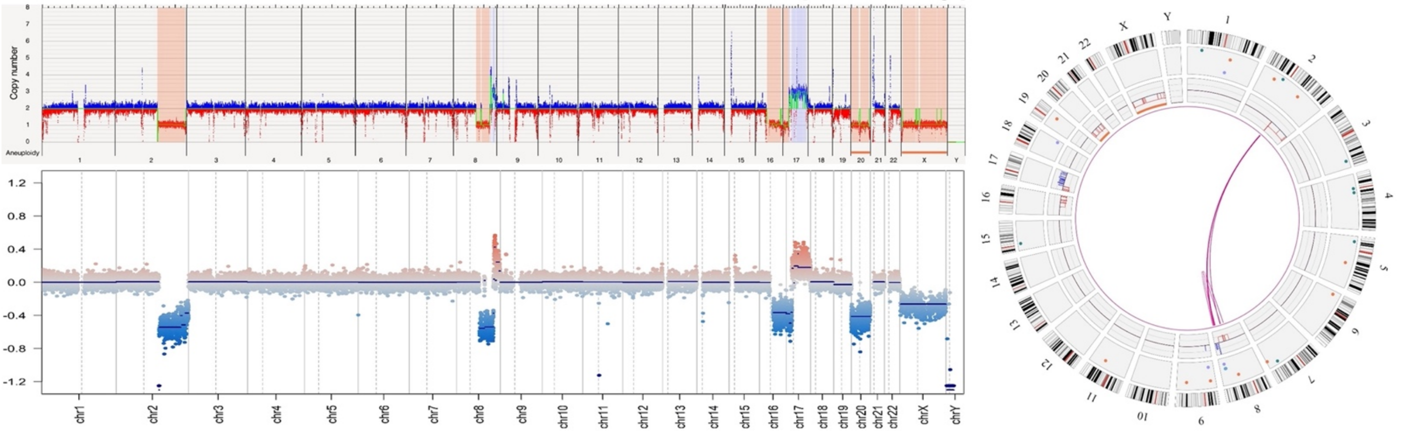

MB27t: MB, SHH-activated, subclass 1

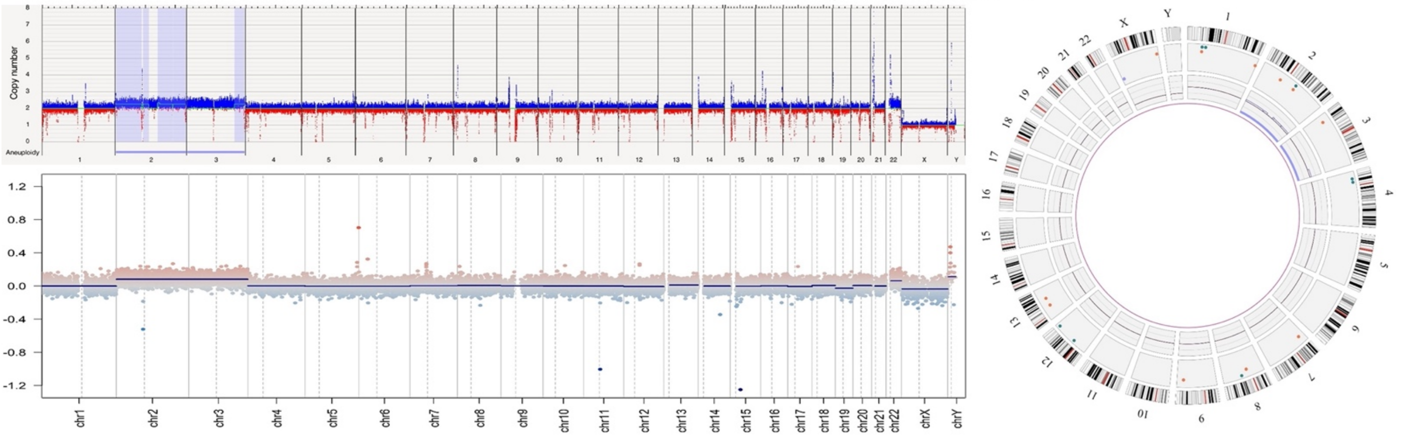

MB28t: MB, SHH-activated, subclass 2

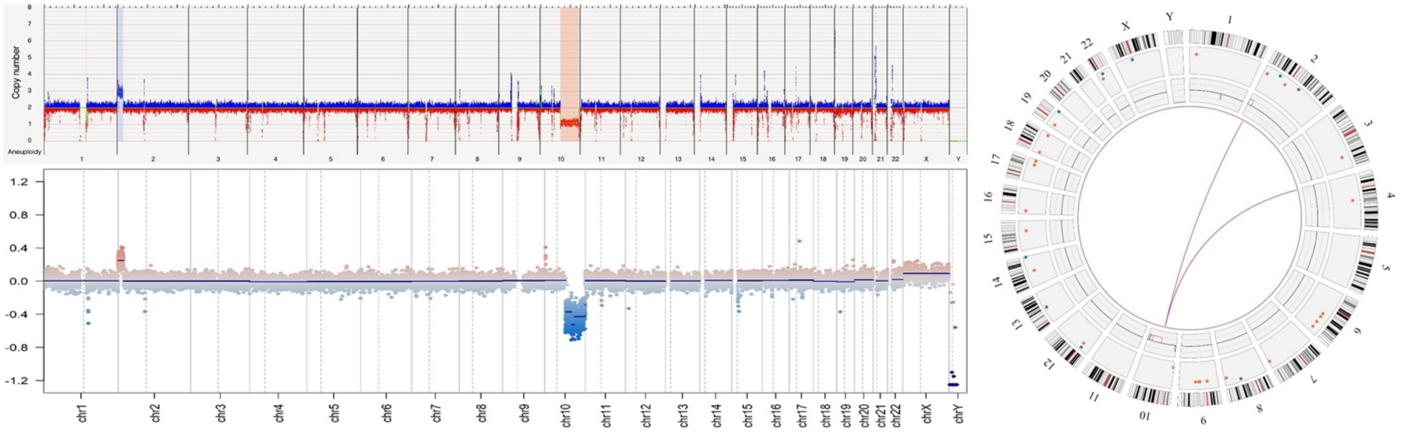

MB29t: MB, non-WNT/non-SHH, Group 3 subtype, subclass IV

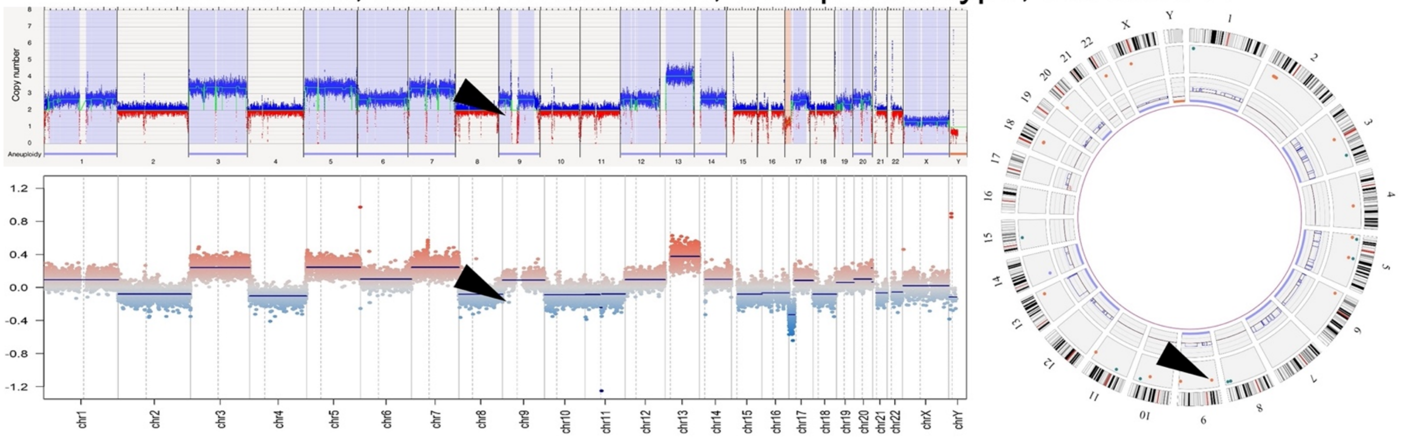

D283 Med (CVCL_1155)

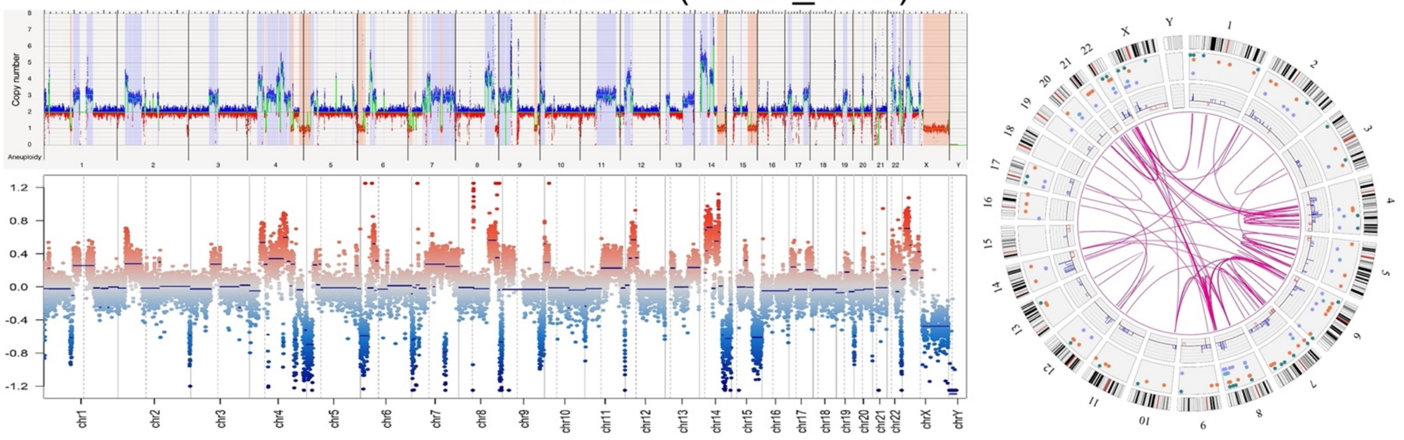

Daoy (CVCL_1167)

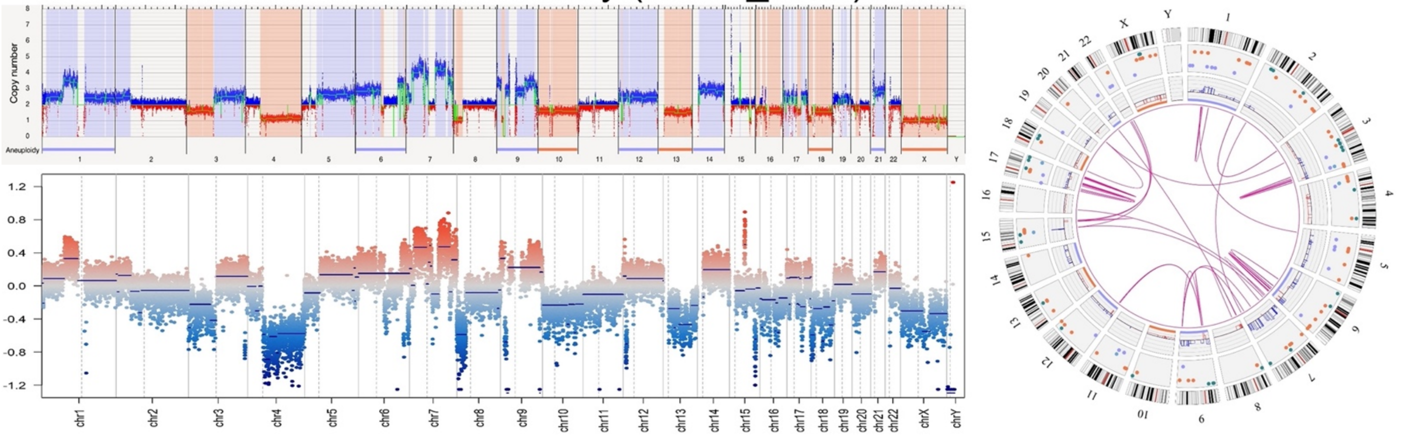

HD-MB03 (CVCL_S506): MB, non-WNT/non-SHH, Group 3 subtype, subclass II

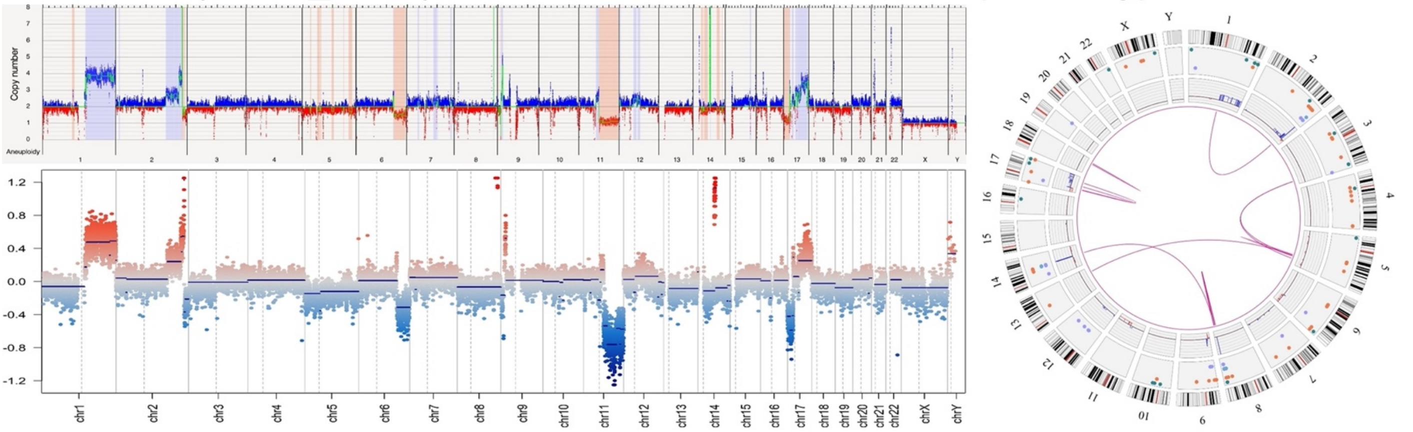

MED-MEB-8A (CVCL_M137)

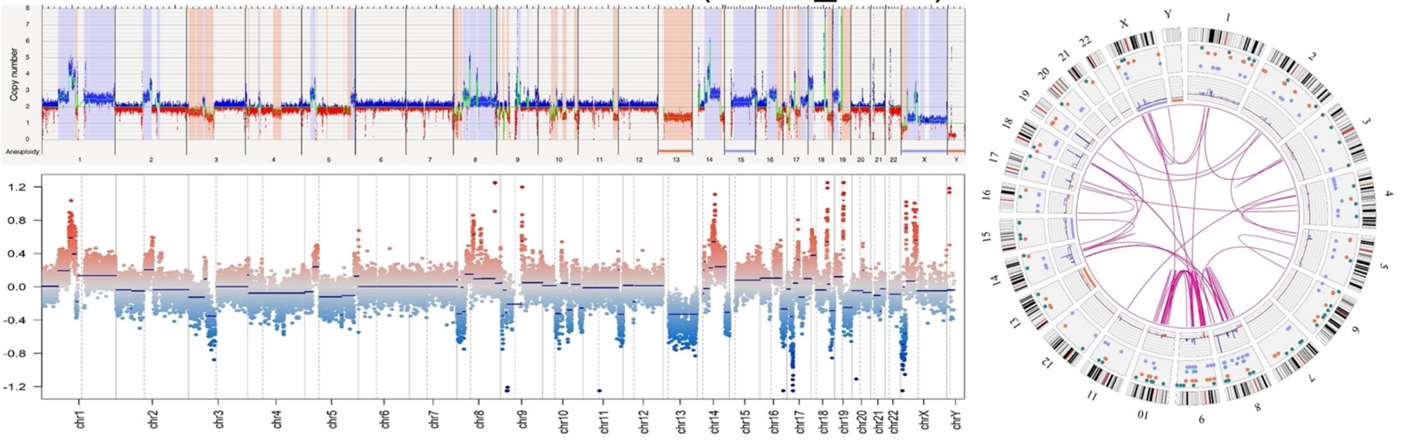

ONS-76 (CVCL_1624)

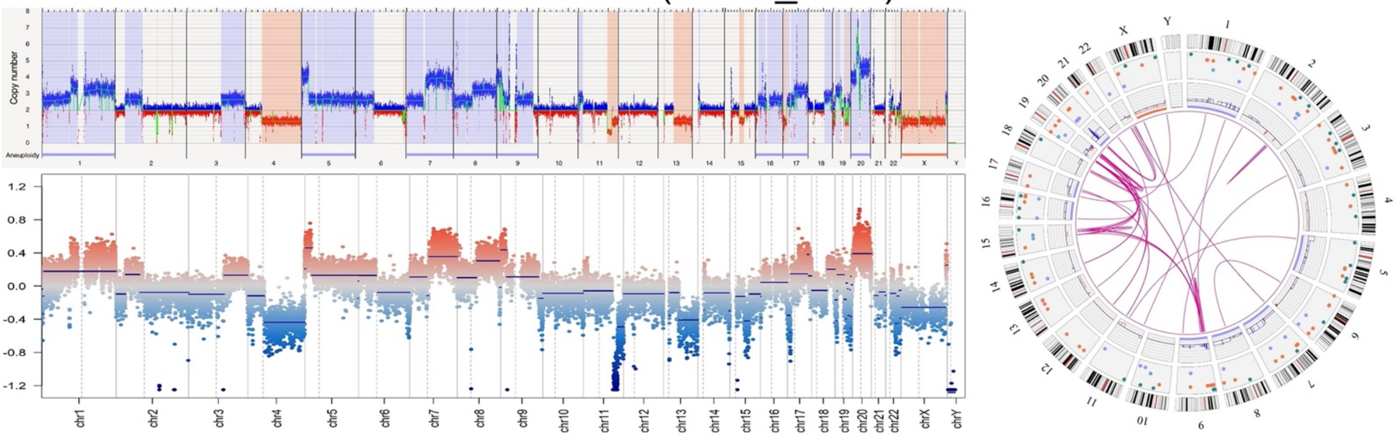

UW228-3 (CVCL_0573)

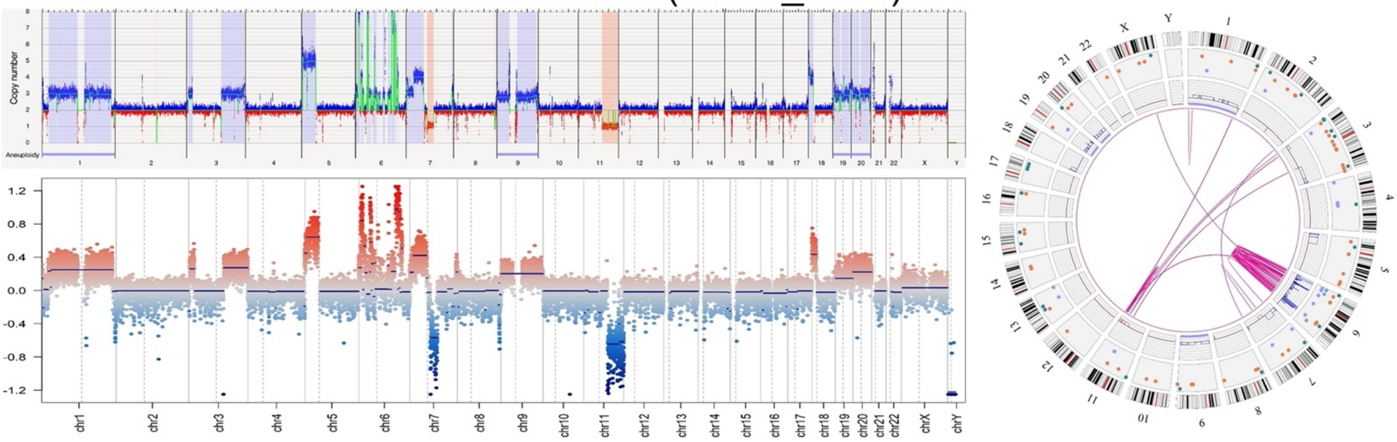

Figure S7. Landscape of CNV alterations and SVs detected by OGM in 29 MB tumors and six MB cell lines. For each case two genome-wide CNV plots (OGM: upper CNV plot, DNA methylation microarray: lower CNV plot) and one circos plot summarizing the rare SVs detected in each tumor or cell line are shown. In the OGM-derived CNV plots, chromosomal regions with copy number losses are indicated in red and regions with copy number gains are indicated in blue. *y-axis*, CN in the interval [0, 8]; *x-axis*, Aneuploidy indicators showing whole-chromosome monosomies or total losses (red) and whole-chromosome trisomies or amplifications (blue), and chr1–22 plus sex chromosomes. In the DNA methylation microarray-derived CNV plots, chromosomal regions with copy number losses are indicated in blue and chromosomal regions with copy number gains are indicated in red. *y-axis*, log2 median segment intensity in the interval [-1.2, 1.2]; *x-axis*, chr1–22 plus sex chromosomes. The circos plots show, from the outside to the center, the chromosome numbers and ideograms, SVs represented as color-coded dots (*red*, deletion; *purple*, duplication; *light-blue*, insertion; *dark-blue*, inversion), the integrated CN plot, and translocations/fusion events of complex rearrangements as connective pink arcs. Black arrowheads point to specific events discussed within the result section.

Supplementary references

1. Azatyan A, Zaphiropoulos PG (2022) Circular and Fusion RNAs in Medulloblastoma Development. Cancers 14:3134. doi: 10.3390/cancers14133134

2. Belleau P, Deschênes A, Beyaz S, Tuveson DA, Krasnitz A (2021) CNVMetrics package: Quantifying similarity between copy number profiles

3. Bionano Genomics (2020) Bionano Solve Theory of Operation: Structural Variant Calling - CG-30110, Rev G

4. Capper D, Jones DTW, Sill M, Hovestadt V, Schrimpf D, Sturm D *et al.* (2018) DNA methylation-based classification of central nervous system tumours. Nature 555:469–474. doi: 10.1038/nature26000

5. Capper D, Stichel D, Sahm F, Jones DTW, Schrimpf D, Sill M *et al.* (2018) Practical implementation of DNA methylation and copy-number-based CNS tumor diagnostics: the Heidelberg experience. Acta Neuropathol (Berl) 136:181–210. doi: 10.1007/s00401-018-1879-y

6. Dobin A, Davis CA, Schlesinger F, Drenkow J, Zaleski C, Jha S *et al.* (2013) STAR: ultrafast universal RNA-seq aligner. Bioinformatics 29:15–21. doi: 10.1093/bioinformatics/bts635

7. Ewels P, Peltzer A, Fillinger S, Patel H, Alneberg J, Wilm A *et al.* (2020) The nf-core framework for community-curated bioinformatics pipelines. Nat Biotechnol 38:276–278. doi: 10.1038/s41587-020-0439-x.

8. Forget A, Martignetti L, Puget S, Calzone L, Brabetz S, Picard D *et al.* (2018) Aberrant ERBB4-SRC Signaling as a Hallmark of Group 4 Medulloblastoma Revealed by Integrative Phosphoproteomic Profiling. Cancer Cell 34:379-395.e7. doi: 10.1016/j.ccell.2018.08.002

9. Gao B, Huang Q, Baudis M (2018) segment_liftover : a Python tool to convert segments between genome assemblies. F1000Research 7:319. doi: 10.12688/f1000research.14148.2

10. Krueger F (2019) Trim Galore. https://www.bioinformatics.babraham.ac.uk/projects/trim_galore/

11. Lawrence M, Huber W, Pagès H, Aboyoun P, Carlson M, Gentleman R *et al.* (2013) Software for Computing and Annotating Genomic Ranges. PLoS Comput Biol 9:e1003118. doi: 10.1371/journal.pcbi.1003118

12. Love MI, Huber W, Anders S (2014) Moderated estimation of fold change and dispersion for RNA-seq data with DESeq2. Genome Biol 15:550. doi: 10.1186/s13059-014-0550-8

13. Mölder F, Jablonski KP, Letcher B, Hall MB, Tomkins-Tinch CH *et al.* (2021) Sustainable data analysis with Snakemake. F1000Research 10:33. doi: 10.12688/f1000research.29032.2

14. Nicorici D, Şatalan M, Edgren H, Kangaspeska S, Murumägi A, Kallioniemi O *et al.* (2014) FusionCatcher – a tool for finding somatic fusion genes in paired-end RNA-sequencing data. doi: 10.1101/011650

15. Patro R, Duggal G, Love MI, Irizarry RA, Kingsford C (2017) Salmon provides fast and bias-aware quantification of transcript expression. Nat Methods 14:417–419. doi: 10.1038/nmeth.4197

16. R Core Team (2021) R: A Language and Environment for Statistical Computing. R Foundation for Statistical Computing, Vienna. https://www.R-project.org/

17. Sedlazeck FJ, Rescheneder P, Smolka M, Fang H, Nattestad M, Von Haeseler A *et al.* (2018) Accurate detection of complex structural variations using single-molecule sequencing. Nat Methods 15:461–468. doi: 10.1038/s41592-018-0001-7

18. Talevich E, Shain AH, Botton T, Bastian BC (2016) CNVkit: Genome-Wide Copy Number Detection and Visualization from Targeted DNA Sequencing. PLOS Comput Biol 12:e1004873. doi: 10.1371/journal.pcbi.1004873

19. Wickham H (2009) ggplot2: Elegant Graphics for Data Analysis. Springer New York, New York, NY. doi: 10.1007/978-0-387-98141-3

20. Wickham H, Averick M, Bryan J, Chang W, McGowan L, François R *et al.* (2019) Welcome to the Tidyverse. J Open Source Softw 4:1686. doi: 10.21105/joss.01686
